# Simultaneous Determination, Transfer Behaviors, Degradation, and Risk Assessment of Pesticides and Q-Marker in *Angelica sinensis* During Decoction

**DOI:** 10.3390/foods15122222

**Published:** 2026-06-19

**Authors:** Hongyan Zhang, Qiaoying Chang, Jian Li, Fuxiang Wu

**Affiliations:** 1 Key Laboratory of Pesticide and Veterinary Drug Monitoring for State Market Regulation, Lanzhou Institute for Food and Drug Control, Lanzhou 730050, China; lj237561297@163.com (J.L.); w635504150@163.com (F.W.); 2 Chinese Academy of Quality and Inspection & Testing, Beijing 102600, China

**Keywords:** pesticides, Q-markers, LC-Q-TOF/MS, *Angelica sinensis*, decoction, density functional theory, risk assessment

## Abstract

Based on liquid chromatography quadrupole time-of-flight mass spectrometry (LC-Q-TOF/MS), a high-throughput method was developed and validated for the simultaneous detection of 270 pesticides and two quality markers (Q-markers)—ferulic acid and ligustilide—in *Angelica sinensis* (*AS*) decoction. Among 50 batches of commercial samples, 15 pesticides were detected. This study dynamically monitored the effects of processing on the content of these 15 pesticides and the two Q-markers. The results showed that distinct differences were observed in the transfer behaviors of the pesticides and Q-Markers during soaking and the first and secondary boiling stages. The decoction transfer rates were calculated and incorporated to establish a risk assessment model applicable to *AS*. During the decoction, density functional theory (DFT) analysis, combined with LC-Q-TOF/MS confirmation, was employed to elucidate the thermal degradation mechanism of chlorpyrifos. DFT-based thermodynamic analysis was used to explain the significant differences in thermal loss between ferulic acid and ligustilide.

## 1. Introduction

*Angelica sinensis* (*AS*) is the dried root of the umbelliferous plant *AS* (Oliv.) Diels, which is recorded in the *Chinese Pharmacopoeia* [1]. It is mainly distributed in Gansu, Yunnan, Sichuan, and other regions in China. *AS* has a history of thousands of years of application, first recorded in Shennong’s *Classic of Materia Medica* compiled during the Han Dynasty [2]. In China, *AS* is also a substance of medicine–food homology (SMFH), which was included in the list of SMFH in 2019 [3]. For a long time, *AS* has been used to treat asthenia, nourish blood, promote blood circulation, relieve pain, and moisten the intestines, as well as to treat menstrual disorders and amenorrhea in women [4].

Traditional Chinese medicine (TCM) decoction is the primary dosage form of prepared TCM pieces and one of the most widely used clinical formulations. During preparation, the active constituents from the herbal pieces become molecularly dispersed in the decoction under boiling conditions, enabling rapid transportation to target tissues for efficient absorption by the human body. However, owing to the increasing scarcity of wild medicinal resources and a lack of effective regulations in artificial cultivation, pesticides are heavily sprayed to boost yields. To ensure therapeutic efficacy and safety, it is critical to understand the transformation of pesticide residues, as well as bioactive compounds, during the decoction of *AS*. This knowledge enables an effective reduction in pesticide contamination, while maximizing the extraction of pharmacologically active components. Ferulic acid and ligustilide are recognized as quality markers (Q-markers) for *AS*. According to the *Chinese Pharmacopoeia*, the following quality standards apply: ferulic acid content ≥ 0.05% and total volatile oils ≥ 0.4% [1], with ligustilide accounting for 40–70% of the volatile oils. The current studies have the following limitations: (I) Most of the current research has primarily investigated pesticide residue detection methods for raw TCM pieces. Several sample purification techniques have been developed, including QuEChERS [5,6,7], solid-phase extraction [8,9], fluorescent probes [10], electrochemiluminescence [11], and gel permeation chromatography [12]. These methods effectively extract pesticide residues from TCM pieces. However, pieces undergo boiling during decoction; pesticides and other chemical components may undergo thermal degradation, transformation, or volatilization. Thus, pesticide residue data from raw TCM pieces cannot directly reflect residue levels in the final decoction. (II) Existing analytical methods for TCM typically study bioactive compounds and contaminants in isolation, lacking an integrated and synergistic detection evaluation system. Liquid chromatography and gas chromatography have been used for the quantification of bioactive compounds such as ferulic acid and ligustilide, whereas liquid chromatography–mass spectrometry has been used to detect pesticide residues in the *Chinese Pharmacopoeia* [1]. This fragmented research paradigm presents significant limitations. It may overlook potential interactions between medicinal components and contaminants during decoction. (III) Existing studies on pesticide transfer behaviors have predominantly focused on dissipation half-life, processing factors, and transfer rates during different processes, including pressing [13], processing [14,15,16], cooking [17], removal effect [18], and brewing [19,20,21,22]. Furthermore, the relevant physicochemical parameters (water solubility (WS), log *k_ow_*, vapor pressure (VP), Henry, and thermodynamic values) affecting pesticide transfer behavior are analyzed [16,22,23]. However, the aforementioned studies solely focused on calculating the total transfer rate of pesticides after processing, neglecting the dynamic monitoring of pesticides during the processing stage. Few studies report the transfer behavior of pesticide residues in TCM during soaking and the first and secondary boiling stages.

Our study was based on liquid chromatography quadrupole time-of-flight mass spectrometry (LC-Q-TOF/MS) and a high-resolution mass spectrometry database containing nearly a thousand pesticides. The current research intends to: (I) establish a simultaneous analytical method for 270 pesticides and two Q-markers (ferulic acid and ligustilide) in *AS* decoction. The 270 pesticides were selected from those prone to excessive residue in routine supervision. The Q-markers were derived from the characteristic marker components of *AS* specified in the *Chinese Pharmacopoeia*; (II) quantify and analyze the changes of the 15 detected pesticides and two Q-markers during soaking, first boiling, and secondary boiling; (III) investigate the decoction transfer rate regularity of the 15 pesticides and two Q-markers; (IV) explain the degradation pathway of chlorpyrifos and divergent thermal losses of the two Q-markers by DFT. The present work establishes an integrated strategy combining high-throughput multi-residue screening, dynamic processing monitoring, component transfer rate quantification, and theoretical thermodynamic calculation. This integrated analytical and computational approach effectively deepens the current understanding of substance transformation and safety changes during *AS* decoction preparation and provides a more comprehensive methodological and theoretical reference for the safety evaluation and standardized processing of TCM decoctions.

## 2. Materials and Methods

### 2.1. Materials and Reagents

An Agilent 1290 Infinity II LC-6545 Q-TOF/MS liquid chromatograph time-of-flight mass spectrometer with a dual-injection electrospray ionization source (Agilent, Santa Clara, CA, USA) was used. Pesticide standards (Alta Technology Co., Ltd., Tianjin, China), ferulic acid and ligustilide standards (National Institutes for Food and Drug Control, Beijing, China), chromatographical grade formic acid (Fisher, Pittsburgh, PA, USA, 99%), ammonium acetate (Fisher, Pittsburgh, PA, USA, 97%), acetonitrile (ACN) (Fisher, Pittsburgh, PA, USA, 99.9%), analytical grade glacial acetic acid (Longxi Science, Longxi, China, 99.5%), anhydrous sodium acetate and anhydrous magnesium sulfate (Tianjin Cameo, Tianjin, China, 99.5%); Oasis PRiME HLB SPE cartridge (200 mg/6 cc, Waters, Milford, CT, USA), the dispersed extraction sorbents [anhydrous MgSO_4_, C18, primary secondary amine (PSA), silica gel, and graphitized carbon] were purchased from Agilent Technology Co., Ltd. (Beijing, China).

### 2.2. Preparation of Standard Solution

Precisely weighed standards were dissolved in methanol in 10 mL brown volumetric flasks to prepare stock solutions and stored away from light at −18 °C. The stock solutions were diluted with methanol to yield working solutions with the desired concentrations and kept at 4 °C.

### 2.3. Sample Preparation

#### 2.3.1. *Angelica sinensis* Pieces

Fifty batches of *AS* samples were gathered from medical facilities, trading enterprises, retail pharmacies and individual farmers across Lanzhou, Dingxi and Longnan in Gansu Province. Before sample preparation, all samples were crushed and sieved using a 0.25 mm sieve for further analysis.

#### 2.3.2. *Angelica sinensis* Decoction

Referring to the official standards for Chinese medicinal decoction in medical institutions [24], 50 g of astragalus slices were soaked in 500 mL water for 50 min, then boiled vigorously at 180–200 °C and simmered gently at 120–150 °C for 60 min to obtain the first decoction. The herbal residues were re-decocted with 250 mL hot water for another 60 min to prepare the second decoction. The two decoctions were mixed and concentrated to 200 mL.

#### 2.3.3. Positive Sample

Common methods for preparing positive samples include field spraying, laboratory immersion, and laboratory spraying. Although field spraying is recognized as an ideal spiking method, it possesses inherent limitations. Specifically, for highly toxic pesticides, field spraying trials may result in irreversible environmental contamination. Furthermore, the unpredictability of field conditions complicates the precise control of pesticide spiking concentrations, which directly compromises the accuracy and reliability of experimental results. Therefore, a laboratory-based approach was adopted for the preparation of positive samples. During the sample preparation process, methodologies for the preparation of pesticide residue-positive samples from ginseng, vegetables, and tea were referenced [25,26,27]. In light of these research findings, to ensure that the experimental data could more accurately reflect real-world conditions, positive samples were prepared by spraying pesticides onto dried specimens. This approach also facilitates the simulation of actual pesticide detection concentrations in practical application scenarios.

First, 200 g of *AS* powder was evenly spread in a flat-bottomed container. An appropriate volume of the mixed standard solution (containing 15 detected pesticides) was transferred to the sample. The solution was thoroughly dissolved and diluted with 200 mL of methanol. It was then evenly sprayed onto the sample powder in three applications with continuous stirring to ensure complete mixing. After the organic solvent fully evaporated, the prepared positive sample was placed in a brown glass bottle. The sample was vigorously shaken for 12 h and then refrigerated at 4 °C in the dark.

### 2.4. Sample Pretreatment

#### 2.4.1. *AS* Pieces

Exactly 3 g of the test sample was accurately weighed and transferred into an 80 mL polystyrene centrifuge tube. Subsequently, 15 mL of 5% formic acid aqueous solution was added to the tube, followed by thorough mixing, and the mixture was allowed to stand for 30 min. Thereafter, 30 mL of acetonitrile (ACN) was added, mixed homogeneously, and the mixture was shaken at 300 r/min for 10 min. A total of 7.5 g of a powder mixture composed of anhydrous magnesium sulfate and anhydrous sodium acetate (4:1, m/m; corrected from *v*/*v*, as this ratio refers to the proportion of solid powders) was added immediately, mixed thoroughly, and shaken at 300 rpm for 5 min. The mixture was then placed in an ice-water bath for 10 min prior to centrifugation at 4200 r/min for 5 min. The resulting supernatant was diluted appropriately and filtered through a 0.22 μm filter membrane for the direct detection of ferulic acid and ligustilide via LC-Q-TOF/MS analysis. Separately, 10 mL of the supernatant was pipetted into another tube and concentrated to a final volume of 2 mL. The concentrated solution was loaded onto an HLB solid-phase extraction column; after the entire volume of the solution had completely passed through the column, the column was eluted twice with 3 mL of methanol each time. The eluates from the two elution steps were combined and concentrated under a gentle nitrogen stream. The resulting residue was dissolved and diluted to 1 mL with a methanol/water mixture (3:2, *v*/*v*), vortex-mixed thoroughly, and then filtered through a 0.22 μm filter membrane for subsequent LC-Q-TOF/MS analysis.

#### 2.4.2. *AS* Decoction

For the detection of pesticide residues in *AS* decoctions, 10 mL of the decoction was mixed with 10 mL of acetonitrile (ACN) containing 1% acetic acid, and the tube was shaken at 300 rpm for 10 min. Subsequently, 10 g of a mixed powder of anhydrous magnesium sulfate and anhydrous sodium acetate (4:1, m/m) was added, immediately mixed, and shaken at 300 rpm for 5 min. The mixture was then placed in an ice-water bath for 10 min before centrifugation at 4200 rpm for 5 min.

The supernatant was appropriately diluted and filtered through a 0.22 μm filter membrane for the direct detection of ferulic acid and ligustilide via LC-Q-TOF/MS analysis. Nine milliliters of the supernatant was transferred into an advanced-equipped dispersive solid-phase extraction (d-SPE) purification tube, which contained 900 mg of anhydrous magnesium sulfate, 300 mg of primary secondary amine (PSA), 300 mg of C18, 300 mg of silica gel, and 90 mg of graphitized carbon. The sample was vortex-mixed thoroughly, shaken at 300 rpm for 10 min, and centrifuged at 4200 rpm for 5 min for purification. A 5 mL aliquot of the purified supernatant was concentrated under a gentle nitrogen stream. The resulting residue was dissolved and diluted to 1 mL with ACN, and then filtered through a 0.22 μm filter membrane for subsequent LC-Q-TOF/MS analysis.

### 2.5. Instrumentation and Instrument Conditions

Agilent 1290 Infinity II L C-6545 Q-TOF/MS liquid chromatograph flight time mass spectrometer with dual jet electrospray ionization source (Agilent Technologies, Santa Clara, CA, USA); chromatographic column: SB-C18 (2.1 mm, 100 mm × 3.5 μm), column temperature: 40 °C; mobile phase: phase A was 0.1% formic acid aqueous solution, and phase B was ACN; flow rate: 0.4 mL/min. Gradient elution procedure: 0–3 min, 1–30% B; 3–6 min, 30–40% B; 6–9 min, 40% B; 9–15 min, 40–60% B; 15–19 min, 60–90% B; 19–23 min, 90% B; 23–23.01 min, 90–1% B; 23.01–27.01 min, 1% B. Injection volume: 5 μL Dual AJS ESI source; scanning mode: positive ion full scan; full scanning range: m/z 50–1000; capillary voltage: 4000 V; atomizing gas: nitrogen; atomizing gas pressure: 0.14 MPa; sheath gas temperature: 375 °C; sheath gas flow rate: 11.0 L/min; dry gas flow rate: 12.0 L/min; dry gas temperature: 325 °C; breaking voltage: 145 v. All ions MS/MS mode conditions: at 0 min, the collision energy was 0 eV; at 0.5 min, the collision energy was 0, 15, and 35 V.

### 2.6. Construction of Accurate Quality Database

The LC-Q-TOF/MS system was operated in MS mode to conduct full-scan analysis. Qualitative software (B.10.0) was utilized to extract molecular ion peaks to determine the retention time and ionization forms ([M + H]^+^, [M + NH_4_]^+^, and [M + Na]^+^) of each pesticide. The names, molecular formulas, and accurate masses of all target pesticides were imported into the MassHunter Personal Compound Database and Library (PCDL) management software to establish a primary accurate mass database for the pesticides. Subsequently, the LC-Q-TOF/MS system was switched to targeted MS/MS mode for secondary scanning. The accurate mass of precursor ions, retention time, and four distinct collision energies (10, 20, 30, and 40 eV) obtained from the primary mass spectrometry analysis were input into the system. The fragment ion mass spectra of the precursor ions of each pesticide under different collision energies were collected and imported into the PCDL software (B.08), thus enabling the establishment of a secondary accurate mass database and spectral library for the pesticides.

To ensure the accuracy of the established database, the molecular formulas and structural formulas of the target pesticides were calibrated via mol map, which was imported using MassHunter Molecular Structure Correlator software (B.10.00). The software automatically calculated theoretical fragment ions and simulated the potential cleavage modes of the compounds. By comparing the accurate masses of the theoretical fragment ions with those of the actual fragment ions, the fragment ions with higher system scores were selected to characterize the pesticides in the library. The PCDL database and mass deviations of 270 pesticides and two Q-markers are presented in Supplementary Table S1.

### 2.7. Calculation of Transfer Rate and Half-Life

The transfer rate was determined using Equation (1), and a prediction model for the transfer rate was created using linear fitting.

(1) $$T(\%)=\frac{C1\times V}{C2\times M}\times 100$$

In the equation above, T (%) stands for the rate of transfer of compounds, C1 for the concentration in the decoction of *AS* (μg/L), C2 for the concentration in the piece of *AS* used for decocting (mg/kg), V for the volume of the decoction of *AS* (mL), and M for the mass (g) of *AS* used for decocting.

The dissipation rate and half-life (t_1/2_) were calculated as the following equations:

(2) $$C_{t}=C_{0}e^{-\mathrm{kt}}$$

(3) $$t_{1/2}=\frac{\ln2}{k}$$

C_t_ was the concentration of pesticide residue at time t, C_0_ was the initial concentration, and k was the rate constant.

### 2.8. Dietary Intake Risk Assessment

The hazard index (HI) method recommended by the European Food Safety Authority (EFSA) is commonly used to evaluate the chronic and acute dietary intake risk of pesticide residues in food [28,29,30]. For *AS*, we have innovatively introduced the transfer rate to establish a risk assessment model, which makes the risk assessment results more practically applicable.

Risk assessment method for chronic dietary intake:

(4) $$\mathrm{EDI}=\frac{C\times \mathrm{IR}\times t}{\mathrm{BW}}$$

EDI is the daily dietary exposure level (mg/kg bw·d); C is the average content of pesticide residues (mg/kg); IR is the average daily consumption (kg/d) of *AS* by the consumer population. The 2025 edition of the *Chinese Pharmacopoeia* stipulates that the dosage of *AS* is 0.006~0.012 kg. In this study, the median consumption of 0.009 kg was taken as the average consumption; t is the transfer rate of pesticides after decoction extraction (%); BW is the average weight of the consumer population, with an average weight of 60 kg for adults and 15 kg for children.

Risk assessment method for acute dietary intake:

(5) $$\mathrm{ESTI}=\frac{\mathrm{LP}\times \mathrm{HR}\times t}{\mathrm{BW}}$$

ESTI stands for short-term dietary intake (mg/kg bw·d); LP is a large portion meal of *AS* (kg/d), and the *Chinese Pharmacopoeia* 2025 edition stipulates that the maximum dosage of *AS* pieces is 0.012 kg [1]; HR is the highest residual level (mg/kg); t is the transfer rate of pesticides after decoction extraction (%); BW is the average weight of the population (kg), with an average weight of 60 kg for adults and 15 kg for children.

Cumulative dietary intake risk assessment:

chronic dietary intake risk hazard index

(6) $$\mathrm{HI}=\sum_{i=1}^{n}{\mathrm{HQ}}_{c}=\sum_{i=1}^{n}\frac{{\mathrm{EDI}}_{i}}{{\mathrm{ADI}}_{i}}$$

acute dietary intake risk hazard index

(7) $$\mathrm{HI}=\sum_{i=1}^{n}{\mathrm{HQ}}_{a}=\sum_{i=1}^{n}\frac{{\mathrm{ESTI}}_{i}}{{\mathrm{ARfD}}_{i}}$$

HQ_c_ represents the chronic hazard quotient, and ADI is the daily allowable intake (mg/kg bw·d); HQ_a_ is the hazard quotient of exposure to a certain chemical in large meals; ARfD is the acute reference dose (mg/kg bw·d), derived from JMPR [31]. If HI ≤ 1, it indicates that the dietary exposure risk is at a controllable level, and the smaller the value, the lower the risk; if HI > 1, it indicates that the dietary exposure risk exceeds the controllable level, and the higher the value, the greater the risk.

### 2.9. Density Functional Theory (DFT) Calculations

In addition to the experimental characterization, DFT calculations were further used to help reveal the pyrolysis mechanism of pesticide chlorpyrifos by bond dissociation energy (BDE). Moreover, DFT was employed to explain the thermal loss difference between the two Q-markers via thermodynamic calculations. Theoretical calculations were performed using Gaussian 09 software, employing DFT with the B3LYP/6-31G(d) basis set for structural optimization and frequency calculations. No virtual frequencies were observed during frequency calculations following structural optimization. Energy calculations were performed using the B3LYP/6-311G(d) basis set.

### 2.10. Statistical Analysis

All measurements were carried out in triplicate. SPSS Statistics software 27.0.1 (IBM Software Inc., New York, NY, USA) was used for the one-way analysis of variance (ANOVA) and *t*-test. *p* < 0.05 was considered statistically different.

### 2.11. Methodological Validation

#### 2.11.1. Pesticides

In accordance with the specified requirements of the European Union guideline document SANTE/11312/2021 [32], the optimized conditions were employed to verify the limit of detection (LOD), the limit of quantification (LOQ), linear relationship, matrix effect (ME), recovery, relative standard deviation (RSD), and within-laboratory reproducibility (RSDwR) of 270 pesticides in *AS* decoctions. The lowest concentration at which the extracted ion chromatogram (EIC) peak corresponding to the exact mass of the target analyte can be obtained was defined as the LOD. The 270 pesticides were fortified at four concentration levels, namely 10, 20, 30, and 15 μg/L, and the minimum fortified level that met the recovery and precision performance criteria (recovery range of 70–120% and RSD ≤ 20%) was defined as the LOQ. Mixed standard working solutions with different concentrations were added to six blank *AS* decoctions to establish matrix-matched standard curves at concentrations of 5, 10, 20, 30, 50, and 100 μg/L. The matrix effect (ME) was calculated using the slopes of the solvent standard curve and the matrix-matched standard curve of *AS* decoctions. The recovery and RSD of the 270 pesticides were determined at three fortified levels (LOQ, 2× LOQ, and 10× LOQ), with five replicates performed for each fortified concentration. The RSDwR of the method was evaluated through intra-laboratory comparisons conducted by different analysts at different time points, with the spiking level set at 10× LOQ and five replicates for each test. The analytical results are reported in Supplementary Table S2.

#### 2.11.2. Q-Markers

Methodological validation of ferulic acid and ligustilide was conducted in accordance with the analytical method validation guidelines specified in the *Chinese Pharmacopoeia* (2025 edition) 9101.

##### Linearity, LOQ and LOD

The standard solution was precisely added to the sample for preparation, and matrix-matched standard curves for ferulic acid (0.5, 1, 2, 3, 5, and 10 mg/L) and ligustilide (0.25, 0.5, 1, 2, 5, and 10 mg/L) were established for quantitative determination. The standard curves were plotted with the horizontal coordinate (X) as the detected concentration minus the background concentration of ferulic acid and ligustilide, and the vertical coordinate (Y) as the peak area. The linear regression equations obtained were Y = 14,368X − 291.3 (R^2^ = 0.9968) for ferulic acid and Y = 21,101X + 1404.2 (R^2^ = 0.9998) for ligustilide. The LOQ of the method was defined as the fortified concentration of each analyte at a signal/noise ratio (S/N) ≥ 10, with the LOQs of ferulic acid and ligustilide being 0.5 mg/L and 0.25 mg/L, respectively. The LOD was defined as the fortified concentration at a signal/noise ratio (S/N) ≥ 3, with the LODs of ferulic acid and ligustilide being 0.2 mg/L and 0.08 mg/L, respectively.

##### Accuracy and Precision

Precisely 10 mL of *AS* decoction was accurately weighed. Standard solutions of ferulic acid and ligustilide were added at concentrations of 0.060 g/L and 0.5 g/L, respectively, with six parallel samples prepared for each analyte. All samples were pretreated in accordance with the preparation method described in Section 2.4.2. The analytical results are presented in Table 1, with average recoveries of 96.9% for ferulic acid and 95.4% for ligustilide, and RSD of 2.7% and 2.2% (n = 6), respectively. Additionally, RSDwR of the method was 3.2% and 2.6% (n = 6), conducted by different analysts at different time points, using the aforementioned spiking levels with six replicates for each test. These results indicate that the established method exhibits excellent reproducibility.

## 3. Results and Discussion

### 3.1. Optimization of the Pretreatment Method

#### 3.1.1. *Angelica sinensis* Pieces

Sample preparation was conducted in accordance with the established methodology of our team [9]. Methodological validation of 270 pesticides and two Q-markers is presented in Supplementary Table S3.

#### 3.1.2. *Angelica sinensis* Decoction

##### Extraction

The samples were placed in 50 mL centrifuge tubes, then ACN (1% acetic acid) was acidified and vortexed. The anhydrous magnesium sulfate and anhydrous sodium acetate were added, mixed and centrifuged in an oscillator. Comparison of the volume of the sample, ACN (1% acetic acid), the amount of anhydrous magnesium sulfate and anhydrous sodium acetate is shown in Table 2. Conditions A and B assessed the effect of sample volume, B and C evaluated the influence of sodium acetate addition on phase separation, C and D examined the impact of MgSO_4_ addition on excess moisture absorption. There was no significant difference in extraction efficiency for multiple pesticide residues under the various extraction conditions described above. Therefore, the optimal extraction conditions for ferulic acid and ligustilide were selected based on the peak area of their mass spectrometry responses.

**Figure 1:**
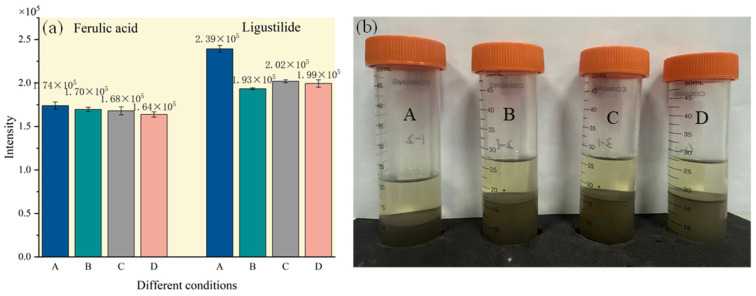
The intensity of ferulic acid and ligustilide and extract state in decoction under different conditions: (**a**) intensity; (**b**) extract state.

The peak areas were calculated based on a standardized sampling volume of 15 mL, which yielded the results depicted in Figure 1. Under condition A (10 mL decoction, 10 mL ACN, 4 g MgSO_4_, 1 g CH3COONa), both ferulic acid and ligustilide exhibited the highest peak areas in the extract (a), and ligustilide was particularly evident. Comparing the color and clarity of extracts obtained under the four different conditions, all extracts were found to be clear and transparent, though each exhibited a slight yellowish tint (b). Therefore, further purification to remove pigments and other impurities was required.

##### Purification

**Figure 2:**
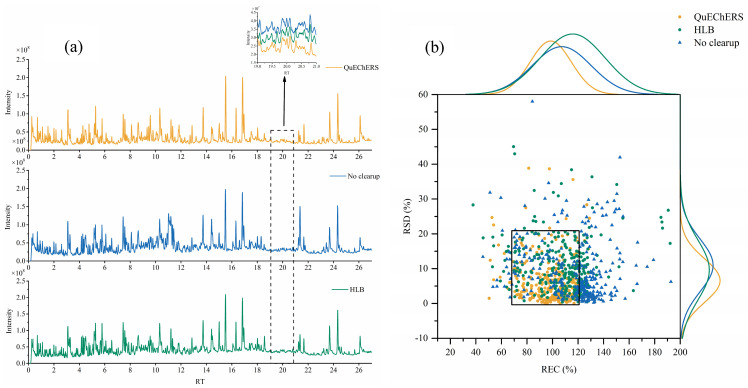
Purication effect of *AS* on different methods. (**a**) TIC of *AS* on different purication methods; (**b**) the pesticide recovery and RSD scatter plot. Note: The points enclosed in black boxes stand for pesticides spiked into AS, whose recovery rates range from 70 to 120% with RSD no more than 20%.

Both HLB and QuEChERS methods are suitable for the purification of samples with high water content. This experiment compared the purification effects of unpurified samples alongside QuEChERS and HLB purification methods on *AS* decoction. As the purification material adsorbs ferulic acid and ligustilide, these compounds were not involved in the purification step. The 270 pesticides were spiked at a concentration of 10 μg/L and analyzed after purification. Figure 2a presents the total ion chromatograms (TICs) for samples purified by different methods. Comparing the baselines revealed that the unpurified sample exhibited the highest baseline, followed by HLB, with QuEChERS showing the lowest. As the LC-Q-TOF/MS operated in full-scan mode, interfering ions in the sample directly contributed to the elevated baseline. Consequently, samples with fewer impurities yielded a lower baseline. It is concluded that the QuEChERS method achieved the most effective removal of impurities.

The scatter plot of pesticide recovery rates and RSDs, with the pesticide recovery rate on the *x*-axis and RSD on the *y*-axis, is shown in Figure 2b. Under QuEChERS, HLB, and unpurified conditions, the numbers of spiked pesticides in *AS* achieving recovery rates between 70–120% and RSD ≤ 20% were 274 (91%), 177 (59%) and 102 (34%), respectively. Above this scatter plot lies a normal distribution curve for recovery rates. The most concentrated distribution range was observed for samples purified using QuEChERS, followed by HLB, while the distribution range for unpurified samples exhibited the greatest dispersion. This outcome correlated with the degree of sample purification: the more thorough the purification, the greater the number of pesticides exhibiting recovery rates within the 70–120% range. Unpurified samples exhibited substantial interference ions, resulting in a broad recovery rate distribution with values skewed either high or low. The right-hand side of the scatter plot depicts the normal distribution of RSD. Samples purified using the QuEChERS exhibited the most concentrated RSD distribution, with the lowest values on the *Y*-axis, indicating superior consistency in sample purification. In summary, comparative analysis of recovery rates and RSDs across different purification processes demonstrated that QuEChERS extraction yielded superior pesticide recovery efficiency compared with HLB and unpurified samples.

### 3.2. Result of Angelica sinensis Pieces

**Figure 3:**
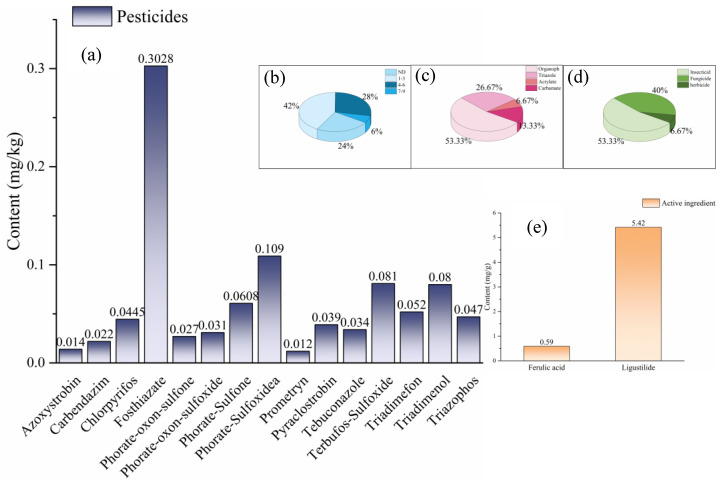
The non-targeted screening results of pesticides and Q-markers in *AS* pieces. (**a**) The average concentrations of pesticides in 50 samples; (**b**) the numbers of pesticides detected in each sample; (**c**) the chemical classifications of pesticides detected; (**d**) the functions of pesticides detected; (**e**) the average concentrations of Q-markers in 50 samples.

A non-targeted screening for pesticide residues and Q-marker was conducted on 50 *AS* samples collected from Gansu Province. Among these, 15 pesticides were detected in 38 samples (76%), with their average concentrations shown in Figure 3a. The numbers of pesticides detected in each sample are presented in Figure 3b, in which samples that detected 1–3 pesticides are prevalent. Their chemical classifications are exhibited in Figure 3c, indicating that organophosphorus pesticides were the most popular. Their functions are shown in Figure 3d, with insecticides being the most widely used. The average concentrations of the Q-markers ferulic acid and ligustilide are depicted in Figure 3e.

Among the 15 pesticides detected in *AS*, the *Chinese Pharmacopoeia* has established maximum residue limits (MRLs) only for phorate and terbufos. No maximum residue limits have been specified for the remaining pesticides detected. Referencing the *European Pharmacopoeia*’s MRL for chlorpyrifos in TCM (0.2 mg/kg) [33], one batch of *AS* samples exceeded this limit. Consequently, there is an urgent need to establish and refine relevant standards in China to enhance the quality of TCM while avoiding potential trade barriers in export markets.

### 3.3. Pesticide Transfer Behavior During Decoction

The pesticide spraying concentration of positive samples (Table 3) was set at 2000 times the actual detected concentration in *AS*. This was because the detected pesticide residues in raw herbs were generally at low levels. In addition, *AS* are soaked and decocted with a large amount of water according to Section 2.3.2, which greatly dilutes the pesticide concentration in the samples. This study systematically monitored the variations in the dissolved amounts of 15 pesticides during soaking, first decoction and secondary decoction, with statistical analysis performed via one-way ANOVA and Tukey’s post hoc test.

**Figure 4:**
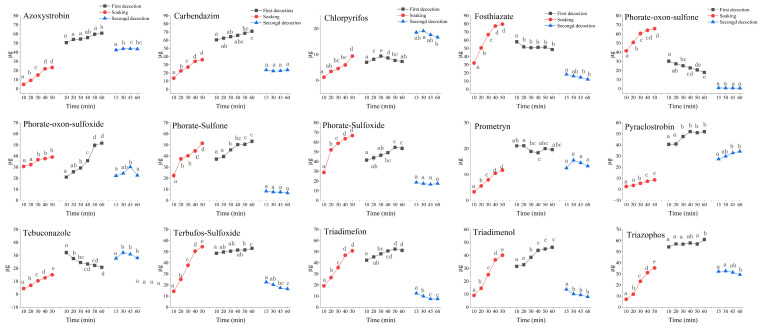
The dissolved masses of pesticide detected during the process of decoction in *AS*. Note: different letters indicate significant differences between groups (*p* < 0.05, Tukey’s test), while the same letter indicates no significant difference (*p* ≥ 0.05).

Samples were collected and tested at 10, 20, 30, 40, and 50 min of soaking, as shown in Figure 4 (red curve). Most pesticides showed a rapid increase in transfer amount within the first 40 min of soaking (*p* < 0.05). Approximately half of the compounds reached or approached the plateau phase within 40 min, including azoxystrobin, carbendazim, fosthiazate, phorate-sulfoxide, prometryn and triadimefon. This indicated that these pesticides were weakly bound to the sample matrix and easily eluted by the aqueous phase, thus being transferred into the decoction after short-term soaking. In contrast, the other half exhibited a continuous upward trend, such as chlorpyrifos, phorate-sulfone, tebuconazole, terbufos-sulfoxide, triadimenol and triazophos, suggesting that they bound more tightly to the matrix or underwent a slow desorption process (*p* < 0.05).

Samples were collected at 10, 20, 30, 40, 50, and 60 min into the first decoction phase (Figure 4 black curve). Throughout the decoction process, both pesticide evaporation through heating, thermal degradation and solid–liquid partitioning occurred concurrently. The combined effect of these factors resulted in a complex multifactorial variation in pesticide transfer into the decoction solution. As the first boiling time went on, the mass of pesticides dissolved in the decoction exhibited three primary trends: continuous increase, continuous decrease, or an initial increase followed by a decline. For certain water-soluble pesticides, such as carbendazim (WS: 2441 mg/L), phorate-sulfone (WS: 971.5 mg/L), and phorate-sulfoxide (WS: 507.5 mg/L), the dissolution mass increased during boiling (*p* < 0.05). This may be attributed to heating, which accelerates dissolution. Conversely, the dissolution of certain pesticides, including fosthiazate, phorate-oxon-sulfone, and tebuconazole, exhibited a decreasing trend (*p* < 0.05), which was likely due to loss or degradation during heating. The concentration of the pesticide chlorpyrifos initially increased before decreasing. The concentration peaked 30 min after decoction and subsequently declined gradually (*p* < 0.05).

Samples were collected for analysis at 15, 30, 45, and 60 min during the secondary boiling phase. The dissolution and transfer amounts of the 15 pesticide residues were generally at lower levels (blue curve) compared with the first decoction, aligning with the conclusions of a previous study [33]. For most pesticides, their dissolved contents increased slowly with the extension of secondary decoction time, and no significant statistical differences were observed among different time points (*p* ≥ 0.05), indicating that free and easily soluble pesticides in *AS* were almost completely released during primary decoction. The dissolved contents of several pesticides even decreased slightly continuously, which was attributed to volatilization and thermal degradation of pesticides under prolonged heating. A slight and slow increasing trend was only found for pyraclostrobin (*p* ≥ 0.05) during secondary decoction. Owing to its extremely low water solubility (0.08069 mg/L) and strong binding force with the *AS* matrix, prolonged secondary heating could further release part of deeply bound pesticide residues.

### 3.4. Pesticide Dissipation Kinetics Model in Piece

**Figure 5:**
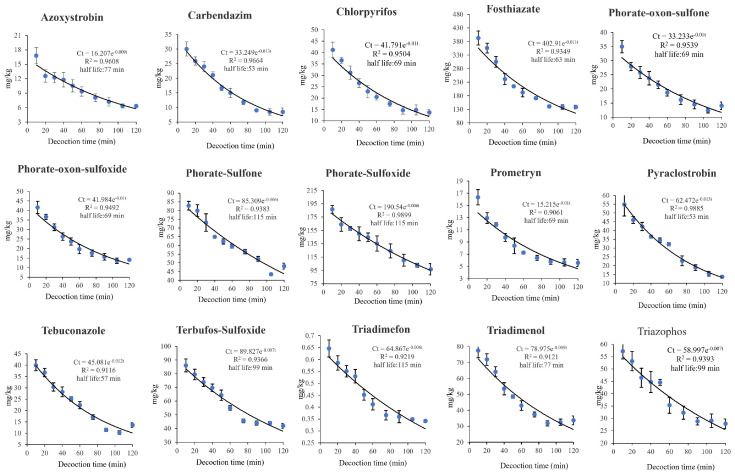
The dissipation curves and half–lives of pesticides in boiling.

Since the experimental conditions for the first and second decoctions of *AS* roots were identical, the total decoction time (first decoction: 60 min + second decoction: 60 min) was used as the time gradient to systematically determine the residual changes of 15 pesticides in the *AS* matrix. The dissipation kinetics of 15 representative pesticides during the simulated decoction were investigated (Figure 5). The concentrations of all target compounds decreased significantly with increasing decoction time, and the dissipation process conformed to the first-order kinetic model Ct = C_0_^−kt^, with all coefficients of determination (R^2^) greater than 0.91, indicating good model fitting that accurately described the dissipation patterns of the analytes.

Significant differences in dissipation rates were observed among different compounds, with half-lives (t_1/2_) ranging from 53 to 115 min. Among them, carbendazim and pyraclostrobin exhibited the fastest dissipation rates, both with a half-life of 53 min, while phorate-sulfone, phorate-sulfoxide, and triadimefon showed the slowest dissipation rates, with half-lives reaching 115 min. Further analysis revealed no significant correlation between the half-lives of pesticides and their physicochemical properties. The dissipation rates of pesticides during decoction are influenced by multiple factors rather than a simple transfer (dissolution) from the matrix to the decoction phase. It is a comprehensive result of simultaneous chemical degradation, evaporation loss, and repeated partitioning of pesticides between the matrix and the decoction phase, which increases the complexity of residue change.

### 3.5. The Thermal Degradation Mechanism of Chlorpyrifos

**Figure 6:**
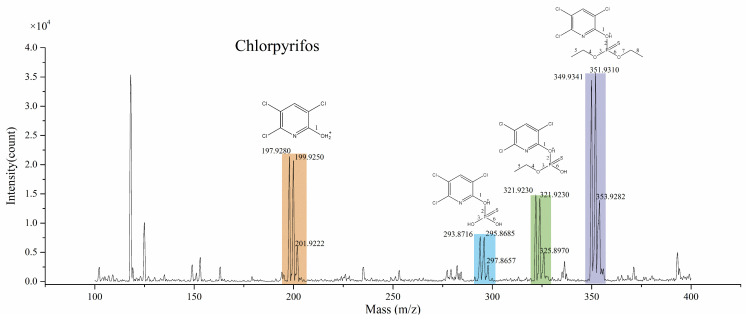
The degradation products of chlorpyrifos by LC-Q-TOF/MS in full-scan model.

**Figure 7:**
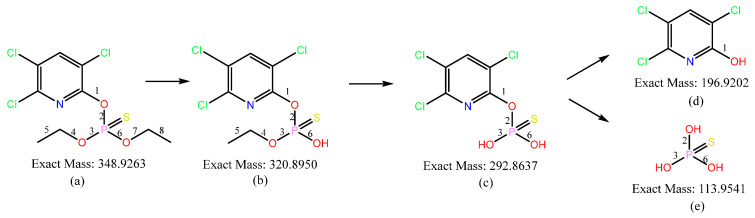
The molecular structural formula and degradation pathway of chlorpyrifos. (**a**) chlorpyrifos; (**b**) desethyl-chlorpyrifos; (**c**) 3,5,6-trichloro-2-pyridin-phosphorothioate; (**d**) 3,5,6-trichloro-2-pyridinol; (**e**) phosphorothioic acid.

During the decoction process of *AS*, short-term heating can accelerate the dissolution of chlorpyrifos, while prolonged heating promotes its degradation, leading to a reduction in concentration. To further investigate the thermal degradation mechanism of chlorpyrifos, full-scan monitoring data of primary mass spectrometry were collected using LC-Q-TOF/MS. Chlorpyrifos contains three chlorine atoms with an isotopic abundance ratio of 27:27:9:1. As shown in Figure 6, four isotopic peak clusters for chlorine were observed, with the isotope peak exhibiting a 27:27:9 abundance ratio being distinctly discernible. Based on its precise mass, the molecular structural formula and degradation pathway can be inferred, as depicted in Figure 7. Chlorpyrifos (a) first loses one ethyl group to form desethyl-chlorpyrifos (b), and then loses another ethyl group to form 3,5,6-trichloro-2-pyridine-phosphorothioate (c). Subsequently, the C-OP bond between the pyridine ring and the phosphorothioic acid in chlorpyrifos is broken through nucleophilic attack, resulting in the formation of 3,5,6-trichloro-2-pyridinol (d) and phosphorothioic acid (e).

**Figure 8:**
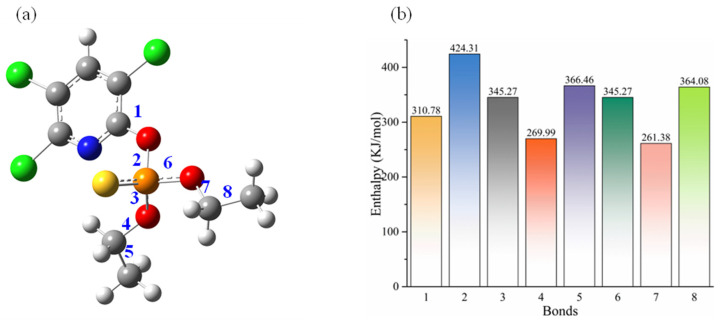
The optimized structure and dissociation energies of different chemical bonds of chlorpyrifos by DFT: (**a**) the optimized structure of chlorpyrifos; (**b**) the BDE of different chemical bonds of chlorpyrifos.

The BDE of chlorpyrifos was calculated using DFT to validate the aforementioned degradation pathways. To measure the difficulty of cleavage of different linkages in the backbones of chlorpyrifos, Gaussian09 software was used to calculate the BDE of different backbone linkages in the pyrolysis of chlorpyrifos. The optimized molecular configurations of chlorpyrifos are shown in Figure 8a. All models involved in the calculation process were confirmed by structural optimization and frequency calculations, and the frequency calculation results did not include virtual frequency. The BDE of different backbone linkages was the difference between the sum of the energy of all products after the linkages were broken and the energy of the reactants with stable molecular configurations [34]. The BDE of different chemical bonds is shown in Figure 8b. Bond 7 exhibited the weakest BDE (261.38 KJ/mol), making the ethyl group most readily eliminable to form desethyl-chlorpyrifos (b). Bond 4 followed with the second weakest BDE (269.99 KJ/mol), allowing elimination of another ethyl group to yield 3,5,6-trichloro-2-pyridin-phosphorothioate (c). Bond 1 possessed the third weakest BDE (310.78 KJ/mol), The carbon–oxygen bond formed between the pyridine ring and the phosphorothioic acid underwent cleavage via a nucleophilic attack, yielding 3,5,6-trichloro-2-pyridinol (d) and phosphorothioic acid (e). The thermal degradation pathway of chlorpyrifos was proposed through the identification of degradation products by LC-Q-TOF/MS. This proposed pathway was verified by a BDE calculation of the DFT analysis. This result was consistent with the findings of previous studies [35,36].

Based on the previous laboratory experiments and the ECOSAR model, the parent compound chlorpyrifos exhibits very high acute and chronic toxicity to fish, daphnia, and green algae. Among them, trichloropyridinol derivatives (products b, c, and d) still maintain extremely high aquatic toxicity. Some oxidation products are even more toxic than the parent chlorpyrifos, while only partial ring-opening degradation products are reduced to a harmless toxicity level [35,37,38].

### 3.6. Q-Markers Transfer Behavior During Decoction

**Figure 9:**
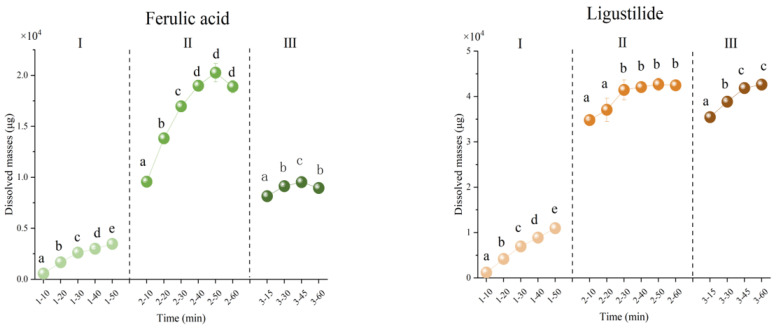
The dissolved masses of two Q-markers during the process of decoction in *AS*. (I) Soaking phase; (II) first decoction phase; (III) second decoction phase. Note: different letters indicate significant differences between groups (*p* < 0.05, Tukey’s test), while the same letter indicates no significant difference (*p* ≥ 0.05).

During the soaking of *AS*, the dissolution quantities of the Q-markers ferulic acid and ligustilide both exhibited a sustained increasing trend (*p* < 0.05). Ferulic acid reached its peak dissolution quantity at 50 min in the first boiling (*p* < 0.05), after which its dissolution quantity declined. Ligustilide peaked at 30 min (*p* < 0.05), maintaining a stable dissolution quantity thereafter (*p* ≥ 0.05). Both ferulic acid and ligustilide reached peak extraction levels after 45 min of the second boiling (*p* < 0.05). Ferulic acid extraction declined thereafter, while ligustilide extraction remained stable. Comparative analysis indicated that ligustilide exhibited greater physicochemical stability than ferulic acid. Taking into account the quality changes of pesticides and Q-markers in *AS*, soaking for 50 min, first boiling for 50 min, and second boiling for 45 min resulted in the highest amount of Q-markers (Figure 9).

### 3.7. Rransfer Rate of Pesticides and Q-Markers

Following the provisions of the ‘Regulations for the Management of Traditional Chinese Medicine Decoction Rooms in Medical Institutions’, decoction experiments were conducted to investigate the transfer rates of 15 pesticides and two Q-marker during the decoction process. Pesticide and Q-marker concentrations were measured in pieces before and after decoction according to Section 2.4.1, as well as in the decoction liquid according to Section 2.4.2. The transfer rates were calculated with Equation (1).

**Figure 10:**
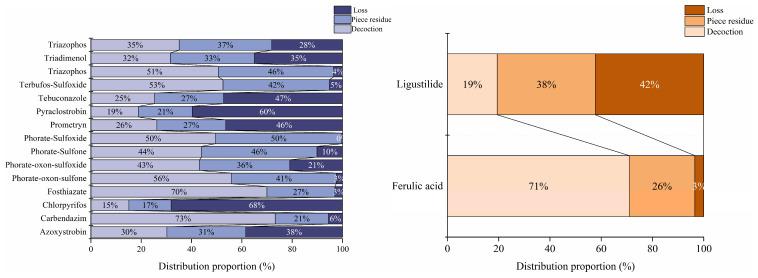
Distribution proportion of pesticides and Q-markers during the decoction process.

The distribution profiles of 15 target pesticides during the decoction process are presented in Figure 10, showing the proportions of pesticides retained in herbal pieces, transferred into the decoction, and lost during the process. After 50 min of soaking followed by 120 min of decoction, most pesticides exhibited a relatively balanced distribution between the decoction and the herbal residue, indicating that pesticide diffusion and partitioning reached a state of equilibrium. Therefore, the distribution proportion of pesticides retained in the herbal pieces is roughly equivalent to that transferred into the decoction liquid.

Significant variations in transfer rates were observed among different pesticides. Carbendazim, which is highly water-soluble, showed the highest transfer rate into the decoction (73%), whereas chlorpyrifos, a highly lipophilic compound, exhibited the lowest transfer rate (15%) and the highest proportion of loss (68%), likely due to volatilization or thermal degradation under the decoction conditions described in Section 3.5. For phorate-related pesticides, including phorate-sulfoxide, phorate-sulfone, phorate-oxon-sulfoxide, and phorate-oxon-sulfone, similar distribution characteristics were observed, with transfer rates ranging from 43% to 56% and the majority of the remaining pesticide being retained in the herbal residue. Other pesticides, such as tebuconazole, pyraclostrobin, and prometryn, showed moderate to high loss rates (46–60%), suggesting poor thermal stability during decoction.

**Figure 11:**
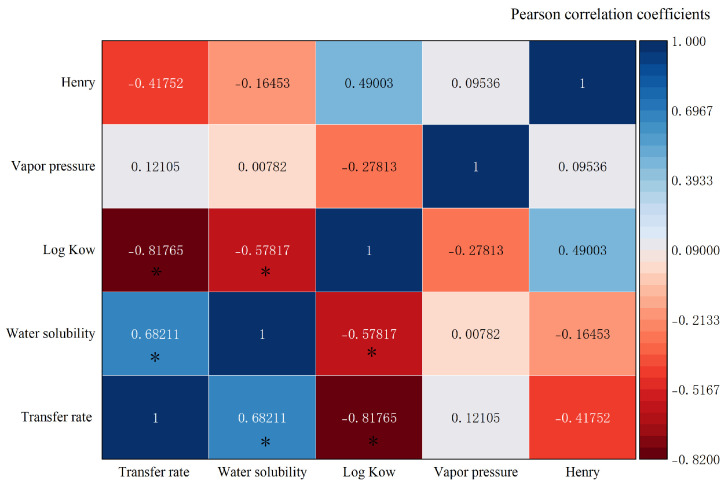
Pearson correlation coefficient heatmap of their transfer rate based on physicochemical properties. Note: * indicates statistically significant correlation at *p* < 0.05.

To further explore the key factors governing pesticide transfer behavior, Pearson correlation coefficients were calculated after Z-score standardization of the physicochemical parameters. As shown in the heatmap (Figure 11), the transfer rate exhibited a strong positive correlation with WS (r = 0.68211, *p* < 0.05) and a strong negative correlation with log K_ow_ (r = −0.81765, *p* < 0.05). In comparison, only a moderate negative correlation was observed with Henry constant (r = −0.41752), and a weak positive correlation with vapor pressure (r = 0.12105). These results indicate that WS and log K_ow_ are the primary factors determining pesticide partitioning between the herbal matrix and the aqueous phase during decoction. Compounds with higher WS and lower log K_ow_ tend to transfer more readily into the decoction, while lipophilic pesticides are more likely to remain in the herbal residue or undergo volatilization/degradation under heating conditions. These findings are consistent with previous studies on pesticide behavior during the decoction of TCM, further confirming the reliability of the observed distribution patterns [39,40].

**Figure 12:**
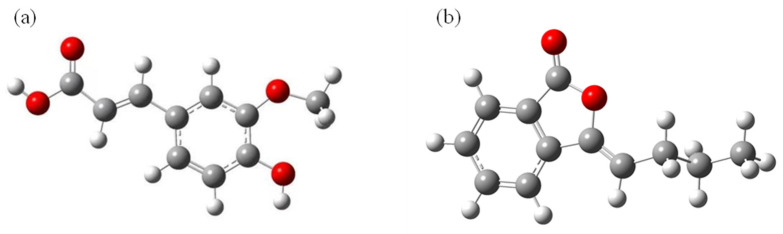
The optimised molecular configurations of ferulic acid and ligustilide by DFT. (**a**) Ferulic acid; (**b**) ligustilide.

The transfer rate of ferulic acid was 71%, while that of ligustilide was 19%, indicating a significant disparity in transfer efficiency between these two Q-markers. This discrepancy arises from their differing solubility properties: ferulic acid is water-soluble, and ligustilide is fat-soluble. Compounds with higher water solubility exhibit greater transfer rates into the decoction. The thermal loss of ferulic acid was 3%, while that of ligustilide was 42%, reflecting the compounds’ differing thermal stability (Figure 10). Theoretical calculations were performed using Gaussian 09 software, employing DFT with the B3LYP/6-31G(d) basis set for structural optimisation and frequency calculations. The optimised molecular configurations of ferulic acid and ligustilide are shown in Figure 12. The high-precision thermodynamic values were calibrated using the Shermo program. The electronic energy, internal energy, enthalpy, and Gibbs free energy are compared in Table 4. The thermodynamic values of ferulic acid were all significantly lower than those of ligustilide, indicating greater thermodynamic stability. This theoretically explains the higher thermal loss observed for ligustilide.

### 3.8. Risk Assessment Results

The assessment of chronic dietary intake risks for adults and children, alongside acute dietary intake risks calculated with Equations (4)–(7), is presented in Table 5. The dietary intake risks of terbufos-class, phorate-class, triazophos, and fosthiazatein pesticides exhibited elevated dietary intake risks in *AS*. The adult HQc ranged from 3.17 × 10^−6^ to 1.16 × 10^−2^, while the pediatric HQc ranged from 1.27 × 10^−5^ to 4.63 × 10^−2^. Since all HQc values were below 1, the pesticide residues in *AS* were within acceptable levels, with no anticipated health concerns from long-term consumption. The adult HQa ranged from 1.15 × 10^−5^ to 4.22 × 10^−2^, and the pediatric HQa ranged from 4.59 × 10^−5^ to 0.169. Although all HQa values were below 1, the chronic dietary intake risk for *AS* in children reached 0.169, warranting attention.

From the perspective of toxicological contribution, organophosphorus pesticides and their metabolites, such as phorate sulfoxide and phorate sulfone, act as the main contributors to chronic and acute dietary risks in children owing to their extremely low ADI and ARfD values. In contrast, highly water-soluble pesticides including carbendazim present high transfer rates but relatively low toxicity, thus failing to yield the highest hazard quotient. This finding reveals that the dietary risk of pesticide residues is not merely determined by the residual concentration in raw medicinal materials but results from the combined effects of residue level, transfer rate, toxicological threshold and body weight of exposed populations.

Although the transfer rate has been incorporated into the risk assessment formula, certain limitations still remain. Firstly, the decoction process is oversimplified in the evaluation system, which tends to cause considerable deviations between assessed results and actual exposure risks. Secondly, the current dietary intake risk assessment method only calculates pesticide intake from single medicinal herbs. In practical application, traditional Chinese medicines are commonly decocted in compound formulas consisting of multiple herbs. Consequently, the cumulative pesticide intake from combined herbal prescriptions may give rise to unacceptable dietary health risks.

## 4. Conclusions

Based on LC-Q-TOF/MS, a high-throughput analytical method was established for the simultaneous determination of 270 pesticides and two Q-Markers (ferulic acid and ligustilide) in *AS* decoction. Fifteen pesticides together with the two Q-Markers were detected in 50 batches of samples. Their dynamic concentration variations were monitored during 50 min of soaking, 60 min of first boiling and 60 min of second boiling processes. Distinct differences were observed in the transfer behaviors of the pesticides and Q-Markers across the three processing stages. It is a synergistic outcome of multiple processes, including solid–liquid partitioning, internal diffusion within the matrix, physical volatilization, and chemical degradation under high temperature conditions. These influencing factors interact with each other, resulting in complex kinetic characteristics of pesticide transfer and dissipation. Chlorpyrifos was verified to undergo chemical degradation under boiling conditions, and its degradation pathway was further confirmed by LC-Q-TOF/MS identification combined with DFT calculations. In addition, the decoction transfer rates of all detected analytes were calculated. The results showed that the transfer rates were significantly positively correlated with WS (r = 0.68211, *p* < 0.05) and markedly negatively correlated with log K_ow_ (r = −0.81765, *p* < 0.05). Obvious disparities were also found in the thermal loss degrees between ferulic acid and ligustilide, and DFT-based thermodynamic analysis elucidated the underlying mechanisms responsible for such thermal losses. Finally, a pesticide residue risk assessment model for SMFH was constructed based on the acquired transfer rate data. Risk assessment results demonstrated that the cumulative toxic risks of pesticide residues in *AS* decoction were generally within the acceptable safety range, with all HQa values lower than 1. Nevertheless, the chronic dietary intake risk for children was calculated to be 0.169, which deserves targeted attention. This study provides reliable methodological support and theoretical reference for the quality control, safety evaluation and processing optimization of SMFH.

Despite the valuable findings obtained in this study, several limitations should be acknowledged. The present investigation was specifically performed on a single *AS* decoction. Given that different TCM formulas contain diverse herbal ingredients with complex matrix environments, variations in herb–matrix interactions, component dissolution behaviors, and pesticide binding effects may lead to distinct transfer and degradation characteristics in compound prescriptions. Moreover, this study was under conventional household and standardized boiling protocols. The pesticide content change in the dynamic process was diversified due to different decoction technological parameters (e.g., boiling temperature, water addition amount and decoction duration). Lastly, the risk assessment model herein adopted partial rough estimations and omitted several potential critical influencing factors, which introduced unavoidable uncertainty into the final risk prediction results.

## Figures and Tables

**Table 1:** Accuracy and precision of *AS* decoction.

Content of Ferulic Acid (g/L)	Standard Added of Ferulic Acid (g/L)	Measured of Ferulic Acid (g/L)	Recovery of Ferulic Acid %	Ave Recover of Ferulic Acidy %	RSD of Ferulic Acid (%)	RSDwR of Ferulic Acid (%)	Content of Ligustilide (g/L)	Standard Added of Ligustilide (g/L)	Measured of Ligustilide (g/L)	Recovery of Ligustilide %	Ave Recovery of Ligustilide %	RSD of Ligustilide (%)	RSDwR of Ligustilide (%)
0.060	0.060	0.115	0.96	96.9	2.7	3.2	0.532	0.500	1.004	0.97	95.4	2.2	2.6
0.058	0.060	0.118	1.00				0.540	0.500	1.025	0.99			
0.058	0.060	0.112	0.95				0.531	0.500	0.984	0.95			
0.059	0.060	0.117	0.98				0.559	0.500	0.993	0.94			
0.059	0.060	0.111	0.93				0.542	0.500	0.972	0.93			
0.058	0.060	0.117	0.99				0.551	0.500	0.990	0.94			

**Table 2:** Different extraction conditions.

Conditions	Decoction (mL)	Acetonitrile (1% Acetic Acid) (mL)	MgSO_4_ (g)	CH_3_COONa (g)
A	10	10	4	1
B	15	10	6	1.5
C	15	10	6	2
D	15	10	8	2

**Table 3:** The concentration and type of pesticides in the fortified samples (n = 3, g/kg).

No.	Pesticides	Spraied Concentration	Detected Concentration (RSD)	Function	Type	Toxicity
1	Azoxystrobin	0.0280	0.0219 (4.34)	Fungicide	Acrylate	Slightly
2	Carbendazim	0.0440	0.0387 (2.54)	Fungicide	Carbamate	Slightly
3	Chlorpyrifos	0.0890	0.0776 (3.49)	Insecticide	Organophosphorus	Moderate
4	Fosthiazate	0.6056	0.5051 (4.37)	Insecticide	Organoph	Moderate
5	Phorate-oxon-sulfone	0.0540	0.0377 (4.34)	Insecticide	Organoph	Hypertoxic
6	Phorate-oxon-sulfoxide	0.0620	0.0439 (2.39)	Insecticide	Organoph	Hypertoxic
7	Phorate-Sulfone	0.1216	0.1067 (2.47)	Insecticide	Organoph	Hypertoxic
8	Phorate-Sulfoxidea	0.2180	0.2078 (4.94)	Insecticide	Organoph	Hypertoxic
9	Prometryn	0.0240	0.0205 (4.81)	Herbicide	Triazine	Slightly
10	Pyraclostrobin	0.0780	0.0625 (3.36)	Fungicide	Carbamate	Slightly
11	Tebuconazole	0.0680	0.0486 (4.65)	Fungicide	Triazole	Moderate
12	Terbufos-Sulfoxide	0.1620	0.1064 (2.13)	Insecticide	Organoph	Hypertoxic
13	Triadimefon	0.1040	0.0774 (3.34)	Fungicide	Triazole	Moderate
14	Triadimenol	0.1600	0.1005 (2.29)	Fungicide	Triazole	Moderate
15	Triazophos	0.0940	0.0746 (2.89)	Insecticide	Organoph	Highly

**Table 4:** The comparison of thermodynamic values between ferulic acid and ligustilide.

Compounds	Electronic Energy (Har)	Internal Energy (Har)	Enthalpy (Har)	Gibbs Free Energy (Har)
Ferulic acid	−688.120344	−687.9294882	−687.9285441	−687.9802962
Ligustilide	−615.101951	−614.8855314	−614.8845872	−614.9359870

**Table 5:** Risk assessment of acute and chronic dietary intake of *AS*.

Pesticides	Transfer Rate (%)	ADI (mg/kg)	Risk Assessment of Chronic Dietary Intake				ARfD (mg/kg)	Acute Dietary Intake Risk Assessment			
			EDI (mg/kg BW)		HQc			ESTI (mg/kg BW)		HQa	
			Adult	Child	Adult	Child		Adult	Child	Adult	Child
Azoxystrobin ^a^	30.21	0.2	6.34 × 10^−7^	2.54 × 10^−6^	3.17 × 10^−6^	1.27 × 10^−5^	Unnecessary	1.15 × 10^−6^	4.59 × 10^−6^		
Carbendazim ^a^	73.26	0.03	2.42 × 10^−6^	9.67 × 10^−6^	8.06 × 10^−5^	3.22 × 10^−4^	0.1	8.94 × 10^−6^	3.58 × 10^−5^	8.94 × 10^−5^	3.58 × 10^−4^
Chlorpyrifos ^a^	15.09	0.01	1.01 × 10^−6^	4.03 × 10^−6^	1.01 × 10^−4^	4.03 × 10^−4^	0.1	9.71 × 10^−6^	3.89 × 10^−5^	9.71 × 10^−5^	3.89 × 10^−4^
Fosthiazate ^a^	69.97	0.004	3.18 × 10^−5^	1.27 × 10^−4^	7.95 × 10^−3^	3.18 × 10^−2^	-	7.70 × 10^−5^	3.08 × 10^−4^		
Phorate-oxon-sulfone ^b^	55.97	0.0007	2.27 × 10^−6^	9.07 × 10^−6^	3.24 × 10^−3^	1.30 × 10^−2^	0.003	1.46 × 10^−5^	5.82 × 10^−5^	4.85 × 10^−3^	1.94 × 10^−2^
Phorate-oxon-sulfoxide ^b^	43.2	0.0007	2.01 × 10^−6^	8.04 × 10^−6^	2.87 × 10^−3^	1.15 × 10^−2^	0.003	2.68 × 10^−6^	1.07 × 10^−5^	8.93 × 10^−4^	3.57 × 10^−3^
Phorate-Sulfone ^b^	43.95	0.0007	4.01 × 10^−6^	1.60 × 10^−5^	5.73 × 10^−3^	2.29 × 10^−2^	0.003	1.94 × 10^−5^	7.75 × 10^−5^	6.46 × 10^−3^	2.58 × 10^−2^
Phorate-Sulfoxidea ^b^	49.54	0.0007	8.10 × 10^−6^	3.24 × 10^−5^	1.16 × 10^−2^	4.63 × 10^−2^	0.003	1.27 × 10^−4^	5.07 × 10^−4^	4.22 × 10^−2^	1.69 × 10^−1^
Prometryn ^a^	26.21	0.04	4.72 × 10^−7^	1.89 × 10^−6^	1.18 × 10^−5^	4.72 × 10^−5^	-	7.34 × 10^−7^	2.94 × 10^−6^		
Pyraclostrobin ^a^	18.91	0.03	1.11 × 10^−6^	4.42 × 10^−6^	3.69 × 10^−5^	1.47 × 10^−4^	0.05	1.47 × 10^−6^	5.90 × 10^−6^	2.95 × 10^−5^	1.18 × 10^−4^
Tebuconazole ^a^	25.3	0.03	1.29 × 10^−6^	5.16 × 10^−6^	4.30 × 10^−5^	1.72 × 10^−4^	0.3	3.44 × 10^−6^	1.38 × 10^−5^	1.15 × 10^−5^	4.59 × 10^−5^
Terbufos-Sulfoxide ^c^	52.5	0.0006	6.38 × 10^−6^	2.55 × 10^−5^	1.06 × 10^−2^	4.25 × 10^−2^	0.002	8.51 × 10^−6^	3.40 × 10^−5^	4.25 × 10^−3^	1.70 × 10^−2^
Triadimefon ^a^	50.61	0.03	3.95 × 10^−6^	1.58 × 10^−5^	1.32 × 10^−4^	5.26 × 10^−4^	0.08	1.87 × 10^−5^	7.49 × 10^−5^	2.34 × 10^−4^	9.36 × 10^−4^
Triadimenol ^a^	31.55	0.03	3.79 × 10^−6^	1.51 × 10^−5^	1.26 × 10^−4^	5.05 × 10^−4^	0.08	9.68 × 10^−6^	3.87 × 10^−5^	1.21 × 10^−4^	4.84 × 10^−4^
Triazophos ^a^	35.06	0.001	2.47 × 10^−6^	9.89 × 10^−6^	2.47 × 10^−3^	9.89 × 10^−3^	0.001	3.30 × 10^−6^	1.32 × 10^−5^	3.30 × 10^−3^	1.32 × 10^−2^
HI					4.50 × 10^−2^	1.80 × 10^−1^				6.26 × 10^−2^	2.50 × 10^−1^

^a^ Estimated maximum residue limit (eMRL); ^b^ as a homologue of phorate, it was calculated according to it; ^c^ as a homologue of terbufos, it was calculated according to it.

## Data Availability

The original contributions presented in this study are included in the article. Further inquiries can be directed to the corresponding authors.

## Supplementary Materials

The following supporting information can be downloaded at: https://www.mdpi.com/article/10.3390/foods15122222/s1, Table S1: PCDL database on 270 pesticides and two Q-markers; Table S2: Methodological validation of 270 pesticides in *AS* decoction; Table S3: Methodological validation of 270 pesticides and two Q-markers in *AS* piece.

## Abbreviations

The following abbreviations are used in this manuscript:

ADI	Allowable Daily Intake
ARfD	Acute Reference Dose
*AS*	*Angelica sinensis*
BDE	Bond Dissociation Energy
DFT	Density Functional Theory
EDI	Estimated Daily Intake
ESTI	Estimated Short-Term Intake
EFSA	European Food Safety Authority
HI	Hazard Index
HQc	Chronic Hazard Quotient
HQa	Acute Hazard Quotient
JMPR	Joint Meeting on Pesticide Residues
LC-Q-TOF/MS	Liquid Chromatography Quadrupole Time-of-Flight Mass Spectrometry
LOQ	Limit of Quantification
MRL	Maximum Residue Limits
PCDL	Personal Compound Database and Library Manager
Q-markers	Quality Markers
RSD	Relative Standard Deviation
RSDwR	Within-laboratory Reproducibility
SMFH	Substances of Medicine Food Homology
TCM	Traditional Chinese Medicine
