# Supplementary material for: Simultaneous Determination, Transfer Behaviors, Degradation, and Risk Assessment of Pesticides and Q-Marker in Angelica sinensis During Decoction
Source: Foods. 2026 Jun 19;15(12):2222. doi: 10.3390/foods15122222 (PMC13298167; doi:10.3390/foods15122222)
Supplement: Supplementary file 1 [file foods-15-02222-s001.zip › foods-4304540-supplementary.pdf]

Table S1. PCDL database on 270 pesticides and two Q-markers

No.	Pesticides	Chemical formula	Retention Time (min)	Actual precursor ion(*m*/*z*)	Daughter ion(*m*/*z*)	Theoretical precursor ion(*m*/*z*)	Additive form	Mass deviation (ppm)
1	1-naphthyl acetamide	$C_{12}H_{11}NO$	4.456	186.0914	115.0542,141.0699,142.0733	186.0919	$[M+H]^+$	-2.69
2	2,6-Dichlorobenzamide	$C_7H_5Cl_2NO$	3.266	189.9821	172.9556,108.984,144.9606	189.9826	$[M+H]^+$	-2.63
3	3,4,5-Trimethacarb	$C_{11}H_{15}NO_2$	7.181	194.1173	137.0961,122.0726,109.1012	194.1181	$[M+H]^+$	-4.12
4	Acetamiprid-(E)	$C_{10}H_{11}ClN_4$	3.962	223.0745	126.0105,98.9996,90.0338,56.0495	223.075	$[M+H]^+$	-2.24
5	Acetamiprid-(Z)	$C_{10}H_{11}ClN_4$	3.961	223.0745	126.0105,98.9996,90.0338,56.0495	223.075	$[M+H]^+$	-2.24
6	Aldicarb	$C_7H_{14}N_2O_2S$	4.964	191.0849	116.0528,89.0419	191.0854	$[M+H]^+$	-2.62
7	Aldicarb-sulfone	$C_7H_{14}N_2O_4S$	2.625	223.0747	86.0600,59.0491,58.0287	223.0753	$[M+H]^+$	-2.69
8	Aldicarb-sulfoxide	$C_7H_{14}N_2O_3S$	2.261	207.0798	89.0419,58.0287,65.0056	207.0803	$[M+H]^+$	-2.41
9	Ametryn	$C_9H_{17}N_5S$	6.867	228.1277	68.0243,186.0808,71.0604	228.1283	$[M+H]^+$	-2.63
10	Aminocarb	$C_{11}H_{16}N_2O_2$	2.249	209.1285	137.0835,152.1070,122.0600	209.129	$[M+H]^+$	-2.39
11	Ancymidol	$C_{15}H_{16}N_2O_2$	5.332	257.1285	81.0447,135.0441,54.0338,107.0491, 121.0648	257.129	$[M+H]^+$	-1.94
12	Anilofos	$C_{13}H_{19}ClNO_3PS_2$	15.127	368.0299	124.9821,198.9647,170.9698	368.0311	$[M+H]^+$	-3.26
13	Aramite	$C_{15}H_{23}ClO_4S$	17.436	352.1347	191.1430,135.0804,111.0474	352.1349	$[M+NH4]^+$	-0.57
14	Atraton	$C_9H_{17}N_5O$	4.313	212.1506	68.0243,170.1036,100.0505	212.1511	$[M+H]^+$	-2.36
15	Atrazine-desisopropyl	$C_5H_8ClN_5$	2.937	174.0541	68.0243,96.0556,104.0010	174.0546	$[M+H]^+$	-2.87

No.	Pesticides	Chemical formula	Retention Time (min)	Actual precursor ion(*m*/*z*)	Daughter ion(*m*/*z*)	Theoretical precursor ion(*m*/*z*)	Additive form	Mass deviation (ppm)
16	Azaconazole	$C_{12}H_{11}Cl_2N_3O_2$	6.21	300.0301	158.9762,230.9974,172.9555,70.0399	300.0307	$[M+H]^+$	-2
17	azadirachtin	$C_{35}H_{44}O_{16}$	6.609	743.2517	665.2228,725.2407,625.1916	743.2527	$[M+Na]^+$	-1.35
18	Aziprotryne	$C_7H_{11}N_7S$	10.196	226.0869	68.0243,156.0338,125.0822,89.0168,58.0651	226.0875	$[M+H]^+$	-2.65
19	Azoxystrobin-(E)	$C_{22}H_{17}N_3O_5$	10.976	404.1241	329.0795,372.0979,344.1030	404.1246	$[M+H]^+$	-1.24
20	Azoxystrobin-(Z)	$C_{22}H_{17}N_3O_5$	9.277	404.1241	329.0795,372.0979,344.1030	404.1246	$[M+H]^+$	-1.24
21	Beflubutamid	$C_{18}H_{17}F_4NO_2$	14.679	356.1268	162.0913,177.1148,221.0584	356.1274	$[M+H]^+$	-1.68
22	Benalaxyl	$C_{20}H_{23}NO_3$	14.45	326.1751	91.0542,148.1121,208.1332,294.1489,121.0886	326.1756	$[M+H]^+$	-1.53
23	Bendiocarb	$C_{11}H_{13}NO_4$	5.98	224.0917	81.0335,109.0284,167.0703	224.0923	$[M+H]^+$	-2.68
24	Benodanil	$C_{13}H_{10}INO$	8.773	323.988	230.9301,202.9352,76.0308	323.9885	$[M+H]^+$	-1.54
25	Bensulide	$C_{14}H_{24}NO_4PS_3$	15.479	398.0678	313.9736,77.0386,158.0270	398.0683	$[M+H]^+$	-1.26
26	Benzovindiflupyr	$C_{18}H_{15}Cl_2F_2N_3O$	14.717	398.064	159.0364,342.0804,378.0571	398.0638	$[M+H]^+$	0.5
27	Bitertanol	$C_{20}H_{23}N_3O_2$	13.057	338.1863	70.0400,99.0804,269.1536	338.1869	$[M+H]^+$	-1.77
28	Blasticidin-S	$C_{17}H_{26}N_8O_5$	0.769	423.2104	74.0713,312.1666,124.0393,154.0975,81.0335	423.2104	$[M+H]^+$	0
29	Boscalid	$C_{18}H_{12}Cl_2N_2O$	11.702	343.0399	271.0866,307.0633,272.0944	343.0405	$[M+H]^+$	-1.75
30	Bromacil	$C_9H_{13}BrN_2O_2$	4.969	261.0233	131.9443,204.9607,54.0338	261.0239	$[M+H]^+$	-2.3
31	Bromfenvinfos-Methyl	$C_{10}H_{10}BrCl_2O_4P$	11.461	374.895	127.0155,169.9685,109.0051	374.8955	$[M+H]^+$	-1.33
32	Bromobutide	$C_{15}H_{22}BrNO$	14.174	312.0958	119.0855,194.0175,139.9529	312.0963	$[M+H]^+$	-1.6
33	Bromuconazole	$C_{13}H_{12}BrCl_2N_3O$	10.857	375.9514	158.9762,70.0399,172.9555,227.0006	375.9519	$[M+H]^+$	-1.33
34	Bupirimate	$C_{13}H_{24}N_4O_3S$	13.006	317.1642	166.0975,108.0114,210.1601	317.1647	$[M+H]^+$	-1.58
35	Butafenacil	$C_{20}H_{18}ClF_3N_2$	14.543	492.1157	331.0092,349.0197,333.0067	492.1149	$[M+NH4]^+$	1.63

No.	Pesticides	Chemical formula	Retention Time (min)	Actual precursor ion(*m/z*)	Daughter ion(*m/z*)	Theoretical precursor ion(*m/z*)	Additive form	Mass deviation (ppm)
		O_6						
36	Butamifos	$C_{13}H_{21}N_2O_4PS$	16.74	333.1035	95.9668,152.0293,180.0607,260.0141,231.9828	333.1038	$[M+H]^+$	-0.9
37	Cadusafos	$C_{10}H_{23}O_2PS_2$	14.62	271.095	96.9508,130.9385,158.9698	271.0955	$[M+H]^+$	-1.84
38	Cafenstrole	$C_{16}H_{22}N_4O_3S$	13.21	351.1485	72.0444,100.0757,101.0787	351.1491	$[M+H]^+$	-1.71
39	Carbendazim	$C_9H_9N_3O_2$	2.665	192.0768	160.0505,132.0556,105.0447	192.0773	$[M+H]^+$	-2.6
40	Carbofuran	$C_{12}H_{15}NO_3$	5.793	222.1125	123.0441,165.0910,77.0386	222.113	$[M+H]^+$	-2.25
41	Carbofuran-3-Hydroxy	$C_{12}H_{15}NO_4$	3.547	238.1074	107.0491,163.0754,135.0804	238.1079	$[M+H]^+$	-2.1
42	Carfentrazone-ethyl	$C_{15}H_{14}Cl_2F_3N_3O_3$	14.593	412.0435	345.9956,347.9929,302.0302	412.0443	$[M+H]^+$	-1.94
43	Carpropamid	$C_{15}H_{18}Cl_3NO$	14.973	334.0527	139.0309,196.0291,198.0262	334.0532	$[M+H]^+$	-1.5
44	Chlordimeform	$C_{10}H_{13}ClN_2$	3.366	197.084	117.0573,152.0262,125.0153	197.0846	$[M+H]^+$	-3.04
45	Chlorfenvinphos-(E)	$C_{12}H_{14}Cl_3O_4P$	14.251	358.9768	204.9373,169.9685,155.0468,127.0155,98.9842,80.9736	358.9774	$[M+H]^+$	-1.67
46	Chlorfenvinphos-(Z)	$C_{12}H_{14}Cl_3O_4P$	13.551	358.9768	204.9373,169.9685,155.0468,127.0155,98.9842,80.9736	358.9774	$[M+H]^+$	-1.67
47	Chloridazon	$C_{10}H_8ClN_3O$	3.747	222.0429	77.0386,65.0386,92.0495	222.0434	$[M+H]^+$	-2.25
48	Chlorimuron-ethyl	$C_{15}H_{15}ClN_4O_6S$	11.168	415.0474	186.0060,83.0240,121.0284,184.9895,143.0002	415.0479	$[M+H]^+$	-1.2
49	Chlorpyrifos	$C_9H_{11}Cl_3NO_3PS$	17.739	349.9336	96.9508,197.9268,321.9023	349.9341	$[M+H]^+$	-1.43
50	Chlorsulfuron	$C_{12}H_{12}ClN_5O_4$	6.187	358.0371	56.0495,141.0771,167.0564	358.0377	$[M+H]^+$	-1.68

No.	Pesticides	Chemical formula	Retention Time (min)	Actual precursor ion(*m*/*z*)	Daughter ion(*m*/*z*)	Theoretical precursor ion(*m*/*z*)	Additive form	Mass deviation (ppm)
		S						
51	Clodinafop-propargyl	$C_{17}H_{13}ClFNO_4$	15.42	350.0589	91.0542,266.0367,222.0105	350.0595	$[M+H]^+$	-1.71
52	Clomazone	$C_{12}H_{14}ClNO_2$	8.299	240.0786	125.0153,89.0386,98.9996	240.0791	$[M+H]^+$	-2.08
53	Cloransulam-methyl	$C_{15}H_{13}ClFN_5O_5S$	8.043	430.0377	369.9807,398.0121,152.9976,400.0094,371.9781	430.0388	$[M+H]^+$	-2.56
54	Crufomate	$C_{12}H_{19}ClNO_3P$	11.114	292.0864	108.0209,236.0638,238.0211	292.0869	$[M+H]^+$	-1.71
55	Cyanazine	$C_9H_{13}ClN_6$	5.364	241.0963	214.0854,68.0243,71.0604	241.0968	$[M+H]^+$	-2.07
56	Cyazofamid	$C_{13}H_{13}ClN_4O_2S$	14.582	325.0521	108.0114,216.0323,190.0292	325.0526	$[M+H]^+$	-1.54
57	Cycloate	$C_{11}H_{21}NOS$	15.349	216.1417	55.0542,83.0855,63.0263,154.1226,72.0444	216.1422	$[M+H]^+$	-2.31
58	Cycluron	$C_{11}H_{22}N_2O$	6.594	199.1805	69.0699,89.0709,55.0542	199.181	$[M+H]^+$	-2.51
59	Cyenopyrafen	$C_{24}H_{31}N_3O_2$	18.772	394.2489	310.1914,254.1301,57.0699	394.2495	$[M+H]^+$	-1.52
60	Cyflufenamid	$C_{20}H_{17}F_5N_2O_2$	16.813	413.1283	203.0227,241.0395,295.0864	413.1288	$[M+H]^+$	-1.21
61	Cymiazole	$C_{12}H_{14}N_2S$	3.846	219.095	77.0386,144.0808,130.0651	219.0956	$[M+H]^+$	-2.74
62	Cymoxanil	$C_7H_{10}N_4O_3$	4.179	199.0826	53.0134,111.0189,128.0455,83.0240,58.0287	199.0831	$[M+H]^+$	-2.51
63	Cyprazine	$C_9H_{14}ClN_5$	6.364	228.1007	186.0541,108.0556,68.0243	228.1016	$[M+H]^+$	-3.95
64	Cyproconazole	$C_{15}H_{18}ClN_3O$	9.474	292.1211	138.9945,125.0153,70.0400	292.1217	$[M+H]^+$	-2.05
65	Cyprofuram	$C_{14}H_{14}ClNO_3$	7.125	280.0735	69.0340,131.0730,184.0519	280.074	$[M+H]^+$	-1.79
66	Daimuron	$C_{17}H_{20}N_2O$	11.635	269.1656	91.0542,119.0855,151.0866	269.1654	$[M+H]^+$	0.74
67	demeton	$C_8H_{19}O_3PS_2$	7.805	259.0586	61.0106,55.0542,89.0419	259.0591	$[M+H]^+$	-1.93

No.	Pesticides	Chemical formula	Retention Time (min)	Actual precursor ion(*m/z*)	Daughter ion(*m/z*)	Theoretical precursor ion(*m/z*)	Additive form	Mass deviation (ppm)
68	Desethylterbuthylazine	$C_7H_{12}ClN_5$	5.183	201.0781	188.0513,145.0150,83.0604,104.0010,110.0461	201.0781	$[M+H]^+$	0
69	Desmetryn	$C_8H_{15}N_5S$	5.309	214.1121	172.0651,82.0400,57.0447	214.1126	$[M+H]^+$	-2.34
70	Diazinon	$C_{12}H_{21}N_2O_3PS$	15.371	305.1083	96.9508,169.0794,153.1022,249.0454,277.077	305.1089	$[M+H]^+$	-1.97
71	Dichlormid	$C_8H_{11}Cl_2NO$	6.707	208.029	84.0808,100.1121,98.0964,86.0964,58.0651	208.0296	$[M+H]^+$	-2.88
72	Diclocymet	$C_{15}H_{18}Cl_2N_2O$	13.362	313.0869	172.9919,174.9890,114.0913	313.0874	$[M+H]^+$	-1.6
73	Diclosulam	$C_{13}H_{10}Cl_2FN_5O_3S$	8.575	405.9938	160.9794,377.9625,223.9327,314.0006,175.9658	405.9944	$[M+H]^+$	-1.48
74	Diethyltoluamide	$C_{12}H_{17}NO$	6.872	192.1383	91.0542,119.0491,65.0386,44.0131,72.0444	192.1388	$[M+H]^+$	-2.6
75	Difenoconazole	$C_{19}H_{17}Cl_2N_3O_3$	14.883	406.072	251.0025,252.9997,337.0393	406.0725	$[M+H]^+$	-1.23
76	Difenoxuron	$C_{16}H_{18}N_2O_3$	7.265	287.139	72.0444,123.0441,95.0491	287.1396	$[M+H]^+$	-2.09
77	Dimethachlor	$C_{13}H_{18}ClNO_2$	8.001	256.1099	148.1121,224.0837,105.0699	256.1104	$[M+H]^+$	-1.95
78	Dimethenamid	$C_{12}H_{18}ClNO_2S$	10.153	276.082	244.0557,111.0263,126.0372	276.0825	$[M+H]^+$	-1.81
79	Dimethomorph	$C_{21}H_{22}ClNO_4$	8.645	388.131	165.0546,301.0626,138.9945	388.1316	$[M+H]^+$	-1.55
80	Dimethylvinphos (E)	$C_{10}H_{10}Cl_3O_4P$	11.862	330.9455	127.0155,109.0049,169.9685	330.9461	$[M+H]^+$	-1.81
81	Dimethylvinphos (Z)	$C_{10}H_{10}Cl_3O_4P$	10.979	330.9455	127.0155,206.9344,204.9393	330.9461	$[M+H]^+$	-1.81
82	Dimetilan	$C_{10}H_{16}N_4O_3$	3.916	241.1295	72.0444,196.0717,56.0131	241.1301	$[M+H]^+$	-2.49
83	Diphenamid	$C_{16}H_{17}NO$	8.228	240.1383	134.0964,165.0699,152.0621	240.1388	$[M+H]^+$	-2.08
84	Dipropetryn	$C_{11}H_{21}N_5S$	11.861	256.1591	214.1121,172.0651,144.0338	256.1596	$[M+H]^+$	-1.95
85	Dipropyl Isocinchomeronate	$C_{13}H_{17}NO_4$	10.755	252.124	164.0706,210.0761,192.0655	252.1236	$[M+H]^+$	1.59

No.	Pesticides	Chemical formula	Retention Time (min)	Actual precursor ion(*m/z*)	Daughter ion(*m/z*)	Theoretical precursor ion(*m/z*)	Additive form	Mass deviation (ppm)
86	Dithiopyr	$C_{15}H_{16}F_5NO_2S_2$	17.166	402.0618	248.0351,354.0582,296.0363	402.0621	$[M+H]^+$	-0.75
87	Esprocarb	$C_{15}H_{23}NOS$	17.456	266.1575	91.0542,71.0855,196.0791,92.0576,72.089	266.1579	$[M+H]^+$	-1.5
88	Etaconazole	$C_{14}H_{15}Cl_2N_3O_2$	11.229	328.0614	256.0034,160.9753,55.0542	328.062	$[M+H]^+$	-1.83
89	Ethiofencarb-sulfone	$C_{11}H_{15}NO_4S$	3.522	258.0795	107.0491,77.0386,79.0542	258.08	$[M+H]^+$	-1.94
90	Ethiofencarb-sulfoxide	$C_{11}H_{15}NO_3S$	3.244	242.0845	107.0491,77.0386,79.0542	242.0851	$[M+H]^+$	-2.48
91	Ethoprophos	$C_8H_{19}O_2PS_2$	10.857	243.0637	96.9508,130.9385,172.9854	243.0642	$[M+H]^+$	-2.06
92	ethychlozate	$C_{11}H_{11}ClN_2O_2$	7.244	239.0583	165.0214,138.0105,110.9996	239.0587	$[M+H]^+$	-1.67
93	Etofenprox	$C_{25}H_{28}O_3$	19.608	394.2377	135.0804,177.1274,107.0491,183.0804,149.0961	394.2382	$[M+H]^+$	-1.27
94	Etoxazole	$C_{21}H_{23}F_2NO_2$	18.382	360.177	141.0146,57.0699,304.1132,177.1274,113.0197	360.1775	$[M+H]^+$	-1.39
95	Etrimfos	$C_{10}H_{17}N_2O_4PS$	14.981	293.0719	78.9943,265.0406,56.0495	293.0725	$[M+H]^+$	-2.05
96	Famphur	$C_{10}H_{16}NO_5PS_2$	9.96	326.028	93.0100,217.0073,280.9702	326.0286	$[M+H]^+$	-1.84
97	Fenamidone	$C_{17}H_{17}N_3OS$	11.381	312.1165	92.0495,236.1182,65.0386	312.1171	$[M+H]^+$	-1.92
98	Fenamiphos	$C_{13}H_{22}NO_3PS$	10.471	304.1131	201.9848,217.0083,234.0345	304.1136	$[M+H]^+$	-1.64
99	Fenamiphos-sulfone	$C_{13}H_{22}NO_5PS$	5.572	336.1029	266.0247,308.0716,108.0573	336.1035	$[M+H]^+$	-1.79
100	Fenamiphos-sulfoxide	$C_{13}H_{22}NO_4PS$	4.572	320.108	108.0573,233.0032,171.0474	320.1085	$[M+H]^+$	-1.56
101	Fenfuram	$C_{12}H_{11}NO_2$	6.969	202.0863	109.0284,53.0386,120.0444	202.0868	$[M+H]^+$	-2.47
102	Fenhexamid	$C_{14}H_{17}Cl_2NO_2$	11.54	302.0709	55.0542,97.1012,143.0128	302.0715	$[M+H]^+$	-1.99
103	Fenobucarb	$C_{12}H_{17}NO_2$	8.819	208.1332	77.0386,95.0491,57.0699	208.1338	$[M+H]^+$	-2.88
104	Fenothiocarb	$C_{13}H_{19}NO_2S$	13.27	254.1209	72.0444,160.0791,161.0819	254.1215	$[M+H]^+$	-2.36

No.	Pesticides	Chemical formula	Retention Time (min)	Actual precursor ion(*m*/*z*)	Daughter ion(*m*/*z*)	Theoretical precursor ion(*m*/*z*)	Additive form	Mass deviation (ppm)
105	Fenoxycarb	$C_{17}H_{19}NO_4$	13.373	302.1387	88.0393,116.0706,256.0968	302.1392	$[M+H]^+$	-1.65
106	Fenpiclonil	$C_{11}H_6Cl_2N_2$	8.991	236.9981	202.0292,140.0495,167.0604	236.9986	$[M+H]^+$	-2.11
107	Fenpyrazamine	$C_{17}H_{21}N_3O_2S$	11.839	332.1427	189.0897,216.1131,230.1288,231.1366,131.073	332.1433	$[M+H]^+$	-1.81
108	Fenpyroximate-(E)	$C_{24}H_{27}N_3O_4$	18.082	422.2074	366.1448,231.1002,138.0662,96.0682,57.0699	422.208	$[M+H]^+$	-1.42
109	Fenpyroximate-(Z)	$C_{24}H_{27}N_3O_4$	17.253	422.2074	366.1448,231.1002,138.0662,96.0682,57.0699	422.208	$[M+H]^+$	-1.42
110	Fenuron	$C_9H_{12}N_2O$	3.587	165.1022	72.0444,77.0386,56.0131	165.1028	$[M+H]^+$	-3.63
111	Ferimzone-(E)	$C_{15}H_{18}N_4$	5.757	255.1612	132.0808,124.0869,117.0573	255.161	$[M+H]^+$	0.78
112	Ferimzone-(Z)	$C_{15}H_{18}N_4$	5.534	255.1604	117.0573,132.0807,124.0869,96.0681	255.161	$[M+H]^+$	-2.35
113	Ferulic acid	$C_{10}H_{10}O_4$	5.758	193.0497	89.0389,117.0338,145.0288,177.0546	193.0501	$[M-H]^-$	-2.07
114	Flamprop-isopropyl	$C_{19}H_{19}ClFNO_3$	15.465	364.111	105.0335,77.0386,3054.0530	364.1116	$[M+H]^+$	-1.65
115	Flonicamid	$C_9H_6F_3N_3O$	3.29	230.0536	203.0427,98.0401,148.0369	230.0541	$[M+H]^+$	-2.17
116	Florasulam	$C_{12}H_8F_3N_5O_3S$	5.856	360.0373	129.0385,191.9914,130.0413,109.0318,128.0304	360.0378	$[M+H]^+$	-1.39
117	Fluacrypyrim	$C_{20}H_{21}F_3N_2O_5$	16.931	427.1475	145.0648,205.0859,117.0699	427.1481	$[M+H]^+$	-1.4
118	Fluazifop-butyl	$C_{19}H_{20}F_3NO_4$	17.862	384.1417	91.0542,282.0725,328.0791	384.1423	$[M+H]^+$	-1.56
119	Flufenacet	$C_{14}H_{13}F_4N_3O_2S$	13.479	364.0737	124.0557,152.0506,194.0976	364.0743	$[M+H]^+$	-1.65
120	Flufenoxuron	$C_{21}H_{11}ClF_6N_2O_3$	17.975	489.0446	156.0768,273.0340,182.0560	489.0441	$[M+H]^+$	1.02
121	Flufenpyr-ethyl	$C_{16}H_{13}ClF_4N_2O_4$	14.253	409.0573	307.0256,335.0205,337.0179	409.0578	$[M+H]^+$	-1.22

No.	Pesticides	Chemical formula	Retention Time (min)	Actual precursor ion(*m*/*z*)	Daughter ion(*m*/*z*)	Theoretical precursor ion(*m*/*z*)	Additive form	Mass deviation (ppm)
122	Flufiprole	$C_{16}H_{10}Cl_2F_6N_4OS$	16.647	508.0195	404.9950,376.0600,406.9922,423.9934,387.0289	508.02	$[M+NH_4]^+$	-0.98
123	Flumetsulam	$C_{12}H_9F_2N_5O_2S$	4.247	326.0518	129.0385,262.0899,128.0306	326.0523	$[M+H]^+$	-1.53
124	Flumiclorac-pentyl	$C_{21}H_{23}ClFNO_5$	17.702	441.1593	308.0484,354.0539,424.1321	441.1593	$[M+NH_4]^+$	0
125	Fluopyram	$C_{16}H_{11}ClF_6N_2O$	12.583	397.0537	173.0209,208.0135,145.0260	397.0542	$[M+H]^+$	-1.26
126	Fluoroglycofen-ethyl	$C_{18}H_{13}ClF_3NO_7$	17.141	465.0671	300.0034,343.9932,345.9908	465.0676	$[M+NH_4]^+$	-1.08
127	Fluquinconazole	$C_{16}H_8Cl_2FN_5O$	11.916	376.0163	306.9836,108.0244,349.0054	376.0168	$[M+H]^+$	-1.33
128	fluroxypyr-meptyl	$C_{15}H_{21}Cl_2FN_2O_3$	18.438	367.0986	254.9723,180.9725,208.9674,236.9617,178.9574	367.0992	$[M+H]^+$	-1.63
129	Flurprimidol	$C_{15}H_{15}F_3N_2O_2$	9.847	313.1158	269.0533,270.0611,185.0709	313.1164	$[M+H]^+$	-1.92
130	Flusilazole	$C_{16}H_{15}F_2N_3Si$	12.755	316.1076	247.0749,165.0697,219.0436	316.1082	$[M+H]^+$	-1.9
131	Flutolanil	$C_{17}H_{16}F_3NO_2$	13.319	324.1206	262.0663,65.0386,242.0600	324.1211	$[M+H]^+$	-1.54
132	Flutriafol	$C_{16}H_{13}F_2N_3O$	6.598	302.1099	70.0400,123.0241,109.0448	302.1105	$[M+H]^+$	-1.99
133	Fluxapyroxad	$C_{18}H_{12}F_5N_3O$	11.863	382.0973	342.0849,362.0911,234.0525	382.0979	$[M+H]^+$	-1.57
134	Fonofos	$C_{10}H_{15}OPS_2$	15.244	247.0375	80.9558,108.9871,62.9453	247.038	$[M+H]^+$	-2.02
135	Fosthiazate	$C_9H_{18}NO_3PS_2$	6.365	284.0538	104.0165,227.9909,199.9596	284.0544	$[M+H]^+$	-2.11
136	Furalaxyl	$C_{17}H_{19}NO_4$	9.818	302.1387	95.0128,242.1176,270.1125	302.1392	$[M+H]^+$	-1.65
137	Furathiocarb	$C_{18}H_{26}N_2O_5S$	17.53	383.1635	195.0474,162.0675,167.0525	383.1641	$[M+H]^+$	-1.57
138	Furmecyclox	$C_{14}H_{21}NO_3$	13.524	252.1594	55.0542,110.0600,170.0812	252.16	$[M+H]^+$	-2.38
139	Griseofulvin	$C_{17}H_{17}ClO_6$	7.242	353.0786	69.0335,165.0546,215.0106	353.0792	$[M+H]^+$	-1.7

No.	Pesticides	Chemical formula	Retention Time (min)	Actual precursor ion(*m/z*)	Daughter ion(*m/z*)	Theoretical precursor ion(*m/z*)	Additive form	Mass deviation (ppm)
140	Haloxyfop-2-ethoxy ethyl	$C_{19}H_{19}ClF_3NO_5$	17.305	434.0977	91.0542,316.0347,272.0080	434.0982	$[M+H]^+$	-1.15
141	Hexazinone	$C_{12}H_{20}N_4O_2$	4.78	253.1659	71.0604,171.0876,85.0760	253.1665	$[M+H]^+$	-2.37
142	Imazamethabenz-methyl	$C_{16}H_{20}N_2O_3$	4.912	289.1547	86.0964,144.0444,229.1335	289.1552	$[M+H]^+$	-1.73
143	Imicyafos	$C_{11}H_{21}N_4O_2PS$	4.717	305.1206	201.0536,235.0413,139.0978	305.1201	$[M+H]^+$	1.64
144	Indanofan	$C_{20}H_{17}ClO_3$	14.112	341.0941	175.0754,187.0754,323.0833	341.0944	$[M+H]^+$	-0.88
145	Iodosulfuron-methyl	$C_{14}H_{14}IN_5O_6S$	8.24	507.9782	167.0564,56.0495,141.0771,83.0240,260.9394	507.9788	$[M+H]^+$	-1.18
146	Ipconazole	$C_{18}H_{24}ClN_3O$	14.486	334.1681	70.0400,125.0153,71.0478	334.1686	$[M+H]^+$	-1.5
147	Isazofos	$C_9H_{17}ClN_3O_3PS$	13.636	314.049	119.9957,96.0508,162.0424	314.0495	$[M+H]^+$	-1.59
148	Isocarbamid	$C_8H_{15}N_3O_2$	3.561	186.1237	87.0553,70.0287,130.0611	186.1243	$[M+H]^+$	-3.22
149	isocarbophos	$C_{11}H_{16}NO_4PS$	7.91	290.061	105.0339,275.0582,138.9945	290.0616	$[M+H]^+$	-2.07
150	Isofenphos-Methyl	$C_{14}H_{22}NO_4PS$	15.288	332.1075	230.9875,121.0284,198.9613	332.1085	$[M+H]^+$	-3.01
151	Isomethiozin	$C_{12}H_{20}N_4OS$	13.793	269.1431	57.0699,200.0852,89.0168,116.0277,84.0808	269.1436	$[M+H]^+$	-1.86
152	Isoprothiolane	$C_{12}H_{18}O_4S_2$	12.712	291.0719	144.9776,231.0144,188.9675	291.0725	$[M+H]^+$	-2.06
153	Isoproturon	$C_{12}H_{18}N_2O$	6.875	207.1492	72.0444,46.0651,165.1022	207.1497	$[M+H]^+$	-2.41
154	Isoxadifen-ethyl	$C_{18}H_{17}NO_3$	14.828	296.1281	232.0757,203.0730,204.0808	296.1287	$[M+H]^+$	-2.03
155	Karbutilate	$C_{14}H_{21}N_3O_3$	5.701	280.1656	72.0444,181.0972,136.0393,93.0335,108.0444	280.1661	$[M+H]^+$	-1.78
156	Ligustilide	$C_{12}H_{14}O_2$	8.986	191.1067	91.0542,115.053	191.1072	$[M+H]^+$	-2.62
157	Mefenacet	$C_{16}H_{14}N_2O_2S$	11.378	299.0849	120.0808,148.0757,91.0542	299.0854	$[M+H]^+$	-1.67

No.	Pesticides	Chemical formula	Retention Time (min)	Actual precursor ion(*m*/*z*)	Daughter ion(*m*/*z*)	Theoretical precursor ion(*m*/*z*)	Additive form	Mass deviation (ppm)
158	Mefenpyr-diethyl	$C_{16}H_{18}Cl_2N_2O_4$	15.941	373.0716	159.9715,327.0298,144.9606	373.0722	$[M+H]^+$	-1.61
159	Mepronil	$C_{17}H_{19}NO_2$	12.675	270.1489	91.0542,119.0419,228.1019	270.1494	$[M+H]^+$	-1.85
160	Metaflumizone-(E)	$C_{24}H_{16}F_6N_4O_2$	17.303	507.125	330.0840,287.0790,221.0530,178.0470,116.0495	507.1256	$[M+H]^+$	-1.18
161	Metaflumizone-(Z)	$C_{24}H_{16}F_6N_4O_2$	17.028	507.125	330.0840,287.0790,221.0530,178.0470,116.0495	507.1256	$[M+H]^+$	-1.18
162	Metalaxyl	$C_{15}H_{21}NO_4$	6.914	280.1543	45.0335,192.1383,220.1332,160.1121,248.1281	280.1549	$[M+H]^+$	-2.14
163	Metamitron	$C_{10}H_{10}N_4O$	3.468	203.0927	104.0495,175.0978,77.0386	203.0933	$[M+H]^+$	-2.95
164	Metazachlor	$C_{14}H_{16}ClN_3O$	7.816	278.1055	134.0946,210.0662,105.0699	278.106	$[M+H]^+$	-1.8
165	Metconazole	$C_{17}H_{22}ClN_3O$	13.051	320.1524	70.0400,125.0153,71.0478,177.0447,95.0855	320.153	$[M+H]^+$	-1.87
166	Methamidophos	$C_2H_8NO_2PS$	1.586	142.0086	63.9947,94.0052,124.9821	142.0092	$[M+H]^+$	-4.23
167	Methfuroxam	$C_{14}H_{15}NO_2$	9.307	230.1176	137.0597,67.0542,111.0804	230.1181	$[M+H]^+$	-2.17
168	Methoprotryne	$C_{11}H_{21}N_5OS$	6.74	272.154	170.0495,198.0808,240.1277	272.1545	$[M+H]^+$	-1.84
169	Metobromuron	$C_9H_{11}BrN_2O_2$	7.32	259.0077	91.0416,169.9600,148.0631	259.0082	$[M+H]^+$	-1.93
170	Metribuzin	$C_8H_{14}N_4OS$	5.492	215.0961	49.0106,57.0699,187.1012	215.0967	$[M+H]^+$	-2.79
171	Metsulfuron-methyl	$C_{14}H_{15}N_5O_6S$	5.731	382.0816	167.0564,56.0495,135.0441	382.0821	$[M+H]^+$	-1.31
172	Mevinphos-(E)	$C_7H_{13}O_6P$	4.079	225.0527	193.0260,127.0155,109.0049,99.0441,67.0178	225.0523	$[M+H]^+$	1.78
173	Mevinphos-(Z)	$C_7H_{13}O_6P$	3.576	225.0527	193.0260,127.0155,109.0049,99.0441,67.0178	225.0523	$[M+H]^+$	1.78
174	Monocrotophos	$C_7H_{14}NO_5P$	2.768	224.0682	50.0287,127.0155,98.0600	224.0688	$[M+H]^+$	-2.68
175	Monolinuron	$C_9H_{11}ClN_2O_2$	6.841	215.0582	98.9996,126.0105,148.0631	215.0587	$[M+H]^+$	-2.32
176	Monuron	$C_9H_{11}ClN_2O$	5.102	199.0633	72.0444,98.9996,126.0105	199.0638	$[M+H]^+$	-2.51

No.	Pesticides	Chemical formula	Retention Time (min)	Actual precursor ion(*m*/*z*)	Daughter ion(*m*/*z*)	Theoretical precursor ion(*m*/*z*)	Additive form	Mass deviation (ppm)
177	Myclobutanil	$C_{15}H_{17}ClN_4$	11.053	289.1215	70.0400,125.0153,151.0309	289.122	$[M+H]^+$	-1.73
178	Neburon	$C_{12}H_{16}Cl_2N_2O$	13.555	275.0712	57.0699,88.1121,58.0287	275.0718	$[M+H]^+$	-2.18
179	Norflurazon	$C_{12}H_9ClF_3N_3O$	7.335	304.0459	140.0306,87.9949,160.0369	304.0464	$[M+H]^+$	-1.64
180	Noruron	$C_{13}H_{22}N_2O$	7.485	223.1811	72.0444,89.0709,135.1168	223.181	$[M+H]^+$	0.45
181	Novaluron	$C_{17}H_9ClF_8N_2O_4$	16.656	493.0196	158.0412,107.0491,310.0059	493.0201	$[M+H]^+$	-1.01
182	Nuarimol	$C_{17}H_{12}ClFN_2O$	8.377	315.0695	81.0447,243.0371,252.0819	315.07	$[M+H]^+$	-1.59
183	Ofurace	$C_{14}H_{16}ClNO_3$	6.909	282.0891	160.1121,254.0942,148.1121	282.0897	$[M+H]^+$	-2.13
184	Orbencarb	$C_{12}H_{16}ClNOS$	14.857	258.0714	125.0153,100.0757,89.0386,72.0444,44.0131	258.0719	$[M+H]^+$	-1.94
185	oxadiazon	$C_{15}H_{18}Cl_2N_2O_3$	17.647	345.0767	303.0298,184.9874,219.9563	345.0773	$[M+H]^+$	-1.74
186	Oxadixyl	$C_{14}H_{18}N_2O_4$	5.165	279.1339	132.0808,133.0886,219.1128	279.1345	$[M+H]^+$	-2.15
187	Oxasulfuron	$C_{17}H_{18}N_4O_6S$	5.416	407.102	150.0662,107.0604,124.0869	407.1025	$[M+H]^+$	-1.23
188	Oxycarboxin	$C_{12}H_{13}NO_4S$	4.579	268.0638	175.0060,146.9747,83.0128	268.0644	$[M+H]^+$	-2.24
189	oxydemeton-methyl	$C_6H_{15}O_4PS_2$	2.704	247.0222	109.0049,169.0083,124.9821,78.9943	247.0228	$[M+H]^+$	-2.43
190	Pebulate	$C_{10}H_{21}NOS$	15.321	204.1417	57.0699,128.1070,72.0444,176.1467,162.0947	204.1422	$[M+H]^+$	-2.45
191	Penconazole	$C_{13}H_{15}Cl_2N_3$	12.81	284.0716	70.0400,158.9763,122.9996,172.9919	284.0721	$[M+H]^+$	-1.76
192	Penflufen	$C_{18}H_{24}FN_3O$	13.331	318.1989	234.1037,141.0459,115.0666	318.1982	$[M+H]^+$	2.2
193	Penoxsulam	$C_{16}H_{14}F_5N_5O_5S$	8.129	484.0709	195.0751,194.0672,164.0567,166.0712,139.0500	484.0714	$[M+H]^+$	-1.03
194	Pentanochlor	$C_{13}H_{18}ClNO$	13.868	240.115	107.0730,142.0413,71.0855	240.1155	$[M+H]^+$	-2.08
195	Pethoxamid	$C_{16}H_{22}ClNO_2$	12.767	296.1412	91.0542,131.0855,250.0993	296.1417	$[M+H]^+$	-1.69
196	Phorate-oxon-sulfon	$C_7H_{17}O_5PS_2$	4.171	277.0337	110.9664,183.0239,154.9926	277.0333	$[M+H]^+$	1.44

No.	Pesticides	Chemical formula	Retention Time (min)	Actual precursor ion(*m*/*z*)	Daughter ion(*m*/*z*)	Theoretical precursor ion(*m*/*z*)	Additive form	Mass deviation (ppm)
	e							
197	Phorate-oxon-sulfoxide	$C_7H_{17}O_4PS_2$	3.457	261.0385	110.9664,243.0273,153.0134	261.0384	$[M+H]^+$	0.38
198	Phorate-Sulfone	$C_7H_{17}O_4PS_3$	8.564	293.0097	124.9821,247.0222,153.0134	293.0105	$[M+H]^+$	-2.73
199	Phorate-Sulfoxide	$C_7H_{17}O_3PS_3$	6.287	277.015	96.9508,142.9385,199.0011	277.0156	$[M+H]^+$	-2.17
200	Phosfolan	$C_7H_{14}NO_3PS_2$	4.126	256.0219	139.9566,167.9879,61.0106	256.0231	$[M+H]^+$	-4.69
201	Phosfolan-Methyl	$C_5H_{10}NO_3PS_2$	3.015	227.9911	61.0106,167.9879,109.0049	227.9918	$[M+H]^+$	-3.07
202	Phosphamidon-(E)	$C_{10}H_{19}ClNO_5P$	4.704	300.0762	226.9871,174.0680,132.0575,127.0155,104.0262,75.9949	300.0768	$[M+H]^+$	-2
203	Phosphamidon-(Z)	$C_{10}H_{19}ClNO_5P$	4.716	300.0762	226.9871,174.0680,132.0575,127.0155,100.0757,72.0444	300.0768	$[M+H]^+$	-2
204	Phoxim	$C_{12}H_{15}N_2O_3PS$	16.012	299.0616	77.0389,129.0447,96.9508,124.9821,153.0134	299.0619	$[M+H]^+$	-1
205	Piperonyl Butoxide	$C_{19}H_{30}O_5$	17.089	338.2092	119.0855,177.0910,147.0804,149.0597,178.0944	338.2093	$[M+H]^+$	-0.3
206	Promecarb	$C_{12}H_{17}NO_2$	10.305	208.1332	91.0542,109.0648,151.1117	208.1338	$[M+H]^+$	-2.88
207	Prometryn	$C_{10}H_{19}N_5S$	9.079	242.1434	68.0243,158.0495,200.0964,85.0509,74.0059	242.1439	$[M+H]^+$	-2.06
208	Propachlor	$C_{11}H_{14}ClNO$	7.692	212.0837	94.0651,170.0367,77.0386	212.0842	$[M+H]^+$	-2.36
209	Propazine	$C_9H_{16}ClN_5$	8.526	230.1167	79.0058,68.0243,104.0010	230.1172	$[M+H]^+$	-2.17
210	Propisochlor	$C_{15}H_{22}ClNO_2$	14.737	284.1412	224.0832,133.0886,73.0653	284.1417	$[M+H]^+$	-1.76
211	Propyzamide	$C_{12}H_{11}Cl_2NO$	11.571	256.029	189.9821,144.9606,172.9556	256.0296	$[M+H]^+$	-2.34
212	Prosulfocarb	$C_{14}H_{21}NOS$	16.842	252.1417	91.0542,128.1070,65.0386,86.0600,92.062	252.1422	$[M+H]^+$	-1.98

No.	Pesticides	Chemical formula	Retention Time (min)	Actual precursor ion(*m/z*)	Daughter ion(*m/z*)	Theoretical precursor ion(*m/z*)	Additive form	Mass deviation (ppm)
213	Prosulfuron	$C_{15}H_{16}F_3N_5O_4S$	10.157	420.0946	141.0771,167.0564,142.0792,128.0693,168.0586	420.0953	$[M+H]^+$	-1.67
214	Prothiofos	$C_{11}H_{15}Cl_2O_2PS_2$	19.287	344.9701	240.9041,160.9554,132.9604	344.9706	$[M+H]^+$	-1.45
215	Prothoate	$C_9H_{20}NO_3PS_2$	8.127	286.0695	96.9508,226.9960,142.9385,124.9821,74.0059	286.07	$[M+H]^+$	-1.75
216	Pyracarbolid	$C_{13}H_{15}NO_2$	6.001	218.1176	125.0597,55.0178,97.0284,83.0491,77.0386	218.1181	$[M+H]^+$	-2.29
217	Pyraclostrobin	$C_{19}H_{18}ClN_3O_4$	15.441	388.1059	194.0812,163.0628,133.0522	388.1064	$[M+H]^+$	-1.29
218	Pyraflufen-ethyl	$C_{15}H_{13}Cl_2F_3N_2O_4$	15.307	413.0277	253.0163,338.9904,260.9987	413.0283	$[M+H]^+$	-1.45
219	Pyrametostrobin	$C_{21}H_{23}N_3O_4$	12.852	382.1769	194.0812,163.0628,164.0706	382.1767	$[M+H]^+$	0.52
220	Pyrazosulfuron-ethyl	$C_{14}H_{18}N_6O_7S$	10.264	415.103	182.0560,139.0502,83.0240,369.0625,188.0124	415.1036	$[M+H]^+$	-1.45
221	Pyrazoxyfen	$C_{20}H_{16}Cl_2N_2O_3$	14.241	403.0612	105.0335,139.0502,172.9556	403.0616	$[M+H]^+$	-0.99
222	Pyributicarb	$C_{18}H_{22}N_2O_2S$	18.059	331.1475	181.0430,133.1012,108.0444	331.148	$[M+H]^+$	-1.51
223	Pyridaphenthion	$C_{14}H_{17}N_2O_4PS$	12.04	341.0719	92.0498,189.0659,96.9508	341.0725	$[M+H]^+$	-1.76
224	Pyriftalid	$C_{15}H_{14}N_2O_4S$	11.091	319.0747	139.0502,179.0161,83.0240	319.0753	$[M+H]^+$	-1.88
225	Pyriminobac-Methyl-(E)	$C_{17}H_{19}N_3O_6$	11.303	362.1347	330.1084,284.0666,256.0717,230.0686	362.1352	$[M+H]^+$	-1.38
226	Pyriminobac-Methyl-(Z)	$C_{17}H_{19}N_3O_6$	9.066	362.1347	330.1084,284.0666,256.0717,230.0686	362.1352	$[M+H]^+$	-1.38
227	Pyrimitate	$C_{11}H_{20}N_3O_3PS$	15.099	306.1036	154.0975,96.9508,71.0604	306.1041	$[M+H]^+$	-1.63
228	Pyriproxyfen	$C_{20}H_{19}NO_3$	17.77	322.1438	96.0444,78.0338,129.0699	322.1443	$[M+H]^+$	-1.55
229	Pyrisoxazole	$C_{16}H_{17}ClN_2O$	6.666	289.1105	120.0444,151.0866,80.0495	289.1108	$[M+H]^+$	-1.04

No.	Pesticides	Chemical formula	Retention Time (min)	Actual precursor ion(*m*/*z*)	Daughter ion(*m*/*z*)	Theoretical precursor ion(*m*/*z*)	Additive form	Mass deviation (ppm)
230	Pyroquilon	$C_{11}H_{11}NO$	4.995	174.0913	117.0573,132.0808,130.0651	174.0919	$[M+H]^+$	-3.45
231	Sebuthylazine-desethyl	$C_7H_{12}ClN_5$	4.595	202.0854	146.0228,68.0243,79.0058	202.0859	$[M+H]^+$	-2.47
232	Sedaxane	$C_{18}H_{19}F_2N_3O$	11.868	332.1575	159.0364,292.1444,312.1507	332.1574	$[M+H]^+$	0.3
233	Siduron	$C_{14}H_{20}N_2O$	8.988	233.1648	137.0709,94.0651,77.0386	233.1654	$[M+H]^+$	-2.57
234	Silthiofam	$C_{13}H_{21}NOSSi$	13.755	268.1186	73.0468,252.0873,139.0212	268.1191	$[M+H]^+$	-1.86
235	Sulfometuron-Methyl	$C_{15}H_{16}N_4O_5S$	5.872	365.0923	150.0662,151.0688,199.0060	365.092	$[M+H]^+$	0.82
236	Sulfotep	$C_8H_{20}O_5P_2S_2$	15.76	323.03	96.9508,171.0239,142.9926	323.0306	$[M+H]^+$	-1.86
237	Sulprofos	$C_{12}H_{19}O_2PS_3$	18.212	323.0358	218.9698,139.0212,247.0011	323.0363	$[M+H]^+$	-1.55
238	Tebuconazole	$C_{16}H_{22}ClN_3O$	11.766	308.1526	70.0400,125.0153,59.0491,127.0124,57.0699	308.153	$[M+H]^+$	-1.3
239	Tebufenpyrad	$C_{18}H_{24}ClN_3O$	16.917	334.1681	117.0209,145.0522,132.0934	334.1686	$[M+H]^+$	-1.5
240	Tebupirimfos	$C_{13}H_{23}N_2O_3PS$	17.854	319.124	153.1022,277.0770,249.0457,231.0352,57.0702	319.1245	$[M+H]^+$	-1.57
241	Tebutam	$C_{15}H_{23}NO$	12.813	234.1852	91.0542,65.0386,57.0699	234.1858	$[M+H]^+$	-2.56
242	Tebuthiuron	$C_9H_{16}N_4OS$	4.655	229.1118	172.0903,62.0059,116.0277	229.1123	$[M+H]^+$	-2.18
243	Temephos	$C_{16}H_{20}O_6P_2S_3$	17.954	466.997	124.9821,418.9936,404.9780	466.9975	$[M+H]^+$	-1.07
244	Tepraloxydim	$C_{17}H_{24}ClNO_4$	5.494	342.1467	166.0863,250.1438,251.1470	342.1472	$[M+H]^+$	-1.46
245	Terbucarb	$C_{17}H_{27}NO_2$	15.765	278.2116	109.0648,166.0863,222.1488	278.212	$[M+H]^+$	-1.44
246	Terbufos	$C_9H_{21}O_2PS_3$	17.142	289.0514	57.0699,103.0576,96.9508,187.0011,199.0011	289.052	$[M+H]^+$	-2.08
247	Terbufos-Sulfone	$C_9H_{21}O_4PS_3$	11.609	321.0412	275.0535,124.9821,142.9385	321.0418	$[M+H]^+$	-1.87
248	Terbufos-Sulfoxide	$C_9H_{21}O_3PS_3$	8.233	305.0465	130.9385,187.0011,158.9698	305.0469	$[M+H]^+$	-1.31

No.	Pesticides	Chemical formula	Retention Time (min)	Actual precursor ion(*m*/*z*)	Daughter ion(*m*/*z*)	Theoretical precursor ion(*m*/*z*)	Additive form	Mass deviation (ppm)
249	Terbumeton	$C_{10}H_{19}N_5O$	5.685	226.1662	170.1036,69.0083,57.0447	226.1668	$[M+H]^+$	-2.65
250	Terbuthylazine	$C_9H_{16}ClN_5$	9.285	230.1167	174.0541,68.0243,104.0010	230.1172	$[M+H]^+$	-2.17
251	Tetraconazole	$C_{13}H_{11}Cl_2F_4N_3O$	12.252	372.029	70.0400,158.9763,160.9734,159.9797,161.9767	372.0294	$[M+H]^+$	-1.08
252	Thenylchlor	$C_{16}H_{18}ClNO_2S$	12.973	324.0819	127.0212,128.0242,129.0179	324.0825	$[M+H]^+$	-1.85
253	Thiazafluron	$C_6H_7F_3N_4OS$	5.254	241.0365	74.0059,184.0151,111.0165	241.0371	$[M+H]^+$	-2.49
254	Thiazopyr	$C_{16}H_{17}F_5N_2O_2S$	15.769	397.0997	377.0941,355.0472,61.0106	397.1009	$[M+H]^+$	-3.02
255	Thiobencarb	$C_{12}H_{16}ClNOS$	15.174	258.0714	125.0153,100.0757,72.0444	258.0719	$[M+H]^+$	-1.94
256	Thiodicarb	$C_{10}H_{18}N_4O_4S_3$	5.891	355.0563	88.0189,107.9902,62.0025,46.9950,72.9981	355.0568	$[M+H]^+$	-1.41
257	Thionazin	$C_8H_{13}N_2O_3PS$	8.464	249.0456	174.9726,192.9831,124.9821,221.0144,153.0134	249.0463	$[M+H]^+$	-2.81
258	Tiocarbazil	$C_{16}H_{25}NOS$	18.526	280.173	91.0542,100.0757,57.0699,224.1104,92.0576	280.1735	$[M+H]^+$	-1.78
259	Tolclofos-methyl	$C_9H_{11}Cl_2O_3PS$	15.665	300.9616	124.9821,110.9994,174.9710	300.9622	$[M+H]^+$	-1.99
260	Tolfenpyrad	$C_{21}H_{22}ClN_3O_2$	17.162	384.1477	197.0961,117.0214,145.0527,198.0995,171.032	384.1479	$[M+H]^+$	-0.52
261	Triadimefon	$C_{14}H_{16}ClN_3O_2$	11.208	294.1004	69.0699,197.0714,225.0659	294.1009	$[M+H]^+$	-1.7
262	Triadimenol	$C_{14}H_{18}ClN_3O_2$	8.68	296.1158	99.0804,227.0833,70.0399	296.1166	$[M+H]^+$	-2.7
263	Triamiphos	$C_{12}H_{19}N_6OP$	5.584	295.1431	135.0679,92.0260,73.0760	295.1436	$[M+H]^+$	-1.69
264	Triasulfuron	$C_{14}H_{16}ClN_5O_5S$	6.204	402.0633	141.0771,167.0537,56.0495	402.0639	$[M+H]^+$	-1.49
265	Triazophos	$C_{12}H_{16}N_3O_3PS$	12.779	314.0723	119.0604,162.0662,96.9508	314.0728	$[M+H]^+$	-1.59
266	Tribufos	$C_{12}H_{27}OPS_3$	19.084	315.1029	168.9905,57.0699,112.9279	315.104	$[M+H]^+$	-3.49
267	Trietazine	$C_9H_{16}ClN_5$	11.866	230.1167	71.0604,68.0243,104.0010	230.1172	$[M+H]^+$	-2.17

No.	Pesticides	Chemical formula	Retention Time (min)	Actual precursor ion(*m/z*)	Daughter ion(*m/z*)	Theoretical precursor ion(*m/z*)	Additive form	Mass deviation (ppm)
268	Trifloxystrobin	$C_{20}H_{19}F_3N_2O_4$	16.944	409.137	145.0260,186.0525,116.0495	409.1375	$[M+H]^+$	-1.22
269	Triflumizole	$C_{15}H_{15}ClF_3N_3O$	15.287	346.0929	69.0447,73.0648,278.0554,280.0527,205.9979	346.0934	$[M+H]^+$	-1.44
270	Triticonazole	$C_{17}H_{20}ClN_3O$	9.554	318.1368	70.0400,125.0153,59.0496	318.1373	$[M+H]^+$	-1.57
271	Uniconazole	$C_{15}H_{18}ClN_3O$	10.784	292.1213	274.1106,218.0480,125.0153,70.0400	292.1217	$[M+H]^+$	-1.37
272	Zoxamide	$C_{14}H_{16}Cl_3NO_2$	15.34	336.0319	186.9712,158.9763,203.9973	336.0325	$[M+H]^+$	-1.79

Table S2. Methodological validation of 270 pesticides in *AS* decoction

No.	Compounds	Linear range (μg/L)	Linearequation	R^2	LOD (μg/L)	LOQ (μg/L)	Matrix effect (%)	Recovery (LOQ)	RSD (LOQ)	Recovery (2×LOQ)	RSD (2×LOQ)	Recovery 10×LOQ	RSD (10×LOQ)	RSDwR (10×LOQ)
1	1-naphthyl acetamide	5~100	y=15499.804952x-25639.579047	0.9983	1.5	5	-51.27	104.12	6.94	93.44	13.09	104.05	2.83	5.26
2	2,6-Dichlorobenzamide	5~100	y=1785.463921x-168.595496	0.9994	1.5	5	-69.11	117.88	8.62	116.69	18.37	117.33	5.04	3.61
3	3,4,5-Trimethacarb	5~100	y=54338.690782x-51276.356292	0.9996	1.5	5	136.59	109.91	7.71	116.23	3.69	115.09	1.25	0.03
4	Acetamiprid-(E)	20~100	y=436.670953x+446.763662	0.9934	6	20	-70.75	82.39	11.03	97.1	7.38	93.31	9.19	3.70
5	Acetamiprid-(Z)	20~100	y=26756.341894x-3432.454327	0.9964	6	20	-53.09	79.1	8.27	104.2	9.33	97.48	5.27	1.87
6	Aldicarb	50~200	y=889.455621x-15626.546264	0.9865	15	50	-42.47	99.35	12.59	83.29	9.24	102.49	8.36	6.73
7	Aldicarb-sulfone	5~100	y=3361.678663x-7684.794863	0.9978	1.5	5	-52.41	77.17	19.8	83.67	4.77	91.36	11.57	6.54
8	Aldicarb-sulfoxide	5~100	y=4968.646737x-4550.805462	0.9985	1.5	5	46.35	93.72	5.69	105.94	2.75	105.67	7.33	5.90
9	Ametryn	5~100	y=175239.863884x-732730.565582	0.9982	1.5	5	-31.25	104.97	7.54	94.9	1.69	81.5	0.82	1.33
10	Aminocarb	5~100	y=40084.426593x-211680.562299	0.9964	1.5	5	-42.79	116.23	11.82	117.42	5.29	116.52	2.02	9.47
11	Ancymidol	5~100	y=20772.348284x-43564.211068	0.9965	1.5	5	-77.38	74.94	8.54	114.16	9.41	109.62	2.54	4.02
12	Anilofos	5~100	y=74544.840739x-271313.571467	0.9991	1.5	5	-21.54	112.14	5.42	111.74	2.68	104.95	1.16	0.08
13	Aramite	10~100	y=53.231348x+1217.879175	0.9934	3	10	-70.75	108.65	15.93	105.83	9.44	95.28	10.38	6.90
14	Atraton	5~100	y=153999.765229x-662264.283094	0.9984	1.5	5	-40.63	106.8	9.41	96.13	5.11	84	1.81	0.95
15	Atrazine-desisopropyl	5~100	y=9629.989315x-19699.062077	0.9976	1.5	5	-80.61	102.77	11.2	96.14	9.46	95.6	3.67	4.45
16	Azaconazole	5~100	y=1454.104942x-3659.674434	0.9935	1.5	5	-98	77.16	16.87	108.91	16.91	81.82	4.12	0.24
17	azadirachtin	5~100	y=10263.374234x-4491.282985	0.9965	1.5	5	197.76	112.62	6.36	116.13	4.5	99.73	3.37	2.74
18	Aziprotryne	5~100	y=17325.468990x-66276.693613	0.9961	1.5	5	-49.07	87.4	12.35	87.52	8.67	111.49	10.03	5.15
19	Azoxystrobin-(E)	10~100	y=1720.514628x+9724.704117	0.9845	3	10	-50.35	113.72	10.93	103.39	5.39	102.55	6.3	7.06

No.	Compounds	Linear range (μg/L)	Linearequation	R^2	LOD (μg/L)	LOQ (μg/L)	Matrix effect (%)	Recovery (LOQ)	RSD (LOQ)	Recovery (2×LOQ)	RSD (2×LOQ)	Recovery 10×LOQ	RSD (10×LOQ)	RSDwR (10×LOQ)
20	Azoxystrobin-(Z)	10~100	y=10915.900857x-23930.850928	0.9994	3	10	-20.81	106.93	12.39	98.42	9.05	100.82	10.42	7.37
21	Beflubutamid	10~100	y=14846.265694x-20239.841845	0.9948	3	10	-30.88	103.23	3.98	101.48	4.82	95.2	8.41	1.42
22	Benalaxyl	5~100	y=112547.685768x-593443.585811	0.9966	1.5	5	-21.18	115.8	7.33	118.02	4.01	110.57	2.04	0.82
23	Bendiocarb	5~100	y=321.459405x-1354.083646	0.9845	1.5	5	-50.35	103.57	9.87	79.33	4.61	114.04	10.6	6.98
24	Benodanil	5~100	y=46412.233510x-149240.290093	0.9983	1.5	5	-31.38	118.25	7.42	114.53	1.84	115.17	1.67	6.12
25	Bensulide	5~100	y=17755.308563x-26582.696923	0.9955	1.5	5	26.41	110.2	7.15	114.87	4.87	113.35	1.82	2.25
26	Benzovindiflupyr	5~100	y=63932.462830x-177206.044022	0.9995	1.5	5	-17.16	115.4	11.23	116.97	2.69	91.86	1.14	6.88
27	Bitertanol	5~100	y=5266.491229x+7879.228358	0.9975	1.5	5	-26.91	117.05	4.91	118.96	3.18	101.33	0.69	4.24
28	Blasticidin-S	10~100	y=145.174924x+1825.342577	0.9945	3	10	183.92	113.66	17.13	105.32	9.05	109.3	12.49	3.03
29	Boscalid	5~100	y=14786.222574x-25697.724367	0.9994	1.5	5	-18.44	109.54	9.59	108.59	4.14	92.35	1.35	8.64
30	Bromacil	50~200	y=416.147109x-1552.160318	0.9873	15	50	-62	105.95	7.14	96.21	4.29	102.48	5.83	4.49
31	Bromfenvinfos-Methyl	5~100	y=4300.597280x-494.024458	0.9984	1.5	5	-28.89	110.52	11.03	114.03	6.12	112.61	3	3.53
32	Bromobutide	10~100	y=8052.079275x-23961.161783	0.9991	3	10	-24.41	83.95	18.81	90.31	10.3	98.2	12.34	0.48
33	Bromuconazole	5~100	y=6646.730797x-20271.105616	0.997	1.5	5	-46.67	115.26	8.5	73.95	4.24	72.25	1.76	2.88
34	Bupirimate	5~100	y=208614.092231x-1015652.528482	0.997	1.5	5	-29.77	114.02	7.74	104.77	3.2	86.94	1.92	0.74
35	Butafenacil	5~100	y=39293.876763x-285347.893909	0.9979	1.5	5	-3.86	116.69	10.89	113.8	2.2	111.18	0.75	3.85
36	Butamifos	10~100	y=7496.203039x-14969.216879	0.9993	3	10	-16.72	89.4	15.35	106.38	11.84	101.39	5.03	2.36
37	Cadusafos	5~100	y=2734.459743x-3443.745594	0.9994	1.5	5	-60.08	103.77	19.76	75.09	8.58	111.72	15.18	1.54
38	Cafenstrole	10~100	y=53930.932418x-284320.768965	0.9944	3	10	179.6	116.44	17.38	92.38	9.03	99.28	7.04	0.10
39	Carbendazim	5~100	y=3563.346115x-5342.446524	0.9975	1.5	5	-48.32	116.49	6.47	118.36	3.72	114.51	0.69	7.57
40	Carbofuran	10~100	y=4645.660968x-56.953576	0.9983	3	10	-73.99	113.83	1.97	107.2	4.96	103.89	3.02	2.62
41	Carbofuran-3-Hydroxy	10~100	y=2242.424543x-456.8978512	0.9995	3	10	-64.73	91.34	4.95	94.95	5.2	85.79	2.3	6.64

No.	Compounds	Linear range (μg/L)	Linearequation	R^2	LOD (μg/L)	LOQ (μg/L)	Matrix effect (%)	Recovery (LOQ)	RSD (LOQ)	Recovery (2×LOQ)	RSD (2×LOQ)	Recovery 10×LOQ	RSD (10×LOQ)	RSDwR (10×LOQ)
42	Carfentrazone-ethyl	10~100	y=17680.829768x-59208.518302	0.9991	3	10	-17.51	119.98	5.42	109.69	7.83	106.4	4.93	8.54
43	Carpropamid	10~100	y=1834.871404x-10831.038294	0.9994	3	10	-20.81	77.03	6.3	91.57	4.91	98.29	2.93	6.97
44	Chlordimeform	5~100	y=2313.094792x-5235.038293	0.9925	1.5	5	-53.32	72.51	8.73	76.17	6.06	78.57	11.24	8.55
45	Chlorfenvinphos-(E)	10~100	y=14754.543980x-754.672574	0.9934	3	10	38.51	75.38	9.35	92.04	6.95	85.29	3.81	3.43
46	Chlorfenvinphos-(Z)	10~100	y=70262.962287x-292865.366301	0.9992	3	10	-33.29	84.92	6.09	89.47	5.87	98.5	2.03	7.78
47	Chloridazon	5~100	y=28994.735628x-89366.061583	0.9989	1.5	5	-76.17	119.05	5.3	117.22	1.64	114.18	1.16	2.27
48	Chlorimuron-ethyl	10~100	y=2054.257741x-4525.768956	0.9868	3	10	24.45	95.83	16.45	89.86	12.84	110.84	5.39	7.81
49	Chlorpyrifos	5~100	y=32423.468572x-6462.466812	0.9945	1.5	5	-39.81	112.79	5.62	115.95	5.93	106.66	1.92	8.13
50	Chlorsulfuron	5~100	y=535.089414x-5352.058195	0.9934	1.5	5	35.97	91.49	5.04	70.7	13.7	84.87	16.04	9.02
51	Clodinafop-propargyl	10~100	y=30021.672517x-42975.242293	0.9978	3	10	-17.8	118.6	4.62	107.37	5.82	97.4	2.74	9.55
52	Clomazone	10~100	y=21377.476038x-62470.320845	0.9985	3	10	-23.51	119.1	6.97	112.6	5.38	98.2	3.7	6.85
53	Cloransulam-methyl	10~100	y=15324.951397x-39954.546014	0.9987	3	10	58.57	113.92	2.83	90.47	4.76	113.85	3.57	3.44
54	Crufomate	5~100	y=101618.948077x-395856.464379	0.9993	1.5	5	-17.49	110.14	6.84	115.62	3.13	99.5	0.94	8.38
55	Cyanazine	5~100	y=22380.387993x-87039.747213	0.9983	1.5	5	-25.01	116.98	10.13	110.05	4.4	113.26	1.59	2.92
56	Cyazofamid	10~100	y=4999.640666x-11846.077477	0.999	3	10	-26.89	112.87	7.35	109.41	5.82	107.29	4.3	7.05
57	Cycloate	10~100	y=7067.546733x-23115.887068	0.9982	3	10	-16.49	113.98	7.77	90.95	5.09	108.29	4.14	6.16
58	Cycluron	5~100	y=69139.420452x-162495.658576	0.9975	1.5	5	-47.44	96.55	6.76	116.15	3.72	101.97	0.59	9.16
59	Cyenopyrafen	10~100	y=398363.839963x-1551304.649380	0.9984	3	10	-14.12	112.34	5.49	108.24	6.39	102.59	3.52	8.45
60	Cyflufenamid	5~100	y=33725.535816x-18841.125301	0.9974	1.5	5	-20.38	116.1	9.98	115.28	1.9	119.53	1.77	7.80
61	Cymiazole	10~100	y=54760.284785x-242415.435191	0.9993	3	10	-72.01	72.07	9.31	85.71	6.5	103.28	3.1	3.35
62	Cymoxanil	5~100	y=68662.678362x+5456.676325	0.9965	1.5	5	-19.84	110.81	8.78	112.64	8.96	108.97	5.58	0.79
63	Cyprazine	10~100	y=34653.018900x-106511.681345	0.9953	3	10	-69.17	106.38	2.09	112.57	4.81	97.25	3.04	9.46

No.	Compounds	Linear range (μg/L)	Linearequation	R^2	LOD (μg/L)	LOQ (μg/L)	Matrix effect (%)	Recovery (LOQ)	RSD (LOQ)	Recovery (2×LOQ)	RSD (2×LOQ)	Recovery 10×LOQ	RSD (10×LOQ)	RSDwR (10×LOQ)
64	Cyproconazole	10~100	y=47085.683454x-67999.219972	0.9986	3	10	-47.56	116.37	6.3	109.45	4.82	101.39	7.39	9.74
65	Cyprofuram	5~100	y=21184.696407x-81209.936277	0.9989	1.5	5	-21.92	111.52	12.75	115.78	6.43	113.32	2.05	5.12
66	Daimuron	5~100	y=63789.527534x-398132.618983	0.9959	1.5	5	-17	118.51	8.12	116.68	3.54	109.81	0.71	6.50
67	Demeton	5~100	y=1653.974621x-863.624863	0.989	1.5	5	-42.71	71.55	19.91	81.97	19.24	100.07	16.82	8.74
68	Desethylterbuthylazine	5~100	y=36178.268063x-134803.519293	0.9987	1.5	5	-28.26	99.41	8.31	98.3	4.5	82.44	1.89	3.52
69	Desmetryn	5~100	y=206968.770656x-846422.483887	0.9975	1.5	5	-52.14	75.21	6.42	83.69	1.78	70.04	1.13	4.34
70	Diazinon	5~100	y=102979.781487x-423943.289002	0.9983	1.5	5	-34.7	111.01	10.73	111.41	3.94	118.38	1.07	9.05
71	Dichlormid	10~100	y=556.893811x+1243.354878	0.9973	3	10	-41.19	117.54	11.53	115.06	8.95	95.29	5.93	7.37
72	Diclocymet	10~100	y=1707.795105x+6965.213832	0.981	3	10	-35	113.84	14.87	108.48	10.93	109.37	4.82	8.91
73	Diclosulam	10~100	y=8392.707222x-16045.549874	0.9996	3	10	71.47	114.27	7.88	107.57	5.92	103.28	8.3	1.49
74	Diethyltoluamide	10~100	y=134207.832989x-342529.965948	0.9987	3	10	-28.42	113.54	4.78	115.72	5.03	95.59	6.58	5.77
75	Difenoconazole	5~100	y=83061.362006x-92719.080069	0.9981	1.5	5	-32.91	112.17	8.04	110.68	2.92	89.05	1.38	7.02
76	Difenoxuron	5~100	y=136864.839353x-594279.244263	0.9983	1.5	5	-28.43	117.46	5.37	118.95	2.49	96.42	0.68	9.85
77	Dimethachlor	10~100	y=41132.449007x-117220.823167	0.9995	3	10	-27.5	117.89	2.4	107.37	3.59	86.39	5.05	0.39
78	Dimethenamid	5~100	y=21550.992736x-76482.973417	0.9968	1.5	5	-55.79	117.71	11.16	106.45	4.01	117.75	3.02	1.26
79	Dimethomorph	10~100	y=25770.978306x-75537.800893	0.998	3	10	-7.03	116.81	6	112.59	4.82	97.2	6.53	7.33
80	Dimethylvinphos (E)	30~100	y=7047.093792x-15988.094476	0.9993	10	30	-8.96	91.71	19.25	75.1	10.42	108.41	9.82	4.64
81	Dimethylvinphos (Z)	30~100	y=9589.612108x-9653.119782	0.9982	10	30	-23.32	84.22	13.29	80.04	7.38	94.2	7.29	5.63
82	Dimetilan	5~100	y=20955.278195x-103174.063442	0.9991	1.5	5	-62.98	115.12	12.99	115.76	3.19	112.25	0.52	6.11
83	Diphenamid	10~100	y=162703.508640x-746512.808622	0.998	3	10	-19.59	117.38	5.65	110.95	7.06	103.21	6.19	8.27
84	Dipropetryn	5~100	y=259068.836275x-1471532.049807	0.994	1.5	5	-26.72	119.32	14.56	115.88	5.01	104.52	2.34	3.27
85	Dipropyl	10~100	y=506.028941x+514.050379	0.9992	3	10	-33.29	117.53	2.18	114.87	5.82	107.48	3.21	4.90

No.	Compounds	Linear range (μg/L)	Linearequation	R^2	LOD (μg/L)	LOQ (μg/L)	Matrix effect (%)	Recovery (LOQ)	RSD (LOQ)	Recovery (2×LOQ)	RSD (2×LOQ)	Recovery 10×LOQ	RSD (10×LOQ)	RSDwR (10×LOQ)
	Isocinchomeronate													
86	Dithiopyr	10~100	y=3609.411689x+13568.508480	0.9993	3	10	-67.72	113.52	3.31	109.7	4.16	96.18	5.03	4.52
88	Esprocarb	5~100	y=28789.249701x+15283.766662	0.9985	1.5	5	-69.71	118.52	9.55	116.49	5.08	103.78	1.83	6.60
89	Etaconazole	5~100	y=50856.396635x-71117.255795	0.9972	1.5	5	-12.3	113.59	5.16	113.11	2.47	101.72	12.75	7.03
90	Ethiofencarb-sulfone	10~100	y=4745.048887x+2821.377372	0.9999	3	10	-56.45	117.74	3.92	114.79	5.83	102.38	3.07	1.33
91	Ethiofencarb-sulfoxide	5~100	y=8978.393492x+23345.933234	0.9997	1.5	5	-25.66	117.18	12.73	89.78	10.84	95.64	5.24	7.88
92	Ethoprophos	5~100	y=89458.432848x-865.466095	0.9992	1.5	5	-49.41	74.34	12.97	74.14	3.04	89.16	17.38	13.72
93	ethychlozate	5~100	y=10168.309174x-16433.938356	0.9999	1.5	5	-23.47	115.61	13.62	112.04	9.48	101.17	2.74	7.24
94	Etofenprox	5~100	y=2896.493722x-11451.629981	0.9845	1.5	5	-88.57	111.05	13.53	110.07	16.37	88.19	5.15	3.55
95	Etoxazole	5~100	y=383687.828682x-1848685.200080	0.9981	1.5	5	-25.61	119.82	19.66	118.02	9.51	102.88	0.42	0.59
96	Etrimfos	5~100	y=96441.830305x-423384.015321	0.9986	1.5	5	-24.58	118.71	11.3	115.13	3.29	102.46	0.76	6.75
97	Famphur	5~100	y=655.038149x+3417.038421	0.9993	1.5	5	-8.96	111.35	7.2	111.31	2.58	117.82	1.28	1.34
98	Fenamidone	5~100	y=45391.743638x-116937.664657	0.9989	1.5	5	-59.39	116.66	4.34	116.98	2.8	110.92	0.77	9.80
99	Fenamiphos	5~100	y=27867.514758x-2567.936746	0.9996	1.5	5	-29.95	99.46	13.3	90.71	18.54	72.15	8.12	1.35
100	Fenamiphos-sulfone	5~100	y=18978.477903x-2786.793473	0.9996	1.5	5	-26.35	75.5	8.75	105.48	10.73	85.46	12.05	6.08
101	Fenamiphos-sulfoxide	5~100	y=327857.434790x-14276.796463	0.9996	1.5	5	-19.61	73.04	18.06	75.41	2.62	71.4	10.48	7.63
102	Fenfuram	5~100	y=26973.955437x-31098.217631	0.9985	1.5	5	-50.26	92.56	10.96	109.9	4.17	93.06	3.29	0.57
103	Fenhexamid	5~100	y=8833.061288x+727.056754	0.9982	1.5	5	-28.45	101.37	7.35	116.76	3.19	93.09	1.63	7.19
104	Fenobucarb	5~100	y=15271.027605x-35103.369140	0.9959	1.5	5	49.25	113.65	5.27	113.29	2.53	92.43	17.12	14.69
105	Fenothiocarb	10~100	y=16676.787352x-5677.345686	0.9959	3	10	-29.99	113.04	4.97	109.52	6.34	101.48	3.1	4.29
106	Fenoxycarb	10~100	y=18242.073967x-3251.084667	0.9982	3	10	-23.32	94.47	7.61	108.83	4.02	92.5	5.81	4.72
107	Fenpiclonil	5~100	y=917.287788x+206.934457	0.9967	1.5	5	-63.11	72.19	13.88	118.08	18.33	107.47	10.48	7.02

No.	Compounds	Linear range (μg/L)	Linearequation	R^2	LOD (μg/L)	LOQ (μg/L)	Matrix effect (%)	Recovery (LOQ)	RSD (LOQ)	Recovery (2×LOQ)	RSD (2×LOQ)	Recovery 10×LOQ	RSD (10×LOQ)	RSDwR (10×LOQ)
108	Fenpyrazamine	10~100	y=108416.210312x-495242.413371	0.9984	3	10	-9.94	115.6	3.63	108.79	4.19	96.49	6.39	2.72
109	Fenpyroximate-(E)	30~100	y=182097.436827x-791975.940944	0.9986	10	30	-10.54	85.92	15.49	95.29	10.29	93.37	9.03	6.57
110	Fenpyroximate-(Z)	30~100	y=34924.492769x-95920.078246	0.9994	10	30	-6.55	78.3	10.28	84.47	6.39	89.02	4.2	7.45
111	Fenuron	5~100	y=22067.144383x-72055.136088	0.9971	1.5	5	-60.43	115.37	11.99	110.55	6.31	117.56	1.64	9.83
112	Ferimzone-(E)	5~100	y=14612.109865x-26776.035619	0.9989	1.5	5	14.73	105.34	15.05	110.82	12.05	98.02	9.49	4.41
113	Ferimzone-(Z)	5~100	y=20217.160791x+150002.767330	0.9818	1.5	5	-32.89	112.68	10.82	108.28	8.02	107.28	8.2	2.94
114	Flamprop-isopropyl	10~100	y=4292.048574x+3904.561304	0.9994	3	10	-6.55	101.06	4.18	110.56	2.75	98.04	2.42	4.45
115	Flonicamid	10~100	y=982.674180x+6788.398941	0.9893	3	10	-40.96	111.43	2.57	114.93	1.83	106.42	8.2	3.76
116	Florasulam	5~100	y=5234.086294x-13409.956284	0.9989	1.5	5	14.73	83.89	12.12	84.66	5.05	70.83	3.29	2.43
117	Fluacrypyrim	10~100	y=4870.268711x-7903.003174	0.9991	3	10	-23.58	113.55	3.34	110.89	5.73	108.59	6.83	5.86
118	Fluazifop-butyl	10~100	y=158891.785572x-666850.994544	0.9985	3	10	-26.18	116.05	11.95	105.82	7.94	107.25	8.93	5.16
119	Flufenacet	10~100	y=14476.392321x-13019.791908	0.9958	3	10	-12.97	119.33	4.7	108.78	2.71	96.49	4.61	7.30
120	Flufenoxuron	10~100	y=1874.973657x-401.705959	0.9891	3	10	-32	113.97	7.85	93.79	6.59	104.69	4.82	8.92
121	Flufenpyr-ethyl	10~100	y=31553.344538x-98718.130285	0.9989	3	10	-13.8	115.87	2.06	104.9	3.83	97.42	5.03	5.22
122	Flufiprole	5~100	y=3395.337646x+10399.113022	0.9949	1.5	5	-33.98	111.35	5.91	101.01	9.96	115.41	3.51	5.32
123	Flumetsulam	10~100	y=1324.048392x-5259.058395	0.9818	3	10	-32.89	113.85	4.52	92.89	2.4	102.46	8.39	6.43
124	Flumiclorac-pentyl	5~100	y=12067.314692x-19882.053111	0.9957	1.5	5	-50.16	96.16	8.8	110.31	4.84	103.76	2.27	6.60
125	Fluopyram	5~100	y=94994.305228x-472254.133974	0.9991	1.5	5	-36.21	113.16	7.28	114.37	2.22	114.17	0.98	4.56
126	Fluoroglycofen-ethyl	5~100	y=615.949313x-2287.218872	0.9974	1.5	5	-75.45	74.22	14.52	118.66	16.4	112.31	7.28	7.08
127	Fluquinconazole	5~100	y=3731.690771x+226.408231	0.9979	1.5	5	-49.82	77.06	8.39	111.77	5.01	96.84	2.49	4.30
128	fluroxypyr-meptyl	5~100	y=1857.407331x-4084.910703	0.9859	1.5	5	-56.11	77.54	13.65	114.05	12.68	76.97	6.53	2.37
129	Flurprimidol	5~100	y=16440.275602x+10461.553257	0.9962	1.5	5	-78.37	75.57	7.3	118.39	4.75	110.31	1.5	6.37

No.	Compounds	Linear range (μg/L)	Linearequation	R^2	LOD (μg/L)	LOQ (μg/L)	Matrix effect (%)	Recovery (LOQ)	RSD (LOQ)	Recovery (2×LOQ)	RSD (2×LOQ)	Recovery 10×LOQ	RSD (10×LOQ)	RSDwR (10×LOQ)
130	Flusilazole	5~100	y=59685.474390x-76859.266717	0.9974	1.5	5	-62.68	86.94	2.15	105.53	1.27	110.29	0.64	1.86
131	Flutolanil	5~100	y=446.084935x+535.598105	0.9991	1.5	5	-35.95	113.32	9.82	111.81	4.91	113.32	1.4	1.75
132	Flutriafol	10~100	y=11421.506146x+5404.217162	0.9905	3	10	-71.57	113.64	8.54	106.32	6.48	95.39	9.2	4.35
133	Fluxapyroxad	5~100	y=23840.606892x-46501.953648	0.9974	1.5	5	-42.67	95.68	3.32	118.71	5.33	98.56	1.06	3.21
134	Fonofos	5~100	y=393732.084864x-12907.984625	0.9932	1.5	5	-68.9	80.47	8.48	81.97	8.67	81.2	1.02	5.47
135	Fosthiazate	10~100	y=47568.863658x-17564.784763	0.9965	3	10	-10.07	114.08	13.19	107.28	9.2	94.3	12.5	6.57
136	Furalaxyl	5~100	y=592.084729x-233.673094	0.9872	1.5	5	-22.27	115.45	16.14	119.65	5.13	116.77	5.51	9.95
137	Furathiocarb	5~100	y=44073.374753x-152006.032152	0.9953	1.5	5	-68.75	86.91	4.37	74.7	2.38	97.83	1.28	6.06
138	Furmecyclox	5~100	y=49476.884569x-130765.618015	0.9977	1.5	5	-36.59	118.73	14.08	104.26	14.31	82.33	6.75	8.90
139	Griseofulvin	5~100	y=10384.972640x-5213.097412	0.9988	1.5	5	-1.99	117.45	5.59	112.04	2.42	112.71	1	5.96
140	Haloxyfop-2-ethoxyethyl	5~100	y=16341.084963x-13205.904803	0.9971	1.5	5	-56.16	111.43	7.53	114.03	2.39	103.1	1.4	2.13
141	Hexazinone	5~100	y=85238.740132x-313652.927846	0.9985	1.5	5	-33.61	118.13	7.49	117.73	3.56	109.82	1.52	2.73
142	Imazamethabenz-methyl	5~100	y=169393.969534x-789349.349214	0.9982	1.5	5	-30.34	118.85	8.44	112.67	3.33	109.38	0.6	2.76
143	Imicyafos	10~100	y=124399.892575x-591011.469222	0.998	3	10	-26.4	110.37	5.57	93.97	4.38	105.39	9.57	5.07
144	Indanofan	10~100	y=12937.967493x-12409.975183	0.9942	3	10	-40.24	118.98	10.37	110.64	7.92	103.17	4.3	2.85
87	Iodosulfuron-methyl	5~100	y=34818.001324x-51322.620468	0.9985	1.5	5	-41.81	94.75	8.04	113.81	1.24	95.02	1	0.61
145	Ipconazole	5~100	y=70497.030535x-96788.415175	0.9991	1.5	5	-32.49	112.71	7.18	109.86	8.58	84.88	3.44	5.38
146	Isazofos	5~100	y=9577.562572x-15676.768367	0.9986	1.5	5	-65.73	86.74	3.39	81.13	8.07	115.27	11.42	3.45
147	Isocarbamid	10~100	y=6096.913360x-9821.560239	0.9984	3	10	-67.77	113.96	2.89	106.45	1.05	97.32	5.39	1.64
148	isocarbophos	5~100	y=1564.645903x-35453.564658	0.9894	1.5	5	-39.31	73.47	13.68	71.09	0.96	95.68	14.42	3.02

No.	Compounds	Linear range (μg/L)	Linearequation	R^2	LOD (μg/L)	LOQ (μg/L)	Matrix effect (%)	Recovery (LOQ)	RSD (LOQ)	Recovery (2×LOQ)	RSD (2×LOQ)	Recovery 10×LOQ	RSD (10×LOQ)	RSDwR (10×LOQ)
149	Isofenphos-Methyl	5~100	y=6283.081731x-5313.093471	0.9997	1.5	5	-23.65	103.32	7.29	79.23	0.67	81.31	1.24	5.18
150	Isomethiozin	10~100	y=1831.386038x-5937.391930	0.9975	3	10	-27.71	90.69	11.79	86.38	8.3	102.39	12.29	10.81
151	Isoprothiolane	5~100	y=13727.100014x-50549.024609	0.9986	1.5	5	-51.47	101.46	9.8	115.43	8.7	117.43	3.12	5.76
152	Isoproturon	5~100	y=89688.532952x-259674.914964	0.9973	1.5	5	-30.49	116.72	5.68	111.65	2.32	110.03	0.74	6.31
153	Isoxadifen-ethyl	5~100	y=11456.977771x-29579.560886	0.9985	1.5	5	-16.79	112.54	9.1	112.6	2.96	115.89	3.33	4.87
154	Karbutilate	10~100	y=6293.048193x-13935.729501	0.9981	3	10	-28.07	113.84	5.54	93.2	4.05	106.2	6.83	6.15
155	Mefenacet	5~100	y=66628.599608x-278067.341907	0.9989	1.5	5	-27.42	111	6.41	101.05	4.11	85.55	1.25	3.72
156	Mefenpyr-diethyl	10~100	y=30459.554965x-129874.773328	0.998	3	10	-11.64	115.68	3.06	105.82	4.8	96.19	2.06	2.62
157	Mepronil	5~100	y=79872.532261x-312636.862142	0.9989	1.5	5	-28.9	118.98	8.81	116.32	2.62	109.64	0.91	5.48
158	Metaflumizone-(E)	20~100	y=2148.962840x-4294.862941	0.9935	6	20	-35.96	85.37	5.03	98.55	8.4	93.4	3.42	1.25
159	Metaflumizone-(Z)	20~100	y=4294.017482x+2018.386501	0.9928	6	20	-38.12	77.84	5.29	86.3	9.42	85.29	6.93	9.66
160	Metalaxyl	10~100	y=2130.849207x-1394.073912	0.9983	3	10	-22.44	108.14	4.43	94.28	2.49	102.49	6.3	4.03
161	Metamitron	5~100	y=17912.547555x-12353.188855	0.9988	1.5	5	-45.56	116.07	13.07	118.33	14.6	94.82	1.33	8.69
162	Metazachlor	5~100	y=12639.448645x-44031.828550	0.9976	1.5	5	-23.16	117.53	17.7	116.58	9.63	114.79	2.38	0.47
163	Metconazole	5~100	y=49017.314859x+23891.490321	0.9958	1.5	5	-47.9	73.96	7.69	106.92	2.85	81.95	1.84	0.64
164	Methamidophos	5~100	y=20346.445742x-2657.678541	0.9997	1.5	5	-26.4	95.22	7.44	80.14	7.28	110.76	13.28	10.18
165	Methfuroxam	5~100	y=6283.074821x-21073.964286	0.9971	1.5	5	-29.39	108.04	13.94	97.59	18.49	72.74	9.66	4.41
166	Methoprotryne	5~100	y=206680.044163x-935116.113717	0.9979	1.5	5	-33.95	83.15	12.51	80.4	3.85	71.36	1	5.01
167	Metobromuron	5~100	y=5225.160334x-9191.857657	0.9979	1.5	5	-44.96	89.18	15.53	117.22	2.37	100.56	2.34	9.77
168	Metribuzin	5~100	y=26327.079185x-70083.161528	0.9942	1.5	5	-76.75	105.62	12.74	111.38	11.07	110.7	7.97	4.20
169	Metsulfuron-Methyl	10~100	y=17082.043063x-48743.208238	0.9973	3	10	116.58	110.7	6.51	92.94	3.71	96.04	10.59	2.09
170	Mevinphos-(E)	20~100	y=2356.643148x+846.478371	0.9932	6	20	-56.16	98.34	6.03	84.05	3.58	90.42	5.39	0.35

No.	Compounds	Linear range (μg/L)	Linearequation	R^2	LOD (μg/L)	LOQ (μg/L)	Matrix effect (%)	Recovery (LOQ)	RSD (LOQ)	Recovery (2×LOQ)	RSD (2×LOQ)	Recovery 10×LOQ	RSD (10×LOQ)	RSDwR (10×LOQ)
171	Mevinphos-(Z)	20~100	y=71096.808528x-451691.487098	0.9872	6	20	-40.24	88.31	8.23	85.03	6.01	98.4	8.14	0.35
172	Monocrotophos	5~100	y=12485.345451x-1578.787468	0.9936	1.5	5	-16.07	82.8	3.31	75.91	19.83	101.2	19.14	10.72
173	Monolinuron	10~100	y=4568.191793x-3113.538790	0.9965	3	10	-58.74	112.39	12.21	114.85	10.84	104.38	7.49	7.15
174	Monuron	5~100	y=34112.471834x-93295.002636	0.9984	1.5	5	-41.92	115.07	8.3	112.52	6.52	102.9	1.01	6.51
175	Myclobutanil	10~100	y=17688.419992x-55824.164759	0.9996	3	10	-17.57	113.31	4.34	107.51	2.65	105.39	5.22	4.90
176	Neburon	10~100	y=56810.293635x-237944.831141	0.9987	3	10	-13.58	114.82	9.16	107.95	8.39	98.41	11.42	6.81
177	Norflurazon	5~100	y=120255.057960x-448563.056361	0.9984	1.5	5	-38.74	95.49	6.33	105.3	2.58	86.5	1.52	7.12
178	Noruron	10~100	y=164324.092543x-652598.896671	0.9985	3	10	-24.81	113.14	4.67	93.29	6.28	103.18	5.83	2.78
179	Novaluron	10~100	y=391.689516x+793.461583	0.9933	3	10	-36.72	114.34	15.49	103.38	7.05	94.29	12.06	10.67
180	Nuarimol	5~100	y=23186.542343x-22073.358091	0.9969	1.5	5	-65.06	78.66	4.21	118.23	3.58	117.62	1.66	6.52
181	Ofurace	5~100	y=35083.124784x-68682.742719	0.9985	1.5	5	-33.69	118.52	7.98	119.86	4.33	117.63	1.59	7.64
182	Orbencarb	10~100	y=5936.577144x+9561.517443	0.9954	3	10	-34.37	114.31	7.99	106.42	2.84	102.48	5.93	4.17
183	oxadiazon	5~100	y=740.133224x-1031.996081	0.9873	1.5	5	-60.33	108.05	19.23	99.08	9.75	119.07	5.19	6.71
184	Oxadixyl	5~100	y=20197.962177x-64265.540081	0.9985	1.5	5	-25.75	114.98	16.28	106.64	12.09	109.69	2.7	8.69
185	Oxasulfuron	5~100	y=9797.447204x-20939.185758	0.9976	1.5	5	7.61	94	11.64	114.02	12.89	75.11	6.02	4.75
186	Oxycarboxin	5~100	y=10260.130856x-8224.098090	0.9982	1.5	5	-35.67	111.67	17.94	110.87	10.12	119.21	3.52	4.54
187	oxydemeton-methyl	10~100	y=13937.075721x-6890.752904	0.9982	3	10	-10.81	82.28	10.93	94.6	5.81	102.58	7.03	6.53
188	Pebulate	10~100	y=5002.402089x-17972.379197	0.9995	3	10	-11.32	90.87	12.59	105.36	9.03	102.3	5.59	2.84
189	Penconazole	5~100	y=23081.783510x-63927.073661	0.9959	1.5	5	-40.31	98.86	6.2	112.49	1.96	102.96	1.08	0.82
190	Penflufen	10~100	y=132312.044985x-571162.555820	0.9982	3	10	-22.84	118.02	4.02	95.38	3.85	106.53	6.04	5.27
191	Penoxsulam	5~100	y=102109.897032x-439663.436358	0.9989	1.5	5	94.79	112.96	13.31	83.17	6.96	78.72	1.31	4.79
192	Pentanochlor	5~100	y=99633.671782x-652492.749169	0.9963	1.5	5	-24	119.24	6.95	116.74	4.41	104.14	1	4.73

No.	Compounds	Linear range (μg/L)	Linearequation	R^2	LOD (μg/L)	LOQ (μg/L)	Matrix effect (%)	Recovery (LOQ)	RSD (LOQ)	Recovery (2×LOQ)	RSD (2×LOQ)	Recovery 10×LOQ	RSD (10×LOQ)	RSDwR (10×LOQ)
193	Pethoxamid	5~100	y=47937.721670x-251843.675082	0.9992	1.5	5	-45.5	113.81	8.37	110.59	2.36	117.61	1.72	6.00
194	Phorate-oxon-sulfone	10~100	y=5235.456452x-24574.468243	0.9938	3	10	-45.37	117.19	17.17	107.29	12.04	96.95	9.48	9.63
195	Phorate-oxon-sulfoxide	5~100	y=25657.565724x-52435.7893562	0.9978	1.5	5	-33.33	100.19	12.44	115.26	14.03	105.99	7.78	6.18
196	Phorate-Sulfone	5~100	y=36575.097136x-2577.785676	0.9946	1.5	5	-21.81	81.95	18.38	73.13	4.09	87.05	0.46	7.81
197	Phorate-Sulfoxide	5~100	y=5676.768864x+2786.768453	0.9898	1.5	5	-67.9	108.01	11.09	103.98	13.58	115.21	5.52	0.48
198	Phosfolan	5~100	y=63928.378193x-5937.319044	0.9956	1.5	5	-72.63	117.73	13.69	81.42	10.88	86.73	11.18	5.71
199	Phosfolan-Methyl	5~100	y=34294.481038x-7391.301783	0.9995	1.5	5	108.16	79.78	14.91	100.33	6.96	108.93	4.35	2.84
200	Phosphamidon-(E)	10~100	y=54879.888313x-167616.630205	0.9988	3	10	-23.65	96.34	10.95	106.94	8.29	101.26	7.59	9.60
201	Phosphamidon-(Z)	10~100	y=24954.781782x-79237.033535	0.9971	3	10	-27.71	90.3	7.05	100.49	9.34	102.72	5.11	9.33
202	Phoxim	5~100	y=24352.889462x-45671.568256	0.9994	1.5	5	-32.1	106.65	8.07	98.29	2.18	84.58	1.37	5.32
203	Piperonyl Butoxide	10~100	y=93465.134866x-86793.678442	0.9945	3	10	-40.78	117.73	6.67	107.26	4.71	96.28	5.05	4.90
204	Promecarb	10~100	y=31038.964829x-4920.417935	0.9956	3	10	-63.29	76.11	19.1	93.01	14.04	85.49	15.2	2.69
205	Prometryn	10~100	y=5346.657683x-56673.676835	0.9957	3	10	-97.73	119.54	7.57	108.55	5.94	92.4	3.06	7.46
206	Propachlor	10~100	y=76823.284918x-29381.037193	0.9993	3	10	-31.25	114.15	3.66	110.59	4.72	102.59	6.49	1.58
207	Propazine	5~100	y=123416.429954x-311319.663729	0.9966	1.5	5	-38.81	79.82	8.24	115.85	7.76	111.91	18.15	16.44
208	Propisochlor	10~100	y=6863.981041x+7118.434448	0.9981	3	10	-26.27	113.46	4.38	105.39	6.92	92.5	5.64	8.47
209	Propyzamide	5~100	y=3309.484272x-2689.750749	0.9953	1.5	5	-27.71	119.39	11.31	115.29	9.12	119.73	2.9	5.27
210	Prosulfocarb	5~100	y=47042.542790x-188520.658876	0.9984	1.5	5	-21.65	112.36	16.43	115.81	6.29	110.76	6.71	7.15
211	Prosulfuron	10~100	y=2668.247898x+460.500231	0.9957	3	10	-15.84	77.19	12.49	90.05	8.62	83.52	10.92	3.75
212	Prothiofos	10~100	y=16318.308581x-10310.376108	0.9983	3	10	-33.57	91.82	18.83	105.39	15.06	84.82	5.05	9.46
213	Prothoate	10~100	y=52889.160984x-191963.243929	0.9992	3	10	-25.76	115.94	3.63	108.94	2.84	96.35	8.05	9.83
214	Pyracarbolid	5~100	y=73684.180754x-272532.350757	0.9983	1.5	5	-37.56	118.91	9.02	116.8	3.96	107.85	1	3.05

No.	Compounds	Linear range (μg/L)	Linearequation	R^2	LOD (μg/L)	LOQ (μg/L)	Matrix effect (%)	Recovery (LOQ)	RSD (LOQ)	Recovery (2×LOQ)	RSD (2×LOQ)	Recovery 10×LOQ	RSD (10×LOQ)	RSDwR (10×LOQ)
215	Pyraclostrobin	5~100	y=34664.565724x-46573.678351	0.9964	1.5	5	-26.08	78.9	14.28	87.86	8.92	78.81	2.68	8.18
216	Pyraflufen-ethyl	10~100	y=40965.806647x-62809.597623	0.9961	3	10	42.2	117.32	2.25	107.27	5.81	96.56	10.84	9.07
217	Pyrametostrobin	5~100	y=67428.777999x-280849.655057	0.9987	1.5	5	-32.52	97.42	9.85	90.5	2.99	75.05	2.14	3.52
218	Pyrazosulfuron-ethyl	5~100	y=10174.690216x-12840.037918	0.9972	1.5	5	-35.55	72.89	11.06	79.44	5.61	70.97	2.63	7.75
219	Pyrazoxyfen	5~100	y=64793.017112x-232467.021371	0.9994	1.5	5	-9.95	111.71	7.09	102.62	8.87	92.76	1.89	0.20
220	Pyributicarb	10~100	y=127270.238952x-623104.921764	0.9986	3	10	-27.06	117.19	5.85	109.32	3.94	94.29	8.29	4.70
221	Pyridaphenthion	10~100	y=65801.875648x-238534.221046	0.9991	3	10	-19.36	117.19	5.85	104.89	4.71	97.3	6.83	0.68
222	Pyriftalid	5~100	y=181626.753860x-1170782.000742	0.9959	1.5	5	-40.13	109.57	8.59	87.98	3.35	70.76	0.82	1.08
223	Pyrimitate	5~100	y=181733.324801x-990149.271336	0.9986	1.5	5	-20.58	113.11	10.94	109	3.49	92.75	1.61	8.86
224	Pyriminobac-Methyl-(E)	5~100	y=1914.675717x+1624.664340	0.9942	1.5	5	108.16	72.38	12.38	79.3	6.37	83.91	5.79	3.08
225	Pyriminobac-Methyl-(Z)	5~100	y=36516.778246x-4677.857366	0.9997	1.5	5	-32.28	75.3	15.37	80.37	9.36	95.28	10.42	6.30
226	Pyriproxyfen	5~100	y=112622.037423x-613024.950299	0.999	1.5	5	-43.99	77.18	6.62	116.17	2.88	86.51	1.12	2.25
227	Pyrisoxazole	5~100	y=45208.481073x-83721.038425	0.999	1.5	5	-37.1	71.64	15.88	99.31	8.61	92.86	3.5	2.18
228	Pyroquilon	5~100	y=70586.126395x-217845.680549	0.9983	1.5	5	-46.97	112.07	10.39	117.35	4.79	105.29	0.95	1.72
229	Sebuthylazine-desethyl	5~100	y=12435.175420x-9666.876654	0.9993	1.5	5	-79.4	114.37	15.64	107.99	7.72	104.76	5.63	0.67
230	Sedaxane	5~100	y=8911.096653x-10238.289494	0.996	1.5	5	-39.28	113.92	15.29	85.31	18.73	82.08	5.48	6.25
231	Siduron	10~100	y=29622.120519x-71876.471003	0.9986	3	10	-18.65	117.23	5.1	103.57	8.05	98.31	6.99	4.89
232	Silthiofam	10~100	y=43420.934597x-170824.921526	0.9988	3	10	-18.73	111.23	2.72	95.3	5.39	103.93	6.43	3.72
233	Sulfometuron-Methyl	10~100	y=21355.700412x-68120.304107	0.9993	3	10	-0.29	118.72	3.17	93.49	4.71	104.29	4.2	4.17
234	Sulfotep	5~100	y=5762.217841x-20690.925205	0.9986	1.5	5	-13.31	92.35	8.96	106.09	9.98	115.55	15.33	10.48

No.	Compounds	Linear range (μg/L)	Linearequation	R^2	LOD（μg/L）	LOQ（μg/L）	Matrix effect (%)	Recovery (LOQ)	RSD (LOQ）	Recovery (2×LOQ)	RSD (2×LOQ)	Recovery 10×LOQ	RSD (10×LOQ)	RSDwR (10×LOQ)
235	Sulprofos	5~100	y=91536.637401x-203342.257741	0.9986	1.5	5	2.85	119.84	12.65	119.26	1.48	103.65	3.32	0.73
236	Tebuconazole	10~100	y=39039.976326x-137306.948666	0.9974	3	10	-23.08	117.13	6.88	102.5	5.72	98.32	9.53	9.72
237	Tebufenpyrad	10~100	y=86599.881477x-338591.334060	0.9979	3	10	-7.38	90.17	2.16	104.29	4.75	103.94	7.44	1.31
238	Tebupirimfos	5~100	y=23904.947103x-52091.489103	0.9982	1.5	5	-13.86	97.76	15.89	119.93	6.14	102.75	5.7	5.08
239	Tebutam	5~100	y=86599.881477x-338591.334060	0.9979	1.5	5	-48.71	110.49	10.45	112.13	3.69	116.69	1.34	3.19
240	Tebuthiuron	5~100	y=58751.980923x-229015.424491	0.9982	1.5	5	-57.69	113.62	5.75	114.29	3.35	107.55	0.95	5.09
241	Temephos	5~100	y=21794.532963x-34865.599777	0.9969	1.5	5	-28.08	110.21	6.12	110.43	2.89	87	2.34	7.12
242	Tepraloxydim	10~100	y=2179.060795x+5487.889404	0.9909	3	10	-68.83	76.78	5.47	84.28	7.39	110.39	3.59	7.85
243	Terbucarb	10~100	y=2799.367974x+456763.236717	0.9906	3	10	1.12	116.66	5.96	106.29	7.94	102.74	6.02	8.64
244	Terbufos	5~100	y=14241.763091x-21383.310974	0.9956	1.5	5	-34.51	80.03	15.37	103.64	16.24	80.15	16.49	8.72
245	Terbufos-Sulfone	5~100	y=8685.577424x-66765.797861	0.9917	1.5	5	-44.52	80.83	18.35	115.6	0.45	79.91	11.79	7.58
246	Terbufos-Sulfoxide	5~100	y=9735.567145x-4566.782564	0.9956	1.5	5	-28.81	116.47	0.23	102.79	3.11	108.12	11.02	6.08
247	Terbumeton	10~100	y=162473.239294x-289889.265455	0.9919	3	10	-32.66	115.45	3.84	98.4	4.29	103.85	9.03	7.16
248	Terbuthylazine	5~100	y=50174.785891x+44712.145421	0.9894	1.5	5	-48.91	107.74	12.97	93.14	10.37	73.62	10.5	9.98
249	Tetraconazole	5~100	y=32803.232307x-67777.335925	0.9982	1.5	5	-57.82	119.99	4.5	114.05	1.76	115	0.96	0.07
250	Thenylchlor	5~100	y=2561.856022x-2540.285976	0.9999	1.5	5	-23.61	90.67	6.96	113.63	7.4	114.15	2.24	8.25
251	Thiazafluron	5~100	y=3759.709746x-6137.963462	0.998	1.5	5	-58.88	113.57	11.39	113.57	7.73	109.38	2.5	2.16
252	Thiazopyr	10~100	y=135452.377502x-560569.054638	0.999	3	10	-4.09	82.51	5.35	96.21	7.05	105.29	9.22	4.11
253	Thiobencarb	50~200	y=6008.657561x+7312.453189	0.9944	15	50	-32.91	114.34	3.14	104.48	7.29	102.96	5.69	7.16
254	Thiodicarb	10~100	y=13396.101346x-32488.150171	0.9977	3	10	3.02	119.13	3.88	105.92	5.29	96.39	4.92	2.39
255	Thionazin	10~100	y=8516.068240x-45942.487963	0.9946	3	10	-10.37	90.91	14.37	109.84	11.08	96.24	4.88	4.19
256	Tiocarbazil	10~100	y=55112.493331x-189919.756025	0.9975	3	10	-53.86	114.35	11.99	102.89	7.93	95.44	9.3	4.33

No.	Compounds	Linear range (μg/L)	Linearequation	R^2	LOD (μg/L)	LOQ (μg/L)	Matrix effect (%)	Recovery (LOQ)	RSD (LOQ)	Recovery (2×LOQ)	RSD (2×LOQ)	Recovery 10×LOQ	RSD (10×LOQ)	RSDwR (10×LOQ)
257	Tolclofos-methyl	5~100	y=1110.860972x-3063.989850	0.9985	1.5	5	-25.49	92.06	13.89	97.67	15.73	99.94	3.92	4.72
258	Tolfenpyrad	5~100	y=22153.849012x-78253.856012	0.9989	1.5	5	-29.71	111.28	9.66	98.18	3.95	71.45	1.49	6.53
259	Triadimefon	10~100	y=17343.454213x-3454.798466	0.9983	3	10	-69.5	117.51	8.59	103.19	5.82	98.5	7.58	4.39
260	Triadimenol	5~100	y=12452.456424x-54657.676835	0.9975	1.5	5	-25.72	102.56	18.72	114.08	4.69	108.54	0.95	6.28
261	Triamiphos	5~100	y=156680.558453x-727554.461231	0.9979	1.5	5	-32.78	117.92	5.67	103.92	3.96	94.76	0.96	3.24
262	Triasulfuron	5~100	y=30840.456553x-101780.509831	0.9988	1.5	5	133.77	113.06	9.08	100.81	5.49	85.66	1.3	5.98
263	Triazophos	50~200	y=34513.774625x-62772.657714	0.9948	15	50	120.72	101.86	11.83	96.59	8.03	98.2	3.31	4.17
264	Tribufos	5~100	y=103568.209004x-444293.470082	0.9978	1.5	5	-36.16	119.41	6.38	115.18	2.27	103.19	1.58	0.72
265	Trietazine	5~100	y=81014.760906x-267854.726955	0.9984	1.5	5	-50.46	98.4	7.55	114.78	1.56	89.44	1.14	6.86
266	Trifloxystrobin	10~100	y=120050.198334x-473056.931513	0.999	3	10	-25.51	92.77	17.58	98.05	7.08	85.29	12.47	3.71
267	Triflumizole	5~100	y=21320.773415x-67537.232157	0.9993	1.5	5	-30.79	117.68	10.94	111.21	3.93	98.78	2.12	0.71
268	Triticonazole	5~100	y=31943.214989x-13638.986659	0.9997	1.5	5	-43.58	117.88	13.87	115.01	12.89	91.28	6.37	9.86
269	Uniconazole	5~100	y=33648.984759x-44084.033905	0.9967	1.5	5	-66.34	86.01	5.5	112.08	2.41	95.2	1.26	2.05
270	Zoxamide	10~100	y=28777.356287x-61448.316919	0.9985	3	10	-0.63	118.45	17.53	109.04	7.88	108.02	2.79	4.83

Table S3. Methodological validation of 270 pesticides and two Q-markers in *AS* piece

No.	Compounds	Linear range （μg/kg）	Linearequation	R^2	LOQ (μg/kg）	Matrix effect (%)	Recovery (LOQ)	RSD (LOQ)	Recovery (2×LOQ)	RSD (2×LOQ)	Recovery (10×LOQ)	RSD (10×LOQ)	RSDwR (10×LOQ)
1	1-naphthyl acetamide	10~200	y=355.702983x-3336.195741	0.9819	20	-57.15	113.05	3.95	98.88	10.15	103.67	8.66	4.41
2	2,6-Dichlorobenzamide	10~200	y=1556.078396x+38281.075746	0.9819	30	-52.04	106.24	3.98	84.43	9.65	96.45	7.83	9.78
3	3,4,5-Trimethacarb	10~200	y=2368.585306x+14007.564331	0.9837	10	61.85	95.2	8.54	116.82	7.31	102.35	3.26	1.15
4	Acetamiprid-(E)	10~200	y=658.031436x+3453.901437	0.9990	10	-45.99	91.33	10.29	107.09	9.67	111.94	3.38	8.87
5	Acetamiprid-(Z)	10~200	y=692.840259x-10248.108493	0.9833	10	-45.61	92.05	12.12	92.25	3.42	108.24	3.49	6.97
6	Aldicarb	20~200	y=2604.250491x-6023.190492	0.9948	20	-59.33	70.82	7.5	84.27	11.57	92.06	11.78	6.06
7	Aldicarb-sulfone	10~200	y=1943.842039x-2084.610938	0.9986	10	-50.67	90.41	14.14	83.78	11.42	82.78	8.61	7.22
8	Aldicarb-sulfoxide	10~200	y=29157.112933x-45227.936455	0.9970	10	-58.94	103.05	13.47	111.35	3.47	72.17	9.73	9.39
9	Ametryn	10~200	y=1348.869969x-1247.423446	0.9980	10	-55.83	102.35	5.01	111.04	9.03	110.5	5.19	4.96
10	Aminocarb	10~200	y=16110.133359x-23254.461744	0.9976	10	-44.55	75.17	9.5	77.38	5.27	91.84	3.68	3.63
11	Ancymidol	10~200	y=5871.282047x+53582.750081	0.9990	10	-53.9	77.62	9.17	101.19	2.53	110.39	2.15	3.90
12	Anilofos	10~200	y=5292.097936x-9269.876729	0.9978	10	-59.55	104.36	2.5	109.72	5.61	103.51	3.16	1.36
13	Aramite	20~200	y=42.535742x+1542.846926	0.9845	20	-23.23	98.23	5.21	103.31	7.19	97.63	4.08	2.75
14	Atraton	10~200	y=115.872477x-861.157934	0.9974	10	-54.27	116.15	19.24	92.22	19.76	113.68	13.31	8.06
15	Atrazine-desisopropyl	10~200	y=4348.895602x-7316.547618	0.9964	10	-55.83	113.01	6.68	113.77	6.86	115.69	4.62	3.04
16	Azaconazole	10~200	y=321.925046x+2270.472982	0.9876	10	-43.54	89.62	5.27	108.37	7.26	113.3	3.75	5.23

No.	Compounds	Linear range （μg/kg）	Linearequation	R^2	LOQ (μg/kg）	Matrix effect (%)	Recovery (LOQ)	RSD (LOQ)	Recovery (2×LOQ)	RSD (2×LOQ)	Recovery (10×LOQ)	RSD (10×LOQ)	RSDwR (10×LOQ)
17	azadirachtin	10~200	y=2332.755144x-2148.272619	0.9928	10	-57.21	74.06	17.43	113.64	13.27	102.48	13.76	10.07
18	Aziprotryne	20~200	y=2216.161830x-9420.070501	0.9950	20	-60.77	97.58	15.9	98.11	10.21	106.62	10.67	6.61
19	Azoxystrobin-(E)	10~200	y=1005.465449x+11390.440779	0.9827	10	-62.42	96.6	4.25	105.73	4.08	101.73	3.29	7.65
20	Azoxystrobin-(Z)	10~200	y=7576.503804x-11136.858880	0.9968	10	-59.03	97.27	5.19	110.48	6.63	115.89	5.36	0.33
21	Beflubutamid	10~200	y=1545.895333x+28895.235612	0.9924	10	-60.03	88.85	15.27	115.91	8.65	110.25	6.72	1.62
22	Benalaxyl	10~200	y=4128.123469x-2408.631607	0.9954	10	-61.28	108.93	11.56	116.47	12.24	110.01	4.35	1.77
23	Bendiocarb	10~200	y=245.646136x-2461.435725	0.9982	10	-16.35	89.64	5.90	99.46	9.04	105.32	9.03	6.07
24	Benodanil	10~200	y=120.083387x+314.284177	0.9859	10	-60.77	95.99	6.88	117.93	5.8	112	6.4	2.12
25	Bensulide	20~200	y=4054.632868x-4720.817021	0.9960	20	-57.88	86.12	15.87	114.81	9.15	99.03	7.89	6.34
26	Benzovindiflupyr	10~200	y=3796.775486x-1195.418872	0.9986	10	-60.72	107.13	4.77	109.87	5.62	117.73	2.73	4.68
27	Bitertanol	10~200	y=1079.941234x-2306.311596	0.9974	10	-60.27	115.01	17.52	95.33	13.95	109.61	3.7	1.80
28	Blasticidin-S	10~200	y=595.796187x+1233.794580	0.9837	10	-62.25	110.54	4.59	115.87	4.67	117.28	6.04	7.62
29	Boscalid	10~200	y=2065.454638x+1276.821812	0.9976	10	-53.52	109.43	6.31	103.13	2.76	114.97	2.41	3.38
30	Bromacil	20~200	y=100.459113x+286.882138	0.9944	20	-62.22	114.86	12.67	107.18	5.32	83.17	9.47	4.28
31	Bromfenvinfos-Methyl	10~200	y=644.948337x-702.208761	0.9942	10	-55.87	112.29	5.47	115.16	8.56	110.29	5.49	5.48
32	Bromobutide	10~200	y=742.777687x+6890.108771	0.9876	10	-62.69	95.22	9.24	103.96	11.07	116.05	7.09	9.01
33	Bromuconazole	10~200	y=850.224364x-2233.861880	0.9962	10	-59.35	88.51	7.52	101.2	10.35	95.52	4.7	0.64
34	Bupirimate	10~200	y=370.884350x-2341.966048	0.9978	10	-62.07	91	8.39	73.56	3.16	97.61	1.82	7.92
35	Butafenacil	10~200	y=14781.776546x+83652.549172	0.9958	10	-58.56	107.97	6.4	109.04	4.48	98.48	3.87	7.16
36	Butamifos	10~200	y=2865.905055x-9077.208364	0.9970	10	-61.71	98.51	18.75	112.37	4.85	99.52	3.06	3.32

No.	Compounds	Linear range （μg/kg）	Linearequation	R^2	LOQ (μg/kg）	Matrix effect (%)	Recovery (LOQ)	RSD (LOQ)	Recovery (2×LOQ)	RSD (2×LOQ)	Recovery (10×LOQ)	RSD (10×LOQ)	RSDwR (10×LOQ)
37	Cadusafos	20~200	y=2194.592103x-2391.289517	0.9978	20	-58.61	114.68	4.83	76.04	13.32	97.28	10.47	9.57
38	Cafenstrole	10~200	y=1699.440421x+29143.728275	0.9849	10	-42.73	93.83	13.78	102.74	14.8	117.62	3.01	7.46
39	Carbendazim	10~200	y=4645.660968x-56.953576	0.9934	10	-62.44	119.94	9.31	109.75	4.98	114.29	6.03	9.83
40	Carbofuran	20~200	y=2810.391034x-210.390419	0.9958	20	-64.67	106.35	12.68	86.49	14.41	83.07	15.78	13.06
41	Carbofuran-3-Hydroxy	20~200	y=1313.290315x-1214.257159	0.9974	20	-52.67	76.68	8.97	79.77	5.66	107.47	6.92	3.49
42	Carfentrazone-ethyl	10~200	y=4837.715712x+14422.636851	0.9944	10	-63.24	83.47	12.36	111.24	10.76	115.71	5.34	4.05
43	Carpropamid	10~200	y=1242.535216x-12451.535164	0.9916	10	-25.34	82.42	9.24	89.45	2.46	101.74	6.28	2.82
44	Chlordimeform	10~200	y=1452.524674x-6462.646837	0.9926	10	-38.53	93.42	7.94	88.46	6.4	90.62	7.45	8.47
45	Chlorfenvinphos-(E)	10~200	y=256.028315x-1061.523135	0.9998	10	-53.45	103.14	9.14	106.94	4.97	115.99	2.93	5.07
46	Chlorfenvinphos-(Z)	10~200	y=1103.845715x-1197.574008	0.9974	10	-62.44	106.81	9.31	109.75	4.98	114.29	6.03	2.43
47	Chloridazon	10~200	y=65.986543x-725.830330	0.9920	10	-62.14	85.2	2.95	102.21	3.86	104.26	16.13	9.66
48	Chlorimuron-ethyl	10~200	y=4595.578496x+35325.549745	0.9857	10	-40.11	90.17	7.49	100.91	5.73	109.47	4	9.75
49	Chlorpyrifos	10~200	y=2048.972409x-3166.952039	0.9974	10	-59.37	0	0	75.63	14.74	82.71	15.92	10.95
50	Chlorsulfuron	10~200	y=362.736836x-3825.625866	0.9884	10	-30.92	85.39	6.49	90.59	6.35	98.49	4.92	0.64
51	Clodinafop-propargyl	10~200	y=2729.492836x-2387.765196	0.9974	10	-63.16	92.58	9.26	106.71	7.19	112.03	4.55	7.24
52	Clomazone	10~200	y=1150.121497x+350.582990	0.9974	10	-51.22	79.86	14.46	106.64	16.2	103.77	8.22	7.55
53	Cloransulam-methyl	10~200	y=3360.367440x-202.830616	0.9954	10	-31.32	92.43	6.06	103.74	6.11	111.63	3.24	3.05
54	Crufomate	10~200	y=223.368883x-104.303156	0.9930	10	-51.41	100.38	4.96	108.72	4.67	114.36	1.9	4.67
55	Cyanazine	10~200	y=4241.607173x-7930.011219	0.9982	10	-49.11	112.82	6.24	102.94	9.34	99.97	4.46	0.94
56	Cyazofamid	10~200	y=4245.942941x-1751.615728	0.9930	10	-62.64	110.1	11.97	90.72	12.02	118.5	7.45	4.82
57	Cycloate	10~200	y=2884.263794x-3055.531068	0.9984	10	-52.73	91.87	11.6	95.23	9.79	107	4.33	3.50

No.	Compounds	Linear range （μg/kg）	Linearequation	R^2	LOQ (μg/kg)	Matrix effect (%)	Recovery (LOQ)	RSD (LOQ)	Recovery (2×LOQ)	RSD (2×LOQ)	Recovery (10×LOQ)	RSD (10×LOQ)	RSDwR (10×LOQ)
58	Cycluron	10~200	y=22082.864701x-53513.008362	0.9984	10	-60.1	104.56	8.06	107.06	3.98	96.92	4.21	8.66
59	Cyenopyrafen	10~200	y=4244.404437x+92908.158758	0.9876	10	-63.45	102.4	9.27	110.31	2.99	105.16	3.9	9.76
60	Cyflufenamid	10~200	y=353.051067x+433.450022	0.9968	10	-64.31	90.55	14.42	114.46	8.95	118.19	3.3	9.32
61	Cymiazole	10~200	y=6854.334486x+9220.222168	0.9974	10	-64.44	92.33	19.37	117.78	6.09	113.09	3.34	6.23
62	Cymoxanil	10~200	y=11971.550252x+40128.809346	0.9990	10	-58.66	89.73	13.08	77.57	9.26	101.46	8.57	8.00
63	Cyprazine	10~200	y=959.934133x+2581.075544	0.9966	10	-54.63	90.54	8.56	107.02	4.41	118.74	4.47	5.37
64	Cyproconazole	10~200	y=6096.679247x+18842.925496	0.9956	10	-55.02	97.62	13.75	96.45	9.69	74.03	9.93	9.30
65	Cyprofuram	10~200	y=2883.048433x+869.786650	0.9948	10	-58.63	114.37	15.19	113.15	6.66	116.34	1.84	6.20
66	Daimuron	10~200	y=17487.009929x-16661.257216	0.9974	10	-49.95	91.56	9.07	107.85	6.95	115.02	3.73	8.40
67	demeton	20~200	y=2913.832052x-420.319048	0.9956	20	-51.33	97.57	7.27	94.46	3.46	105.92	7.11	4.84
68	Desethylterbuthylazine	10~200	y=63823.213328x-95168.245194	0.9984	10	-58.33	87.46	7.46	97.23	2.58	93.37	5.52	1.37
69	Desmetryn	10~200	y=2965.809075x+54991.833871	0.9916	10	-53.24	81.02	5.52	86.73	6.04	90.93	3.23	4.78
70	Diazinon	10~200	y=4441.433102x-2182.752635	0.9970	10	-64.09	100.8	8.52	104.94	14.85	102.31	9.61	2.79
71	Dichlormid	10~200	y=2676.650813x-3428.055101	0.9984	10	-65.27	86.31	7.04	78.84	6.84	73.73	3.78	3.58
72	Diclocymet	10~200	y=2368.565331x+3258.766471	0.9984	10	-63.99	97.82	9.35	105.94	5.57	110.17	5.85	0.29
73	Diclosulam	10~200	y=24060.853901x+130992.976099	0.9974	10	-28.97	90.42	3.94	98.68	5.78	110	1.82	0.82
74	Diethyltoluamide	10~200	y=3627.325280x+12262.853584	0.9978	10	-52.08	102.63	8.25	111.44	7.41	116.52	3.7	6.40

No.	Compounds	Linear range （μg/kg）	Linearequation	R^2	LOQ (μg/kg)	Matrix effect (%)	Recovery (LOQ)	RSD (LOQ)	Recovery (2×LOQ)	RSD (2×LOQ)	Recovery (10×LOQ)	RSD (10×LOQ)	RSDwR (10×LOQ)
75	Difenoconazole	10~200	y=25186.720617x-2398.637614	0.9978	10	-61.72	106.86	6.43	111.74	3.7	113.71	2.35	1.50
76	Difenoxuron	10~200	y=493.180695x+233.502074	0.9990	10	-53.24	100.32	7.57	107.23	4.36	101.42	3.96	6.34
77	Dimethachlor	10~200	y=6241.465903x-7487.470690	0.9988	10	-57.67	113.2	7.79	110.98	9.7	106.89	5.39	4.63
78	Dimethenamid	10~200	y=56531.752153x-116250.121117	0.9968	10	-60.98	105.72	5.49	95.34	3.7	102.54	7.21	2.92
79	Dimethomorph	10~200	y=3152.537133x-12641.672908	0.9976	10	-52.06	102	9.55	114.24	7.91	109.92	5.61	8.45
80	Dimethylvinphos (E)	10~200	y=6502.033619x+14302.709070	0.9988	10	-62.25	72.27	17.19	91.4	15.01	106.72	8.08	9.46
81	Dimethylvinphos (Z)	10~200	y=120.990340x-317.486852	0.9986	10	-57.59	107.93	11.25	104.93	6.25	104.02	2.17	2.34
82	Dimetilan	10~200	y=989.780032x-1334.714265	0.9946	10	-54.91	100.63	11.38	116.08	6.52	112.78	7.01	0.90
83	Diphenamid	10~200	y=10884.408370x-6795.593900	0.9988	10	-47.46	103.73	5.5	112.57	3.79	106.22	3.67	6.67
84	Dipropetryn	10~200	y=-124.661070x+64341.360853	0.9807	20	-58.9	100.66	5.46	109.71	4.78	95.87	11.94	7.00
85	Dipropyl Isocinchomeronate	10~200	y=323.453585x+746.835754	0.9805	30	-31.31	78.9	4.9	89.59	4.89	99.05	8.09	5.55
86	Dithiopyr	10~200	y=46899.293724x-50683.132388	0.9982	10	-54.05	84.74	2.04	90.92	3.93	103.85	0.85	3.54
87	Esprocarb	10~200	y=15990.049607x-30980.224125	0.9982	10	-66.59	89.65	3.27	102.42	5.04	93.93	2.73	1.34
88	Etaconazole	10~200	y=7074.638779x-14520.982701	0.9992	10	-81.72	81.47	8.72	92.44	19.6	85.81	7.62	6.74
89	Ethiofencarb-sulfone	10~200	y=3770.925907x-4221.119440	0.9964	10	-17.27	115.26	6.57	114.19	4.5	113.06	3.34	9.49
90	Ethiofencarb-sulfoxide	10~200	y=3777.201922x-3931.250847	0.9970	10	-47.29	79.36	10.42	107.62	6.17	98	5.91	3.43
91	Ethoprophos	20~200	y=7793.253826x+3057.949190	0.9964	20	-46.41	103.55	6.97	84.78	14.45	93.37	10.76	8.94

No.	Compounds	Linear range （μg/kg）	Linearequation	R^2	LOQ (μg/kg)	Matrix effect (%)	Recovery (LOQ)	RSD (LOQ)	Recovery (2×LOQ)	RSD (2×LOQ)	Recovery (10×LOQ)	RSD (10×LOQ)	RSDwR (10×LOQ)
92	ethychlozate	10~200	y=73.761676x+91777.023153	0.9811	20	-50.31	99.47	4.85	109.29	5.37	100.72	9.36	8.23
93	Etofenprox	10~200	y=3458.184606x-1082.084115	0.9936	10	-59.84	111.93	13.8	104.59	9.32	108.84	5.51	5.96
94	Etoxazole	10~200	y=5363.843462x-2170.255052	0.9978	10	-60.71	107.65	7.55	99.24	6.41	104.25	5.66	2.06
95	Etrimfos	10~200	y=4238.521084-2413.593851	0.9962	10	-63.28	94.11	11.53	96.65	9.64	104.29	9.23	3.56
96	Famphur	10~200	y=434.875647x+5425.975353	0.9990	10	-28.08	75.03	8.93	98.35	7.08	101.45	3.98	7.36
97	Fenamidone	10~200	y=6326.476712x-39985.983264	0.9821	10	-50.4	88.51	6.45	104.02	7.05	113.9	1.79	5.96
98	Fenamiphos	10~200	y=73924.481048x-3231.362856	0.9978	10	-58.67	98.04	9.34	110.82	13.02	84.15	6.13	9.01
99	Fenamiphos-sulfone	10~200	y=2037.970944x-6121.027971	0.9972	10	-63.33	79.45	13.57	100.58	11.92	105.41	5.68	4.23
100	Fenamiphos-sulfoxide	10~200	y=28403.368277x-59379.296997	0.9986	10	-52.67	107.98	5.58	87.46	2.69	87.39	12.54	8.16
101	Fenfuram	20~200	y=3905.554250x+16569.249506	0.9994	20	-59.78	100.14	11.11	113.78	5.21	89.07	8.9	8.46
102	Fenhexamid	10~200	y=976.508580x-230.426001	0.9986	10	-53.67	90.88	5.33	96.92	8.58	111.61	2.25	0.47
103	Fenobucarb	10~200	y=9440.002559x-3747.913511	0.9986	10	-54.73	99.41	5.14	91.69	15.15	112.88	14.68	5.55
104	Fenothiocarb	10~200	y=16362.072948x-2424.518205	0.9972	10	-64.94	111.12	5.01	104.19	7.23	112.76	8.28	6.35
105	Fenoxycarb	10~200	y=13425.836138x-2414.273548	0.9956	10	-26.04	90.35	8.35	96.37	7.08	101.45	6.92	5.94
106	Fenpiclonil	10~200	y=251850.718349x-24221.526819	0.9978	10	179.1	94.79	6.58	103.91	5.34	107.54	3.65	5.12
107	Fenpyrazamine	10~200	y=5368.676714x+8443.636926	0.9946	10	-63.95	94.53	7.02	118.56	5.34	106.88	3.69	4.60
108	Fenpyroximate-(E)	10~200	y=141738.092818x-1524.290517	0.9962	10	-49.13	100.63	9.35	102.78	4.17	101.49	2.84	4.96
109	Fenpyroximate-(Z)	10~200	y=3535.088088x-15553.028857	0.9944	10	-49.25	96.79	5.34	112.86	5.74	113.5	2.34	3.19
110	Fenuron	10~200	y=1759.669168x+169.187161	0.9984	10	-58.4	82.71	15.49	74.46	14.58	93.27	5.56	1.47

No.	Compounds	Linear range （μg/kg）	Linearequation	R^2	LOQ (μg/kg）	Matrix effect (%)	Recovery (LOQ)	RSD (LOQ)	Recovery (2×LOQ)	RSD (2×LOQ)	Recovery (10×LOQ)	RSD (10×LOQ)	RSDwR (10×LOQ)
111	Ferimzone-(E)	10~200	y=572.280309x-1800.629824	0.9805	10	-64.93	110.87	18.13	102.14	5.27	105.6	3.5	4.31
112	Ferimzone-(Z)	10~200	y=5191.703920x-3052.050675	0.9984	10	-64.09	106.11	6.11	103.06	8.32	107.89	4.5	6.23
113	Ferulic acid	500-10000	*y*= 66005.904715X+7362.794694	0.9976	50	-8.32	96.56	6.62	98.32	4.61	99.74	5.84	3.02
114	Flamprop-isopropyl	10~200	y=2341.536814x+2463.582042	0.9862	10	-35.25	93.75	8.03	99.19	8.46	106.45	3.46	3.40
115	Flonicamid	10~200	y=3039.793673x-4738.174604	0.9978	10	-43.86	77.14	9.07	104.5	7.93	107.42	7.44	4.02
116	Florasulam	10~200	y=4252.524142x-35635.673796	0.9988	10	-38.23	89.42	7.43	90.38	5.36	104.35	8.56	2.67
117	Fluacrypyrim	20~200	y=52287.474566x-68250.410610	0.9984	20	-60	111.02	10.16	102.96	3.32	84.9	10.75	7.01
118	Fluazifop-butyl	10~200	y=15186.416107x-29605.884297	0.9986	10	-66.03	75.34	5.92	96.64	10.51	115.53	5.13	6.99
119	Flufenacet	10~200	y=2322.708611x-587.143396	0.9978	10	-63.69	114.37	5.96	98.46	4.8	108.88	2.71	3.14
120	Flufenoxuron	10~200	y=1343.355884x+5937.009814	0.9950	10	-54.82	114.11	5.61	112.5	4.62	108.57	2.41	5.23
121	Flufenpyr-ethyl	20~200	y=570.526363x-3532.886118	0.9922	20	-61.97	110.99	6.99	116.57	1.4	94.97	3.78	1.53
122	Flufiprole	10~200	y=769.174301x-1298.962052	0.9974	10	-50.56	103.97	8.07	105.72	12.52	110.32	6.34	4.55
123	Flumetsulam	10~200	y=957.624965x-3653.745754	0.9902	10	-32.52	90.36	6.85	99.34	5.84	102.84	6.91	8.93
124	Flumiclorac-pentyl	20~200	y=349.603466x-1900.313037	0.9807	20	-65.58	118.99	15.47	117.54	16.34	108.78	12.78	8.34
125	Fluopyram	10~200	y=1949.444763x-945.398301	0.9972	10	-60.35	95.58	6.35	110.39	4.28	107.06	3.17	8.95
126	Fluoroglycofen-ethyl	10~200	y=3352.187386x-6185.399299	0.9996	10	-53.68	103.23	10.78	111.5	5.9	108.31	5.78	5.60
127	Fluquinconazole	20~200	y=167.760471x-1154.020205	0.9946	20	-62.55	104.77	8.21	109.54	4.23	107.47	7.81	5.20
128	fluroxypyr-meptyl	10~200	y=9277.761003x-13334.431869	0.9974	10	-64.4	77.92	9.98	102.89	13.46	105.27	4.49	2.36
129	Flurprimidol	10~200	y=1661.317022x-1091.411842	0.9960	10	-49.03	98.99	6.56	108.51	3.88	107.69	2.83	3.13

No.	Compounds	Linear range（μg/kg）	Linearequation	R^2	LOQ (μg/kg)	Matrix effect (%)	Recovery (LOQ)	RSD (LOQ)	Recovery (2×LOQ)	RSD (2×LOQ)	Recovery (10×LOQ)	RSD (10×LOQ)	RSDwR (10×LOQ)
130	Flusilazole	10~200	y=931.089660x-4740.391790	0.9900	10	-58.58	90.98	12.73	111.49	4.23	103.27	1.97	5.35
131	Flutolanil	10~200	y=235.457146x+625.725759	0.9823	30	-35.90	80.24	8.90	88.90	9.35	102.86	8.90	1.25
132	Flutriafol	10~200	y=5321.491444x+601.383514	0.9990	10	-56.57	95.14	9.12	90.74	4.61	115.33	3.44	4.19
133	Fluxapyroxad	20~200	y=146.900685x-1761.013816	0.9884	20	-58.5	111.71	3.36	112.15	2.93	88.98	7.93	6.35
134	Fonofos	20~200	y=346265.673600x-25786.340841	0.9926	20	-28.34	78.34	7.67	90.54	8.46	99.06	6.97	7.42
135	Fosthiazate	10~200	y=297.524670x-1439.232628	0.9996	10	-47.12	99.24	14.2	98.99	4.33	107.24	4.21	1.12
136	Furalaxyl	10~200	y=344.863561x-175.004679	0.9864	10	-34.24	88.92	8.46	99.78	9.73	90.57	7.78	5.75
137	Furathiocarb	10~200	y=911.172318x-4082.629366	0.9932	10	-26.49	90.27	6.08	100.77	6.31	111.65	1.77	5.03
138	Furmecyclox	20~200	y=6095.195013x-31765.687669	0.9829	20	-63.47	87.83	10.06	97.55	16.59	79.04	10.64	6.30
139	Griseofulvin	10~200	y=8276.487139x-4412.852002	0.9966	10	-41.32	83.96	3.85	89.05	7.36	102.76	9.26	5.16
140	Haloxyfop-2-ethoxyethyl	10~200	y=12575.578352x-23616.068329	0.9930	10	-29.05	73.89	6.34	101.78	8.57	103.8	8.56	3.82
141	Hexazinone	10~200	y=2206.659667x+3778.268675	0.9976	10	-50.58	110.62	3.06	109.91	5.11	106.35	4.34	2.72
142	Imazamethabenz-methyl	10~200	y=2641.961022x-9125.862721	0.9853	10	-49.11	113.81	4.25	98.6	2.76	109.1	3.53	7.34
143	Imicyafos	10~200	y=1727.416714x-624.778510	0.9896	10	-50.51	95.97	6.52	109	3.38	114.58	2.55	9.25
144	Indanofan	10~200	y=8423.975785x-15356.542464	0.9886	10	-21.53	80.03	7.94	95.90	6.03	93.67	4.67	8.87
145	Iodosulfuron-methyl	10~200	y=44899.521495x-283264.000294	0.9837	10	-3.07	98.86	4.16	95.91	3.96	108.4	1.63	0.56
146	Ipconazole	10~200	y=16071.767473x+3836.072804	0.9984	10	-47.19	107.31	4.79	104.64	4.82	102.69	4.15	5.79
147	Isazofos	10~200	y=600.209201x-177.484875	0.9976	10	-58.59	92.3	13.45	78.4	8.53	115.92	7.45	5.41

No.	Compounds	Linear range （μg/kg）	Linearequation	R^2	LOQ (μg/kg)	Matrix effect (%)	Recovery (LOQ)	RSD (LOQ)	Recovery (2×LOQ)	RSD (2×LOQ)	Recovery (10×LOQ)	RSD (10×LOQ)	RSDwR (10×LOQ)
148	Isocarbamid	10~200	y=3819.966555x-49565.038889	0.9898	10	-43.6	112.87	3.77	110.06	2.27	113.06	1.44	4.49
149	isocarbophos	30~200	y=1206.088499x-1187.008468	0.9976	30	-46.67	71.78	2.28	93.97	8.3	97.39	5.66	5.53
150	Isofenphos-Methyl	10~200	y=4525.567145x-4572.737626	0.9902	10	-25.78	82.79	6.03	93.57	6.89	90.86	9.03	8.64
151	Isomethiozin	10~200	y=1454.565257x-4343.576245	0.9930	10	-36.02	85.47	5.04	99.05	9.05	97.56	7.13	8.61
152	Isoprothiolane	10~200	y=19831.120729x+17523.690756	0.9966	10	-62.26	90.74	17.82	116.29	3.56	110.65	2.26	0.31
153	Isoproturon	10~200	y=49483.187229x-80086.448757	0.9976	10	-54.02	105.83	4.31	114.1	4.84	108.39	2.82	3.55
154	Isoxadifen-ethyl	30~200	y=1130.381722x-2220.884789	0.9980	30	-63.68	93.5	18.3	103.78	12.05	84.5	10.84	7.82
155	Karbutilate	10~200	y=4573.971287x-21353.561209	0.9986	10	-15.03	88.94	8.57	102.67	10.75	89.46	6.95	8.86
156	Ligustilide	250-10000	y=18342.424081X+1031.281931	0.9972	250	-30.23	86.34	6.34	94.68	4.93	99.04	3.77	2.05
157	Mefenacet	10~200	y=28872.319421x-14130.296957	0.9980	10	-50.61	96.27	9.28	112.1	5.43	114.54	3.29	6.10
158	Mefenpyr-diethyl	10~200	y=338.520338x-388.965472	0.9984	10	-60.5	104.83	4.29	109.55	5.14	113.53	2.57	3.09
159	Mepronil	10~200	y=3670.564908x+3188.032679	0.9940	10	-59.28	97.81	8.74	112.27	5	110.67	3.18	5.71
160	Metaflumizone-(E)	10~200	y=1543.340052x-3984.366668	0.9976	10	-23.06	75.86	9.03	95.52	7.94	90.72	8.35	7.83
161	Metaflumizone-(Z)	20~200	y=3242.875123x+2901.837264	0.9868	20	-36.06	80.57	7.78	83.15	8.03	93.78	9.03	7.03
162	Metalaxyl	10~200	y=1131.174628x-2324.986468	0.9954	10	-19.54	74.78	12.67	90.45	6.78	90.24	7.27	7.70
163	Metamitron	10~200	y=4638.088251x+2502.415681	0.9988	10	-3.34	78.4	18.22	85.84	2.81	87.91	2.89	1.74
164	Metazachlor	10~200	y=6891.894140x-24901.450919	0.9962	10	-58.91	108.61	10.27	110.09	9.94	107.15	6.1	9.04
165	Metconazole	10~200	y=2392.841940x-13249.951492	0.9964	10	-63.13	110.61	14.79	87.4	6.51	78.37	14.89	9.21
166	Methamidophos	30~200	y=4619.365290x+1696.143353	0.9992	30	-57.33	88.79	7.42	83.09	9.03	94.17	7.16	8.58

No.	Compounds	Linear range （μg/kg）	Linearequation	R^2	LOQ (μg/kg)	Matrix effect (%)	Recovery (LOQ)	RSD (LOQ)	Recovery (2×LOQ)	RSD (2×LOQ)	Recovery (10×LOQ)	RSD (10×LOQ)	RSDwR (10×LOQ)
167	Methfuroxam	10~200	y=5169.824135x-23386.410270	0.9888	10	-26.07	82.67	8.90	79.93	8.94	108.46	8.67	9.90
168	Methoprotryne	10~200	y=2173.675960x-1052.740197	0.9982	10	-49.98	98.54	5.39	100.31	3.01	116.26	2.11	2.55
169	Metobromuron	10~200	y=2170.314085x-3358.210812	0.9984	10	-57.78	101.1	15.49	118.56	7.09	117.98	5.85	8.05
170	Metribuzin	20~200	y=18444.477084x+4580.909302	0.9974	20	-64.29	96.77	8.83	101.76	4.83	107.47	8.42	0.64
171	Metsulfuron-methyl	10~200	y=229.788260x+43037.696065	0.9817	20	-22.31	99.26	4.24	106.68	2.62	83.72	6.82	9.73
172	Mevinphos-(E)	10~200	y=20808.311078x+35582.275927	0.9972	10	-61.59	112.22	11.02	110.21	5.58	116.46	4.05	0.83
173	Mevinphos-(Z)	10~200	y=16543.158213x-18845.918134	0.9968	10	-54.44	87.12	7.39	99.64	4.61	111.18	3.21	1.00
174	Monocrotophos	20~200	y=2836.847351x-7623.604369	0.9982	20	-38.67	108.7	6.03	75.34	5.69	82.94	9.68	4.55
175	Monolinuron	20~200	y=21204.626656x+40580.476836	0.9966	20	-61.35	106.29	10.24	112.31	3.93	111.76	6.36	9.02
176	Monuron	10~200	y=7924.828636x+4305.099090	0.9988	10	-59.46	102.97	11.25	111.73	4.89	112.92	3.02	6.59
177	Myclobutanil	10~200	y=4326.787698x-3078.109261	0.9972	10	-46.42	103.85	6.22	104.11	6.3	118.28	4.34	7.44
178	Neburon	10~200	y=16257.802386x+2722.495677	0.9968	10	-63.76	96.45	10.53	106.54	14.35	111.47	8.18	8.60
179	Norflurazon	10~200	y=8860.264122x-20430.402872	0.9990	10	-56.42	98.45	7.26	113.52	5.77	115.69	5.33	3.55
180	Noruron	10~200	y=2674.432902x+5575.963723	0.9843	10	-53.24	106.16	5.64	112.2	4.77	115.43	3.89	3.13
181	Novaluron	10~200	y=1865.178065x+8113.021502	0.9922	10	-48.59	82.48	5.68	114.76	2.87	107.55	2.89	4.04
182	Nuarimol	10~200	y=18936.589150x-3489.882306	0.9924	10	194.52	106.88	6.39	110.24	8.43	116.77	4.55	7.45
183	Ofurace	10~200	y=54063.356900x-12174.087689	0.9974	10	-51.35	102.98	4.97	117.25	4.45	116.21	3.82	7.22

No.	Compounds	Linear range （μg/kg）	Linearequation	R^2	LOQ (μg/kg)	Matrix effect (%)	Recovery (LOQ)	RSD (LOQ)	Recovery (2×LOQ)	RSD (2×LOQ)	Recovery (10×LOQ)	RSD (10×LOQ)	RSDwR (10×LOQ)
184	Orbencarb	10~200	y=783.506312x+646.713191	0.9906	10	-44.94	106.97	6.15	103.46	3.49	108.7	1.84	5.83
185	oxadiazon	10~200	y=2038.206834x+1139.977837	0.9944	10	-65.19	92.54	7.91	114.95	6.68	110.18	5.7	9.13
186	Oxadixyl	10~200	y=4777.796334x-5643.052581	0.9986	10	-52.18	93.93	8.77	107.43	4.23	110.73	3.34	0.70
187	Oxasulfuron	10~200	y=15390.391045x-3421.849150	0.9928	10	-39.68	99.87	8.32	99.16	8.6	105.46	4.24	3.19
188	Oxycarboxin	10~200	y=551.147950x-1419.542984	0.9968	10	-53.57	115.35	13.72	112.66	9.12	116.96	4.11	7.63
189	oxydemeton-methyl	20~200	y=10865.985132x-5419.435875	0.9978	20	-35.72	90.36	9.05	77.04	3.67	105.67	11.67	8.52
190	Pebulate	10~200	y=5108.057347x+42825.012361	0.9932	10	-34.58	95.44	4.97	107.56	4.1	117.4	3.23	7.71
191	Penconazole	10~200	y=18927.895427x-56498.632132	0.9934	10	-30.85	78.52	6.78	90.04	7.90	102.40	9.04	5.85
192	Penflufen	10~200	y=16730.162586x-36038.777221	0.9974	10	-60.4	108.65	4.78	113.86	18.98	119.51	2.99	6.16
193	Penoxsulam	10~200	y=31878.699070x-15924.818273	0.9978	10	-33.67	95.39	4.61	100.97	3.9	111.52	1.92	3.96
194	Pentanochlor	30~200	y=5624.791289x+2291.145522	0.9964	30	-60.47	83.37	10.21	94.29	6.46	82.87	7.83	1.25
195	Pethoxamid	10~200	y=8431.519021x+28587.409493	0.9982	10	-59.46	84.72	11.05	102.39	10.96	110.07	6.04	8.91
196	Phorate-oxon-sulfone	10~200	y=4377.032682x-4917.049969	0.9970	10	-62.42	106.62	11.09	118.17	6.12	119.3	4.02	0.84
197	Phorate-oxon-sulfoxide	10~200	y=3329.631777x+5084.977326	0.9946	10	-56.51	102.25	13.34	112.79	6.44	110.26	10.09	6.33
198	Phorate-Sulfone	10~200	y=10643.003622x-41505.036362	0.9986	10	-52.67	94.31	15.24	104.45	5.93	79.22	6.38	2.85
199	Phorate-Sulfoxide	10~200	y=29053.560314x-67681.588618	0.9986	10	-33.33	73.72	5.51	89.57	15.47	74.89	12.27	9.08
200	Phosfolan	10~200	y=49250.510951x-7151.901593	0.9918	10	-29.03	74.90	8.67	102.78	8.46	99.03	7.83	5.69

No.	Compounds	Linear range （μg/kg）	Linearequation	R^2	LOQ (μg/kg)	Matrix effect (%)	Recovery (LOQ)	RSD (LOQ)	Recovery (2×LOQ)	RSD (2×LOQ)	Recovery (10×LOQ)	RSD (10×LOQ)	RSDwR (10×LOQ)
201	Phosfolan-Methyl	20~200	y=29361.159062x-6181.619051	0.9930	20	-39.34	89.36	9.05	89.25	7.83	98.89	2.56	3.00
202	Phosphamidon-(E)	20~200	y=5214.222199x+23354.580267	0.9976	20	-52.5	96.06	2.74	110.46	2.72	101.87	4.82	9.52
203	Phosphamidon-(Z)	20~200	y=6222.881717x-29409.271182	0.9910	20	-51.23	112.42	11.23	101.16	3.73	82.06	6.62	8.52
204	Phoxim	10~200	y=24892.671034x-3098.519820	0.9978	10	-3.76	108.39	5.69	98.86	1.31	105.22	2.55	5.92
205	Piperonyl Butoxide	10~200	y=7269.691589x+4351.619028	0.9950	10	-59.25	101.63	7.98	108.62	1.23	113.99	2.44	7.01
206	Promecarb	10~200	y=23320.896122x-5426.975353	0.9956	10	-31.68	79.78	11.06	91.06	9.46	103.85	4.82	3.60
207	Prometryn	10~200	y=21725.716902x-2661.451782	0.9942	10	-60.63	110.1	9.02	95.9	18.62	83.25	4.25	0.45
208	Propachlor	10~200	y=6753.875364x-43512.008575	0.9942	10	-25.36	83.67	10.97	99.57	10.05	90.78	6.45	7.92
209	Propazine	20~200	y=14597.030066x-25839.470297	0.9980	20	-53.81	115.41	11.06	106.7	4.52	80.73	8.31	3.96
210	Propisochlor	10~200	y=239.453410x-714.002447	0.9908	10	-59.93	113.95	6.45	84.05	16.72	97.98	6.79	8.89
211	Propyzamide	10~200	y=362.269005x-2013.192235	0.9980	10	-54.51	112.96	15.38	112.11	5.03	108.08	8.4	5.18
212	Prosulfocarb	20~200	y=30583.025349x-103407.292909	0.9968	20	-64.93	117.08	17.03	111.89	5.44	106.46	8.35	4.24
213	Prosulfuron	10~200	y=5099.140789x+11331.887994	0.9964	10	-41.88	118.44	5.29	102.36	3.91	116.32	1.15	0.65
214	Prothiofos	10~200	y=13243.096590x-132319.09895	0.9948	10	-31.53	79.78	9.05	105.95	9.57	88.92	8.25	0.83
215	Prothoate	10~200	y=17064.444302x-113536.171346	0.9980	10	-48.42	99.8	6.5	111.54	7.02	116.23	5.12	4.48
216	Pyracarbolid	10~200	y=727.598617x+5041.479276	0.9811	30	-50.32	113.78	4.51	107.94	3.7	97.04	5.85	5.02
217	Pyraclostrobin	10~200	y=236.532943x-1762.176277	0.9956	10	-61.83	106.79	7.67	117.83	4.79	110.16	3.03	4.25
218	Pyraflufen-ethyl	10~200	y=1024.413517x-348.856889	0.9978	10	-62.56	103.28	8.62	114.36	8.03	112.39	5.72	6.92

No.	Compounds	Linear range （μg/kg）	Linearequation	R^2	LOQ (μg/kg）	Matrix effect (%)	Recovery (LOQ)	RSD (LOQ)	Recovery (2×LOQ)	RSD (2×LOQ)	Recovery (10×LOQ)	RSD (10×LOQ)	RSDwR (10×LOQ)
219	Pyrametostrobin	10~200	y=1885.434844x-2292.544955	0.9988	10	-56.47	81.94	7.67	102.89	1.88	110.78	4.27	4.69
220	Pyrazosulfuron-ethyl	10~200	y=14369.442889x-10164.062228	0.9978	10	-51.64	107.17	3.38	107.2	3.71	116.23	2.22	6.14
221	Pyrazoxyfen	10~200	y=5385.923258x-10319.206045	0.9980	10	-60.05	103.85	5.79	117.6	5.41	118.65	2.3	9.96
222	Pyributicarb	10~200	y=1162.836338x-951.739424	0.9984	10	-64.92	104.95	6.4	106.88	6.42	106.2	1.83	1.05
223	Pyridaphenthion	10~200	y=9901.471851x-26534.748163	0.9968	10	-52.49	94.52	3.05	106.56	3.66	115.32	4.34	2.03
224	Pyriftalid	10~200	y=4798.527725x-6071.627960	0.9974	10	-51.65	95.71	5.76	107.98	5.36	116.02	2.88	1.17
225	Pyriminobac-Methyl-(E)	10~200	y=1265.624680x-1224.576058	0.9994	10	-46.67	96.15	11.89	97.25	14.59	107.47	2.38	3.04
226	Pyriminobac-Methyl-(Z)	10~200	y=12870.824786x-25241.007318	0.9986	10	-65.33	70.46	13.75	96.46	4.59	80.72	5.28	9.25
227	Pyrimitate	10~200	y=8631.701463x-22027.783065	0.9980	10	-59.66	107.38	4.41	108.38	4.37	112.64	2.95	9.13
228	Pyriproxyfen	20~200	y=49727.534314x-130639.153265	0.9980	20	-64.85	100.58	9.65	115.54	2.19	92.47	7.48	4.82
229	Pyrisoxazole	10~200	y=35976.875809x-120965.657120	0.9950	10	-24.90	77.35	12.78	98.34	8.03	90.67	5.71	6.94
230	Pyroquilon	10~200	y=15303.657040x-28906.044492	0.9988	10	-58.49	90.83	6.94	104.86	5.14	103.42	3.68	3.91
231	Sebuthylazine-desethyl	10~200	y=78488.565174x-186020.427928	0.9982	10	-53.98	74.96	11.06	95.3	13	105.6	6.24	7.75
232	Sedaxane	10~200	y=11489.245377x+6188.312771	0.9918	10	-54.67	90.64	8.68	95.95	8.28	109.57	9.69	4.18
233	Siduron	10~200	y=400.348073x-328.014765	0.9966	10	-60.26	77.14	4.72	95.76	12.46	115.27	13.8	8.82

No.	Compounds	Linear range （μg/kg）	Linearequation	R^2	LOQ (μg/kg）	Matrix effect (%)	Recovery (LOQ)	RSD (LOQ)	Recovery (2×LOQ)	RSD (2×LOQ)	Recovery (10×LOQ)	RSD (10×LOQ)	RSDwR (10×LOQ)
234	Silthiofam	10~200	y=3734.321720x-8927.593302	0.9956	10	-62.04	95.39	8.06	106.9	9.15	113.85	5.05	0.47
235	Sulfometuron-Methyl	10~200	y=2681.236803x+920.256574	0.9998	10	-60.04	109.59	4.81	115.03	5	107.14	3.33	4.98
236	Sulfotep	10~200	y=61.899484x-674.666255	0.9978	10	-53.33	100.47	11.57	102.79	9.75	72.85	7.04	7.29
237	Sulprofos	10~200	y=21178.229793x-55410.085832	0.9974	10	-65.39	116.18	16.6	104.36	13.82	113.19	6.24	8.54
238	Tebuconazole	10~200	y=2283.193158x+495.780532	0.9952	10	-54.64	110.42	5.31	119.46	5.52	110.32	3.97	6.02
239	Tebufenpyrad	10~200	y=13636.880439x+2398.131427	0.9988	10	-60.64	89.85	6.6	116.19	6.87	118.99	5.82	0.87
240	Tebupirimfos	10~200	y=18659.216564x-89709.761296	0.9952	10	-35.35	80.78	10.06	90.86	12.56	105.95	9.58	9.84
241	Tebutam	10~200	y=3595.387893x-8722.418044	0.9984	10	-64.36	116.01	5.51	105.28	5.99	110.35	1.62	7.71
242	Tebuthiuron	10~200	y=7152.092891x-16262.619036	0.9841	10	-58.15	99.12	5.87	109.86	5.8	109.24	3.32	8.53
243	Temephos	20~200	y=104.348972x+32.887620	0.9857	20	-65.5	107.77	13.59	114.52	7.12	89.36	4.17	1.96
244	Tepraloxydim	10~200	y=254.070859x-1183.166491	0.9920	10	-62.94	98.56	7.17	93.25	7.27	94.96	10.58	5.14
245	Terbucarb	10~200	y=3884.350603x+814.601093	0.9962	10	-53.78	82.2	4.79	107.47	10.98	104.02	5.2	5.31
246	Terbufos	10~200	y=10975.763143x-34126.812656	0.9952	10	-23.31	93.78	9.05	96.79	9.46	90.64	6.25	7.12
247	Terbufos-Sulfone	10~200	y=3353.319625x+1281.112309	0.9924	10	-47.33	101.93	4.78	73.68	9.59	101.83	10.74	2.13
248	Terbufos-Sulfoxide	10~200	y=9165.585952x-2529.353166	0.9966	10	-58.67	76.47	10.53	80.29	5.13	93.62	12.37	7.22
249	Terbumeton	10~200	y=162.283371x-1255.246616	0.9986	10	-48.77	103.28	6.21	109.58	6.87	108.67	2.82	8.59
250	Terbuthylazine	10~200	y=87.099114x+37160.202605	0.9803	30	-57.66	110.66	2.47	100.86	4.89	88.93	2.04	9.15
251	Tetraconazole	10~200	y=15290.189058x-81562.178255	0.9944	10	-58.95	99.26	8.91	112.14	5.04	111.89	2.73	2.55

No.	Compounds	Linear range （μg/kg）	Linearequation	R^2	LOQ (μg/kg)	Matrix effect (%)	Recovery (LOQ)	RSD (LOQ)	Recovery (2×LOQ)	RSD (2×LOQ)	Recovery (10×LOQ)	RSD (10×LOQ)	RSDwR (10×LOQ)
252	Thenylchlor	10~200	y=15351.618251x-6152.826914	0.9960	10	-63.97	83.23	6.88	102.71	9.71	111.75	5.12	6.78
253	Thiazafluron	10~200	y=43406.666025x-102702.885980	0.9984	10	-61.99	85.09	3.26	109.23	5.56	107.29	4.97	6.26
254	Thiazopyr	10~200	y=5473.847655x+6194.922273	0.9984	10	-62.46	103.03	3.83	110.85	5.55	110.15	1.59	3.94
255	Thiobencarb	10~200	y=2676.224992x-1265.262079	0.9992	10	-61.36	100.06	3.75	111.67	4.52	111.96	3.11	2.65
256	Thiodicarb	10~200	y=579.790566x-816.184806	0.9980	10	-42.19	73.39	10.35	73.45	9.45	72.46	4.53	5.77
257	Thionazin	10~200	y=6282.287132x-22577.920591	0.9978	10	-30.02	81.06	12.19	98.1	12.36	107.43	4.75	9.09
258	Tiocarbazil	10~200	y=1905.384003x+415.704760	0.9982	10	-66.39	74.13	10.29	106.81	15.84	101.16	3.33	8.91
259	Tolclofos-methyl	10~200	y=2028.769821x+4622.722056	0.9994	10	-60.97	101.59	7.65	110.1	6.39	109.45	4.07	7.72
260	Tolfenpyrad	10~200	y=18754.120954x-117642.865320	0.9962	10	-38.04	84.27	8.05	103.95	12.67	95.72	7.86	0.52
261	Triadimefon	10~200	y=346.527871x+1475.917162	0.9878	10	-55.11	105.75	11.74	84.04	16.58	104.46	8.34	4.05
262	Triadimenol	10~200	y=33472.596395x+154250.456317	0.9980	10	-58.67	105.87	14.42	105.35	6.33	114.05	3.99	5.09
263	Triamiphos	10~200	y=16935.306395x-5535.939701	0.9986	10	-47.46	82.71	11.27	93.99	7.41	101.63	1.99	7.84
264	Triasulfuron	10~200	y=1253.536388x-1881.610203	0.9998	10	-30.38	98.5	7.66	113.01	6.45	115.98	4.24	1.22
265	Triazophos	10~200	y=6830.375981x+7060.974534	0.9950	10	-60.56	88.3	14.76	86.38	9.8	112.29	3.59	7.32
266	Tribufos	10~200	y=3500.756169x-8497.384690	0.9978	10	-65.88	106.94	13.38	106.43	3.29	106.13	3.2	1.11
267	Trietazine	10~200	y=8978.643328x-24945.060121	0.9972	10	-61.5	84.81	6.74	93.52	8.87	102.5	6.63	1.34
268	Trifloxystrobin	10~200	y=4700.146145x-7039.009688	0.9960	10	-63.3	99.74	5.45	113.15	4.07	98.7	2.26	5.33
269	Triflumizole	10~200	y=5124.846931x-1761.431560	0.9934	10	-51.39	104.64	8.3	109.03	5.75	113.27	4.8	2.68
270	Triticonazole	10~200	y=5346.334697x+41363.340082	0.9990	10	-51.69	78.62	9.69	81.71	13.8	103.89	11.11	8.74

No.	Compounds	Linear range （μg/kg）	Linearequation	R^2	LOQ (μg/kg)	Matrix effect (%)	Recovery (LOQ)	RSD (LOQ)	Recovery (2×LOQ)	RSD (2×LOQ)	Recovery (10×LOQ)	RSD (10×LOQ)	RSDwR (10×LOQ)
271	Uniconazole	10~200	y=5899.263831x-7178.624477	0.9972	10	-55.21	105.66	5.19	113.42	2.79	116.08	2.37	0.66
272	Zoxamide	10~200	y=941.414807x-1714.177487	0.9978	10	-63.14	118.73	5.7	115.1	3.83	116.38	2.21	2.31
